# The rise of baobab trees in Madagascar

**DOI:** 10.1038/s41586-024-07447-4

**Published:** 2024-05-15

**Authors:** Jun-Nan Wan, Sheng-Wei Wang, Andrew R. Leitch, Ilia J. Leitch, Jian-Bo Jian, Zhang-Yan Wu, Hai-Ping Xin, Mijoro Rakotoarinivo, Guy Eric Onjalalaina, Robert Wahiti Gituru, Can Dai, Geoffrey Mwachala, Ming-Zhou Bai, Chen-Xi Zhao, Hong-Qi Wang, Sheng-Lan Du, Neng Wei, Guang-Wan Hu, Si-Chong Chen, Xiao-Ya Chen, Tao Wan, Qing-Feng Wang

**Affiliations:** 1 State Key Laboratory of Plant Diversity and Specialty Crops, Wuhan Botanical Garden, Chinese Academy of Sciences, Wuhan, China; 2 Sino-Africa Joint Research Centre, Chinese Academy of Sciences, Wuhan, China; 3 School of Biological and Chemical Sciences, Queen Mary University of London, London, UK; 4 Royal Botanic Gardens, Kew, London, UK; 5 BGI Genomics, BGI-Shenzhen, Shenzhen, China; 6 Department of Biotechnology and Biomedicine, Technical University of Denmark, Lyngby, Denmark; 7 University of Antananarivo, Antananarivo, Madagascar; 8 Department of Botany, Jomo Kenyatta University of Agriculture and Technology, Nairobi, Kenya; 9 School of Resources and Environmental Science, Hubei University, Wuhan, China; 10 East African Herbarium, National Museums of Kenya, Nairobi, Kenya; 11 Shanghai Chenshan Botanical Garden, Shanghai, China

**Keywords:** Conservation genomics, Plant evolution, Phylogenetics, Biodiversity

## Abstract

The baobab trees (genus *Adansonia*) have attracted tremendous attention because of their striking shape and distinctive relationships with fauna^1^. These spectacular trees have also influenced human culture, inspiring innumerable arts, folklore and traditions. Here we sequenced genomes of all eight extant baobab species and argue that Madagascar should be considered the centre of origin for the extant lineages, a key issue in their evolutionary history^2,3^. Integrated genomic and ecological analyses revealed the reticulate evolution of baobabs, which eventually led to the species diversity seen today. Past population dynamics of Malagasy baobabs may have been influenced by both interspecific competition and the geological history of the island, especially changes in local sea levels. We propose that further attention should be paid to the conservation status of Malagasy baobabs, especially of *Adansonia* *suarezensis* and *Adansonia* *grandidieri*, and that intensive monitoring of populations of *Adansonia* *za* is required, given its propensity for negatively impacting the critically endangered *Adansonia* *perrieri*.

## Abstract (web summary)

We carried out genomic and ecological analyses of all eight extant baobab species, providing insights into their evolutionary history and recommendations for conservation efforts.

## Main

The genus *Adansonia*, better known as the baobabs and ‘mother of the forest’^1^, has captivated botanists, tourists, naturalists and passers-by for centuries. Probably the earliest record of humans marvelling at these amazing trees can be traced back to the Ancient Egyptians, around 2,300 bc (ref. ^1^). With their grotesque appearance, enormous size^1^, reputed longevity^4^ and diverse uses^5^, baobabs have become one of the most charismatic species on our planet. Embedded in folklore and tradition, baobabs have inspired innumerable pieces of art and have been associated with human settlements and cultures over millennia^6,7^.

**Fig. 1:**
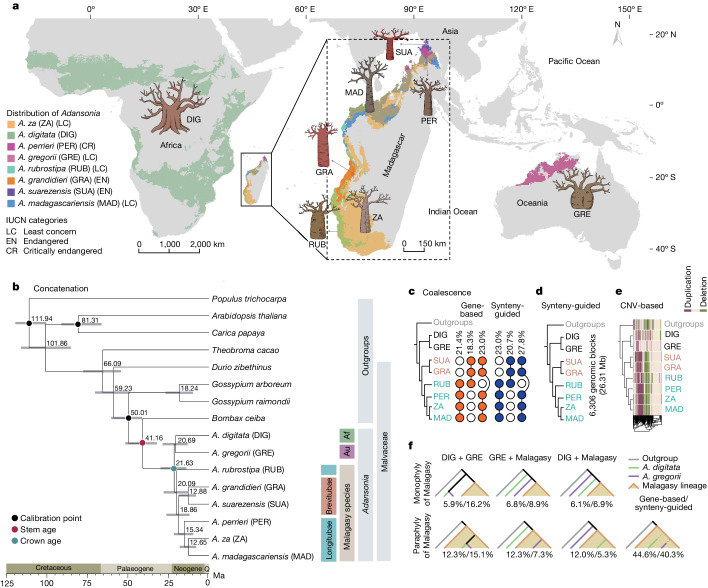
Geographic distribution and phylogenetic relationships between species of *Adansonia.* **a**, The distribution of eight baobab species and their conservation status in IUCN. The coloured patches represent the occurrence of each baobab species. **b**, A maximum-likelihood tree from the concatenation of 999 SCN genes. Median ages of the nodes are shown and a timescale is provided at the bottom; grey horizontal bars show 95% CIs of estimated divergence dates between lineages. The geographic occurrence of the *Adansonia* species is designated: Af, African lineage; Au, Australian lineage and Malagasy species. **c**, Coalescence analyses of 999 concatenated SCN genes. Summary of the proportion of gene tree topologies using either SCN genes (that is, gene-based) or synteny-guided genomic blocks. **d**, Maximum-likelihood phylogenetic relationships of *Adansonia* inferred from analysing concatenated synteny-guided genomic blocks. **e**, The *Adansonia* phylogeny based on CNVs (calculated as a pairwise edit distance of duplications and deletions). *A. gregorii* was selected for the identification of CNVs to circumvent any potential bias caused by using species with a closer phylogenetic relationship as the reference. **f**, The proportions of conflicting maximum-likelihood gene tree topologies. The acronyms used for the species are as in **a**. Numbers represent the percentage of trees with the given topology (SCN gene dataset/synteny-guided genomic blocks). The topologies are sketched to show the different placements of each lineage, regardless of the branch length.

*Adansonia* (family Malvaceae, subfamily Bombacoideae) comprises eight morphologically distinct species^8,9^. The extant geographic distribution of baobabs is unusual, with one tetraploid species, *Adansonia digitata* (2*n* = 4*x* = 168), which is widespread across continental Africa, one diploid species, *Adansonia* *gregorii* (2*n* = 2*x* = 88), restricted to Northwestern Australia, and six diploid species (2*n* = 2*x* = 84, 88) which are endemic to Madagascar^3^ (Fig. 1a). A substantial body of research has detailed the natural history of *Adansonia*, including its taxonomy, ethnobotany, ecology and physiology^1,8^. All but *A. digitata* are listed in The IUCN Red List of Threatened Species, 2023, with three of the Malagasy species threatened with extinction (*Adansonia* *perrieri* classified as critically endangered and *Adansonia* *grandidieri* and *Adansonia* *suarezensis* as endangered). The remaining Malagasy species are now listed as being of least concern but their declining populations indicate that more rigorous conservation strategies are required to ensure the long-term survival of these culturally and globally important species. For that to happen, a detailed understanding of the genetics of baobabs is urgently needed.

Here we present high-quality, chromosomal-level genome assemblies for all eight baobab species (Supplementary Fig. 1). Using these data together with ecological analyses, we uncover the evolutionary history of this genus, including its origin, diversification, population history and patterns of interspecific hybridization. Our findings provide valuable insights for developing effective and biologically informed conservation strategies for these spectacular trees in a changing world^10^.

## The genetic landscape of baobab genomes

Clustering of scaffolds from Hi-C (high-throughput chromosome conformation capture) libraries validated cytological data showing that baobab karyotypes are comprised of numerous small chromosomes (Supplementary Fig. 2). The base chromosome number of *n* = 44 was conserved in all diploid *Adansonia* species, except *A. perrieri*, consistent with previous counts for *Adansonia*^11^. The chromosome count of *A. perrieri* (*n* = 42), was validated by the Hi-C contact map and syntenic alignment to *A. grandidieri* (*n* = 44) (Supplementary Fig. 3).

Macrosynteny alignments revealed high levels of synteny between species (Supplementary Fig. 4a and Supplementary Table 1) and suggest that chromosomal rearrangements and fusions occurring in the lineage leading to *A. perrieri* (Supplementary Fig. 4b) gave rise to its reduced chromosome number. Alignments of *A. perrieri* to outgroup Malvaceae species *Theobroma cacao* (2*n* = 20) and *Gossypium raimondii* (2*n* = 26) showed several scattered syntenic blocks in *A. perrieri* (Supplementary Fig. 4c).

Analysis of total coverage of *K-*mer pairs indicates that *A. digitata* is an autotetraploid (Supplementary Fig. 5). Although analyses of synonymous substitutions per synonymous site (*K*_s_) in paralogous gene pairs failed to identify any further whole-genome duplications (WGD) with the divergence of *Adansonia*, evidence of a shared ancient WGD that is either Malvaceae-specific or specific to the common ancestor of Bombacoideae/Malvoideae (Supplementary Fig. 6), confirmed earlier findings^12^. The higher chromosome numbers in Bombacoideae compared with most other species in Malvaceae^13,14^ may reflect this ancestral WGD, with reduced numbers in Malvoideae arising from fusions in the lineage leading to that tribe. However, chromosome rearrangements and fissions in the lineage leading to Bombacoideae may also have given rise to their high chromosome numbers. See Supplementary Note 1 for a summary of genome size diversity in *Adansonia* and Malvaceae.

## The *Adansonia* phylogeny

The eight extant baobab species form three geographic lineages, *A. digitata* on continental Africa, *A. gregorii* in Northwestern Australia and the remainder in Madagascar^3^. Owing to the limited number of loci used in previous phylogenetic studies^3^, the interspecific relationships between *Adansonia* species have remained controversial, making it difficult to infer patterns of geographic divergence and the centre of origin for the three extant lineages.

To dissect the evolutionary history of baobabs, we analysed our whole-genome sequence assemblies for the eight baobabs with eight outgroup species (five species from Malvaceae and three from other families; Supplementary Table 2). Concatenated datasets of 999 single-copy nuclear (SCN) genes were extracted to establish a phylogenetic framework for *Adansonia*. The highest proportion of maximum-likelihood trees indicated that *A. digitata* and *A. gregorii* form a sister clade to the Malagasy lineage (Fig. 1b and Supplementary Fig. 7a). Secondary calibrated molecular clocks indicated a stem lineage origin of *Adansonia* at about 41.1 (95% confidence interval (CI) 52.0–32.4) million years ago (Ma), far younger than the break-up of Gondwana (about 160 Ma)^15^ (Fig. 1b), supporting previous analyses^2^. The diversification of Malagasy baobabs probably occurred over the last 20.6 (95% CI 28.8–13.3) to 12.6 (95% CI 18.2–8.0) Myr (Fig. 1b). There was partial support for the two morphologically defined sections Brevitubae and Longitubae^8^, but the results placed *Adansonia* *rubrostipa* as sister to the two sections, indicating paraphyly of Longitubae (Fig. 1b). However, in coalescent-based species trees of the 999 SCN genes by ASTRAL (Supplementary Fig. 8a), monophyly of Longitubae was observed in 21.4% of the trees recovered, occurring without topological consensus for the placement of *A. rubrostipa* (Fig. 1c).

**Extended Data Fig. 1:**
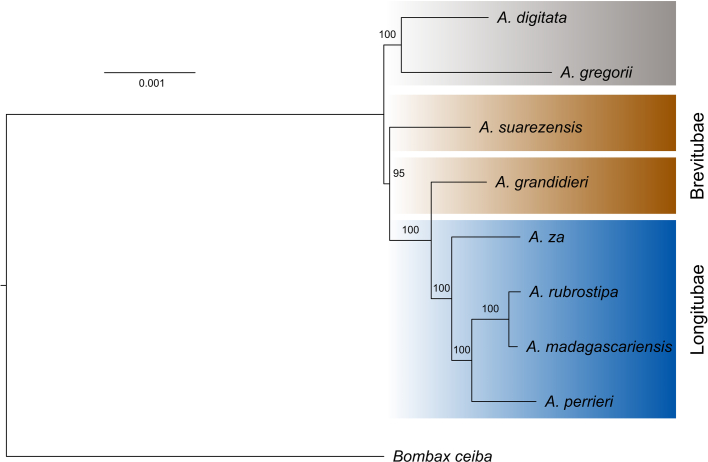
Plastid genome tree inferred from maximum-likelihood phylogenetic analysis. There was some discordance between nuclear and plastid phylogenies in the placement of *A. suarezensis*, perhaps because it has retained a different ancestral plastome than the other species.

To circumvent potential bias brought about by natural selection on non-neutral gene sites, we also conducted a synteny-guided phylogenomic analysis of all baobab species and *G. raimondii*, an approach that incorporated more non-coding DNA. The analysis used a concatenated block of 6,306 highly conserved syntenic segments (≥3 kilobases (kb), synteny-guided genomic blocks) which were assembled into a 26.31 megabase (Mb) fragment. The maximum-likelihood tree supported a topology with *A. digitata* + *A. gregorii* being sister to the rest of the baobabs and monophyly of sections Brevitubae and Longitubae (Fig. 1d and Supplementary Fig. 7b). However, monophyly of Longitubae was not recovered in coalescent-based species trees using these synteny-guided genomic blocks (Supplementary Fig. 8b). Similar results were observed when using small (2 kb) alignment blocks to reduce any potential influence from intralocus recombination breakpoints (Supplementary Fig. 9). We extended our phylogenetic analyses to use copy number variations (CNVs) (Supplementary Table 3) which are less likely to occur independently in separate lineages than DNA polymorphisms (for example, single-nucleotide polymorphisms) and are valuable markers for phylogenetic reconstructions^16^. The neighbour-joining tree of CNV genotypes (Fig. 1e) recapitulated the phylogenetic relationships seen with the concatenated synteny-guided genomic blocks (Fig. 1d) and many of the coalescence gene trees (Fig. 1c), leading to an emerging consensus of the *Adansonia* phylogeny comprising monophyly of sections Brevitubae and Longitubae. Furthermore, we revisited the plastid phylogeny^8^ and showed a well-supported Longitubae clade (Extended Data Fig. 1) but with some discordance between nuclear and plastid phylogenies in the placement of *A. suarezensis*, perhaps because the latter retains a different ancestral plastome than the other species.

Despite an overall consensus on the monophyly of the Malagasy lineage, we observed a large proportion of SCN gene trees (81.2%) and synteny-guided genomic block trees (68.1%) which showed paraphyly of the Malagasy baobabs (Fig. 1f). We also noticed that more trees supported a clade containing *A. gregorii* and the Malagasy baobabs as sister to *A. digitata* than a clade containing *A. digitata* and Malagasy baobabs as sister to *A. gregorii* (19.1% versus 18.1% in SCN gene trees and 16.2% versus 12.2% in synteny-guided genomic block trees) (Fig. 1f). Such genome-wide phylogenetic discordances indicate a complex scenario of reticulations in baobabs^3^, involving introgression and/or incomplete lineage sorting (ILS) of ancestral variation^17,18^.

## Gene flow among baobab species

To explore the impact of introgression and ILS on the *Adansonia* phylogeny, we used three approaches: (1) Patterson’s *D*-statistics and *f*-branch scores to identify gene flow among baobab species; (2) the tree-based method QuIBL (quantifying introgression by means of branch lengths) designed to distinguish introgression from ILS^17^; and (3) PhyloNet^19^ to recover putative reticulate evolution.

**Fig. 2:**
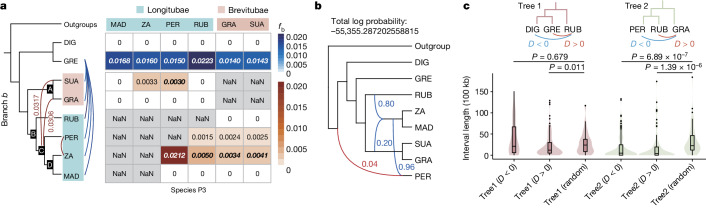
Test for introgression. **a**, *D*-statistics calculated between *Adansonia* species branches or internal tree branches (branch *b*, ordered A–D) and baobabs species (P3; acronyms as in Fig. 1a). The associated table is in two parts, the upper part (*Bombax ceiba* as the outgroup; *A. rubrostipa* as the reference genome) and lower part (*A. gregorii* as the outgroup and the reference genome) have cells coloured according to their *f*-branch score (*f*_b_) and show gene flow between each species tree branch and the species P3. C—*A. suarezensis* and C—*A. grandidieri* had high-value *f*_b_ (‘0’ indicates a zero score; italics depicts *Z*-score greater than 3; ‘NaN’ indicates an unperformable test). **b**, The best-supported network derived from analyses of 999 concatenated SCN gene trees in PhyloNet in runs with a 50% bootstrap threshold. Reticulation edges are coloured blue and the minor inheritance in *A. perrieri* is highlighted in red. **c**, Kernel density plots of the interval length between adjacent windows of introgressed genomic blocks (100 kb). The type of introgression is shown in **c** and for each tree topology, 100 windows were sampled randomly as a control. One-sided Wilcoxon test; left to right, *n* = 43, 109, 63, 136, 71, 62. In box plots, the centre line is the median, the box limits show the interquartile range (25th to 75th percentile), whiskers extend to quartiles ±1.5× interquartile range, and dots show potential outliers.

**Extended Data Fig. 2:**
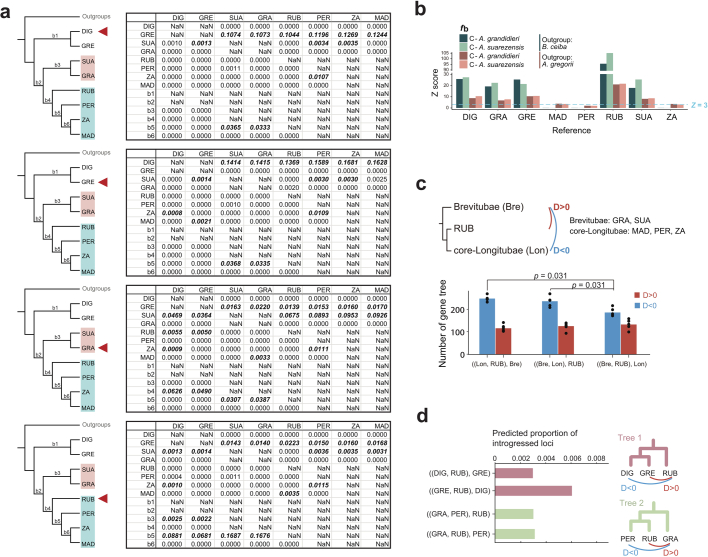
Test for introgression using different reference genomes. **a**, The inferred *D*-statistics were calculated and *f*-branch scores are shown between the species branches or internal tree branches (branch *b*, ordered b1-6) and baobab species (P3, horizontal row) using *Bombax ceiba* as the outgroup. The reference genome in each case is indicated by a red arrow. ‘0’ indicates a zero score returned in the analysis while significant cells with a *Z*-score > 3 are highlighted in italics. ‘NaN’ indicates an unperformable test. **b**, The *Z*-score statistics of *f*_b_ using different reference assembly genomes and outgroups (*Z*-scores > 3 indicate *f*-branch scores that were significantly larger than zero). **c**, Characterization of different SCN gene topologies from the genomic regions showing introgression signals. Two-sided Wilcoxon-test for paired samples (*n* = 6). **d**, The predicted proportion of introgressed loci that generate the triplet species topologies.

Patterson’s *D*-statistics detected an excess of shared derived alleles between *A. gregorii* and each of the Malagasy species, providing strong evidence of introgression (Fig. 2a and Extended Data Fig. 2a). To account for potential bias caused by mapping resequenced data to a reference genome using *D*-statistics^20^, we replicated the comparisons by using each baobab species as the reference. Similar results were found with all reference genomes, except when *A. gregorii* was the reference (Extended Data Fig. 2a, Supplementary Fig. 10 and Supplementary Table 4). For the Malagasy species, the frequent occurrence of shared alleles between non-sister lineages is probably a result of interspecific hybridization (Extended Data Fig. 2a and Supplementary Table 4). For example, analysis of *f*-branch score *f*_b_ values indicates extensive gene flow between *Adansonia* *za* and *A. perrieri* (high *f*_b_ value of 0.0212; Fig. 2a), a result that is supported by admixed microsatellite genotypes in individuals of *A. perrieri* and *A. za* occurring in sympatry and morphologically intermediate individuals^21^. It suggests that hybridization is continuing because of the overlapping distributions and flowering times of the two species^21^. Similarly, gene flow is predicted between the lineages leading to *A. za* and *A. rubrostipa* (*f*_b_ = 0.0050). Importantly, *f*_b_ values indicate gene flow between the ancestors of each Brevitubae species and the most recent common ancestor (MRCA) of Longitubae, excluding *A. rubrostipa* (Fig. 2a and Extended Data Fig. 2b). Such gene flow could have occurred before a shift in flowering phenology of the two sections (Brevitubae flower in the dry season and Longitubae in the rainy season^8^). As further evidence of introgression, we compared the topology of SCN gene trees in genomic regions with different introgression signals (*D* > 0 and *D* < 0). The result showed that genes in introgressed regions involving branches leading to Brevitubae and core Longitubae (*D* < 0) contributed significantly to the variable phylogenetic placement of *A. rubrostipa* (Extended Data Fig. 2c), an interpretation that is contrary to past assertions of introgression from *A. digitata* to Brevitubae^3^.

Evidence of introgression is supported by QuIBL, which shows that a tree topology ((*A. gregorii*, Malagasy species that is *A. rubrostipa*), *A. digitata*) has a lower Bayesian information criterion (BIC) value for an ‘introgression + ILS’ model than an ‘ILS-only’ model (Supplementary Table 5), even though less than 0.6% of loci were considered as introgressed (Extended Data Fig. 2d). This low estimation of introgression has probably been obscured by ILS among the three geographic lineages which have a short internal branch length in the phylogenetic tree (Fig. 1b) and similar proportions of variant tree topologies (that is, ((*A. digitata*, *A. gregorii*), Malagasy lineage), ((*A. digitata*, Malagasy lineage), *A. gregorii*), ((*A. gregorii*, Malagasy lineage), *A. digitata*)) (Fig. 1f). Given a sister relationship between *A. digitata* and *A. gregorii* (Fig. 1b) and gene flow between *A. gregorii* and the Malagasy baobabs (Fig. 2a), we suggest that *A. gregorii* was once in sympatry with the Malagasy baobabs, meaning that gene flow occurred after the split separating the Malagasy clade from the other two species. In addition, both *D*-statistics and QuIBL analysis showed signatures of gene flow between *A. digitata* and Malagasy baobabs, suggesting that *A. digitata* or the common ancestors of *A. digitata* and *A. gregorii* may have been in sympatry with the Malagasy baobabs (Extended Data Fig. 2a,d and Supplementary Table 5).

**Extended Data Fig. 3:**
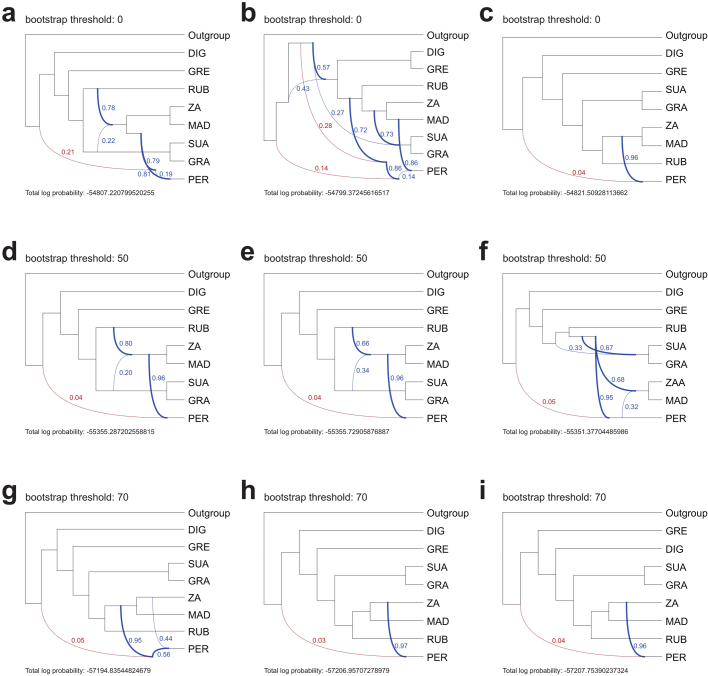
The reticulation history of baobabs inferred from PhyloNet. The best-supported networks (from left to right) were derived from analyses of 999 concatenated SCN gene trees using PhyloNet in three replicated runs with either (**a-c**) no bootstrap threshold, or with 50 (**d-f**) or 70 (**g-i**) bootstrap thresholds. Reticulation edges are coloured in blue, except for the reticulation edge involving *A. perrieri* which is in red. The inheritance probabilities are indicated next to the reticulation edges.

Further support for introgression was seen by analysing the dataset of 999 SCN genes using PhyloNet, implementing six search networks and allowing for zero to five reticulation events. The best-supported species networks showed that Longitubae, including *A. rubrostipa*, form a clade with a major inheritance probability (80%), whereas the placement of *A. rubrostipa* as sister to the entire Malagasy clade occurs with a minor inheritance probability (20%) (Fig. 2b). Given a consensus of monophyly of Longitubae in the *Adansonia* phylogeny, it is most likely that the 20% of loci are derived by introgression. The phylogenetic network is best interpreted as the product of historical hybridization between core Longitubae and the MRCA of Brevitubae, a result that is compatible with the *D*-statistics analyses (Fig. 2a). This reticulation event also explains the lack of topological consensus for the placement of *A. rubrostipa* in gene trees (Fig. 1c and Supplementary Figs. 7 and 8). The closer placement of *A. gregorii* to the Malagasy baobabs than between those species and *A. digitata* may also have resulted from introgression between the Malagasy baobabs and *A. gregorii*. Notably, PhyloNet species networks consistently identified about 4% admixture from a distantly related lineage into *A. perrieri* only. Although values of less than 5% admixture should be treated with caution, the stability of the signal suggests that it may be a biological feature reflecting the residual retention of ancestral genomic characters, possibly as a result of an extremely small population size^22^ (Fig. 2b and Extended Data Fig. 3). Small population sizes can fix introgressed segments rapidly by genetic drift, although of course they can also be lost rapidly, which might explain why this signal is not observed in other baobab species. Nevertheless, the possibility of the edge in *A. perrieri* as an artefact cannot be ruled out, as perhaps suggested by the lack of an *A. gregorii* + *A. digitata* clade on the species tree network.

The timing of proposed introgression events can be inferred by analysing the consecutiveness of introgressed regions in the genome, given that recent reticulation events are expected to result in longer stretches of introgressed segments than are more ancient events^23^. We investigated the length of the interval between two adjacent genomic windows of 100 kb in length that carry a significant signal of introgression (that is, *D* values deviated significantly from zero, *D* > 0 and *D* < 0). We compared these interval lengths against those returned from 100 random windows of 100 kb (Fig. 2c and Supplementary Fig. 11). Many windows with significant *D* values were found in the Malagasy species with interval lengths of 0 bp, indicating large and recent introgressed regions. By contrast, for the three geographic lineages, only *A. gregorii*-Malagasy species genomic windows had significant *D* values (*D* > 0), showing signatures of consecutive introgressed regions (Fig. 2c), which indicates earlier introgressions than those that occurred in Malagasy baobabs. As for gene flow involving *A. digitata*, the distribution pattern of introgressed windows suggests more ancient introgression, which could have occurred before polyploidy, followed by considerable recombination, breaking up introgressed segments as the polyploid genome diverged^24^.

## Genetic diversity and population dynamics

**Fig. 3:**
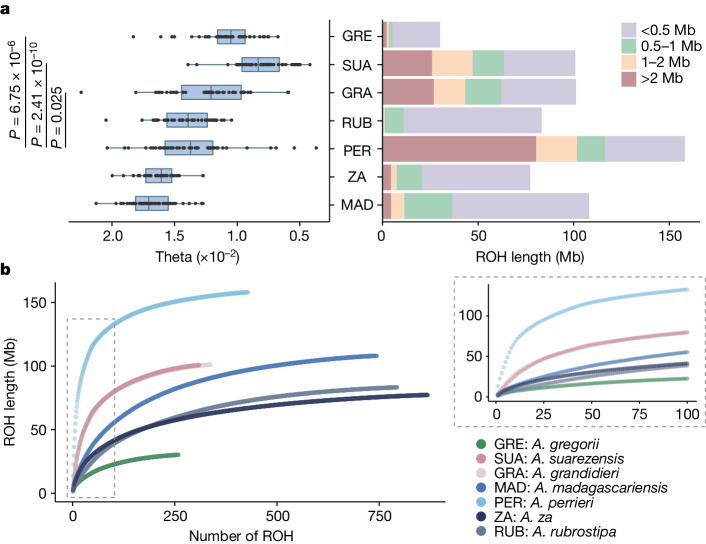
The genomic footprints of inbreeding and hybridization in diploid baobabs. **a**, GWH (left) and ROH (right) in seven baobab species (*A. digitata* is excluded because it is an autopolyploid). The heterozygosity statistics for all chromosomes of each species are presented as a box plot. The two-sided Wilcoxon test showed significantly higher levels of GWH in *A. perrieri* (*n* = 42) compared to species with, on average, longer ROHs (*n* = 44). In box plots, the centre line is the median, the box limits show the interquartile range (25th to 75th percentile) and whiskers extend to quartiles ±1.5× interquartile range. **b**, The relationship between the number and lengths of ROH. The dashed box shows the distribution pattern of the top 100 longest ROH in each species.

**Extended Data Fig. 4:**
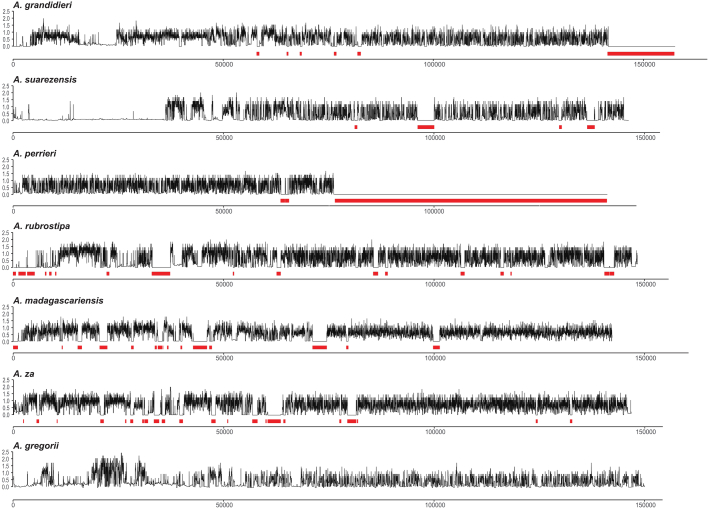
Visualized runs of homozygosity (ROH) along orthologous chromosome 15 in each baobab species. The y-axis shows the time to the most recent common ancestor (TMRCA) and the x-axis shows positions along the chromosome. Red bars below the black signal indicate regions that are inferred by the pairwise sequentially Markovian coalescent (PSMC) model to have coalesced recently and be ROH.

To investigate the genetic diversity of baobabs, we characterized genome-wide heterozygosity (GWH) and runs of homozygosity (ROH) in baobab genomes (*A. digitata* was excluded as it is autopolyploid) (Fig. 3a and Supplementary Tables 6 and 7). Both of the two endangered species, *A. suarezensis* and *A. grandidieri*, had lower GWH and numerous segments of long ROH (more than 2 Mb), which suggest high levels of recent inbreeding^25,26^ (Fig. 3a and Extended Data Fig. 4). Unexpectedly, the genome of the critically endangered *A. perrieri* exhibited high GWH (1.33%) despite the presence of abundant long ROHs (Fig. 3a). Compared to *A. suarezensis* and *A. grandidieri*, the higher GWH of *A. perrieri* could have arisen from gene flow with other Longitubae species (that is, *A. za*) (Fig. 2a). The abundant long ROHs may also have arisen by inbreeding between a few individuals in a constrained geographical range (Fig. 1a). *Adansonia madagascariensis*, *A. rubrostipa* and *A. za* had more short ROHs than other species, which may represent genomic remnants of ancient inbreeding combined with recent outcrossing (Fig. 3b). In contrast to the Malagasy species, the Australian species *A. gregorii* on average had shorter and fewer ROH segments and relatively low GWH (1.04%) (Fig. 3a), which probably arose from the ‘bottleneck’ caused by its dispersal to Australia^27^. The small number of ROH in *A. gregorii* could then be interpreted as a genetic consequence of its rapid radiation and high intraspecific gene flow^7^ in Australia.

**Fig. 4:**
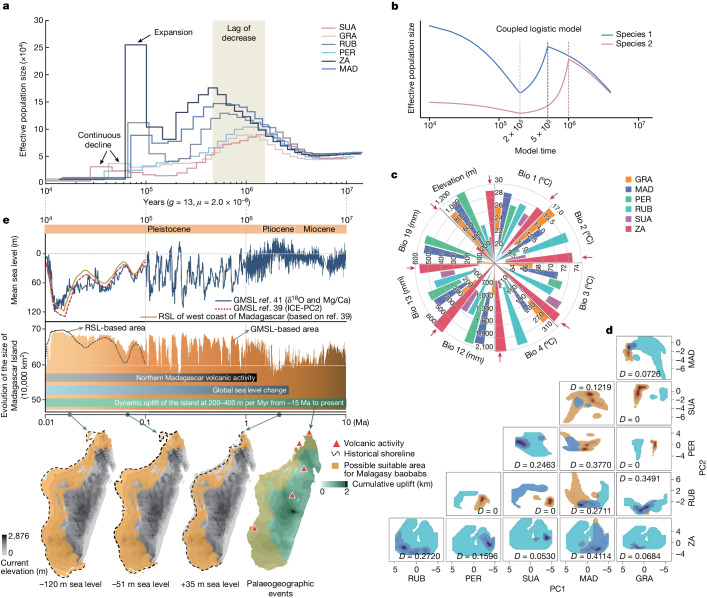
The ecological drivers of population dynamics in Malagasy baobabs. **a**, A PSMC model inferring the effective population sizes of Malagasy baobabs. A generation time of 13 years was used to translate substitution rates to ages. **b**, A coupled logistic model supporting the asynchronous population size decreases from around 1 Ma apparent in the PSMC model (Methods). **c**, Variation in ecological valence of six Malagasy baobabs to different environmental factors. The red arrows highlight the widest ecological range observed for *A. za*. **d**, Niche equivalency and similarity statistical tests and patterns of niche overlaps between baobabs. Coloured patches represent ecological niche differentiation of species pairs (pale blue and brown with areas of highest density being most intense; *x* axis, PC1; *y* axis, PC2). Areas of niche overlap are shown in blue. **e**, Geological events impacting the distribution of Malagasy baobabs. Changing sea levels over time (top, GMSL, RSL)^39,48^ was inferred by different models (Methods). The dynamics of the land area of Madagascar (middle) were derived from a calculation using the GMSL (orange patch) and RSL (dash line). The timescale of reported geological events^31,48,49^ is indicated by coloured strips. The predicted suitable areas of habitat for baobabs were mapped onto the simulated territories of the island under different sea-level scenarios (bottom). The acronyms used for species are as in Fig. 1a.

We estimated changes in effective population size (*N*_e_) through the Pleistocene using the pairwise sequentially Markovian coalescent (PSMC) model^28^. Analysis revealed an exponential increase in *N*_e_ for all Malagasy baobabs over the period 10–1 Ma (Fig. 4a). Then, from about 1 Ma, two distinct population dynamics emerged, with *A. grandidieri, A. suarezensis* and *A. perrieri* showing a decrease in *N*_e_, whereas the other Malagasy species (*A. za*, *A. madagascariensis* and *A. rubrostipa*) exhibited a lag phase before a decrease in *N*_e_ lasting about 0.3 Myr and then a significant recovery in *N*_e_ around 0.1 Ma (Fig. 4a). Inferences from coupled logistic models for species experiencing ecological competition^29^ are consistent with these data when models are perturbed by a dramatic and asynchronous population size decrease (Methods; Fig. 4b). It is likely that geological and environmental events in Madagascar (for example, uplift of mountains, mantle upwelling and volcanism)^10,30,31^, along with severe climate oscillations in East Africa around 1 Ma (ref. ^32^) may have contributed to these population dynamics. Furthermore, estimated inverse coalescence rates are a reflection of not only the census population size but also historical gene flow and population structures^33^. For instance, the significant increase of *N*_e_ in *A. za* at about 0.1 Ma could have arisen from population growth and extensive gene flow with other Malagasy baobabs, whereas the small *N*_e_ of *A. perrieri* over the last approximately 1 Ma may be due to high levels of inbreeding caused by low numbers of individuals and/or population fragmentation.

## Influences of geology and the environment

To understand the demography of Malagasy taxa and to infer past population dynamics, integrated ecological analyses were performed using present distributions (Supplementary Table 8) and 19 present- and palaeo-bioclimatic variables and altitude data from the WorldClim database v.2.0 (www.worldclim.org)^34^ (Supplementary Table 9).

**Extended Data Fig. 5:**
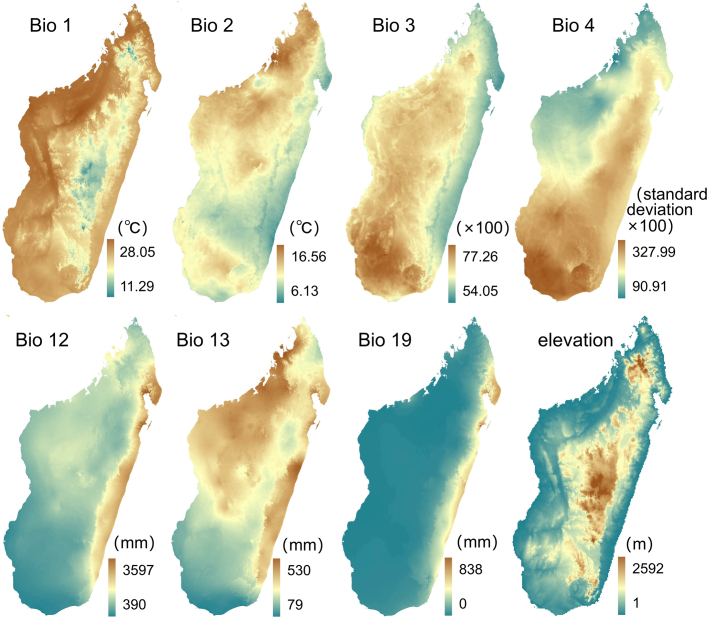
Patterns of spatial heterogeneity of environmental factors in Madagascar. Eight of 19 bioclimatic variables were filtered using Pearson correlation analysis (Pearson *r* < 0.8). The differences in climates were visualized in ArcGIS (version 10.2). Bio1 = Annual Mean Temperature (°C), Bio2 = Mean Diurnal Range (mean of monthly temperature ranges (monthly max temp – monthly min temp) (°C), Bio3 = Isothermality (Bio2 / Bio7) (×100), Bio4 = Temperature Seasonality (standard deviation ×100), Bio12 = Annual Precipitation (mm), Bio13 = Precipitation of Wettest Month (mm), Bio19 = Precipitation of Coldest Quarter (mm).

**Extended Data Fig. 6:**
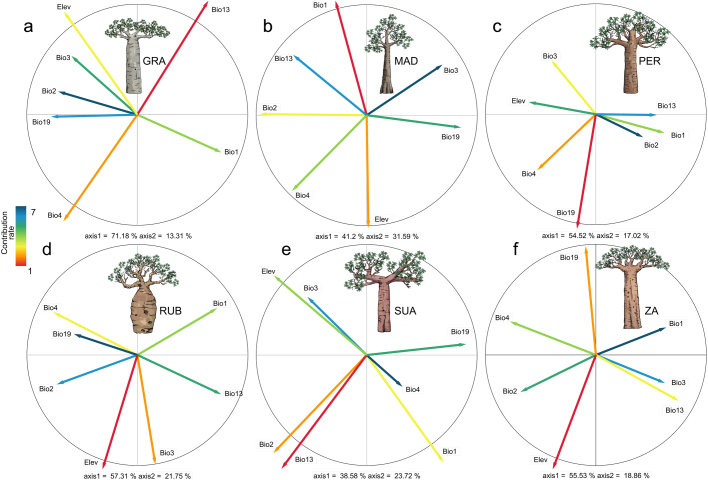
Principal component analysis of the main factors influencing the distribution of baobabs. For each species (species acronyms are given in Fig. 1a), the figures (**a**-**f**) show in the first two principal components (PC1 vs PC2) the relative contribution (line colour) of the bioclim variables. Bio1 = Annual Mean Temperature (°C); Bio2 = Mean Diurnal Range (mean of monthly temperature ranges (monthly max temp – monthly min temp) (°C); Bio3 = Isothermality (Bio2/Bio7) (×100); Bio4 = Temperature Seasonality (standard deviation ×100); Bio5 = Max Temperature of Warmest Month (°C); Bio6 = Min Temperature of Coldest Month (°C); Bio7 = Temperature Annual Range (BIO5-BIO6) (°C); Bio 8 = Mean Temperature of Wettest Quarter (°C); Bio9 = Mean Temperature of Driest Quarter (°C); Bio10 = Mean Temperature of Warmest Quarter (°C); Bio11 = Mean Temperature of Coldest Quarter (°C); Bio12 = Annual Precipitation (mm); Bio13 = Precipitation of Wettest Month (mm); Bio14 = Precipitation of Driest Month (mm); Bio15 = Precipitation Seasonality (Coefficient of Variation) (mm); Bio16 = Precipitation of Wettest Quarter (mm); Bio17 = Precipitation of Driest Quarter (mm); Bio18 = Precipitation of Warmest Quarter (mm); Bio19 = Precipitation of Coldest Quarter (mm). The results demonstrated that the spatial heterogeneity of altitude, temperature and precipitation act as the main environmental factors influencing the current distribution of Malagasy species, with *A. madagascariensis* and *A. rubrostipa* mainly influenced by temperature and altitude, while the remaining species were mainly affected by precipitation and altitude.

Ecological valence analysis revealed that *A. za* can tolerate the widest range of environmental conditions, encompassing many of the ecological niches occupied by the other species (Fig. 4c and Supplementary Table 10). Potentially *A. za* is strongly competitive for habitat space^35^ and able to replace more locally distributed species when in sympatry (for example, *A. suarezensis*, *A. grandidieri* and *A. perrieri*). Niche equivalency and similarity statistical tests also demonstrated that the ecological niche of all Longitubae species overlapped, with *A. za* having the highest coefficient of overlap (Schoener’s *D* = 0.411419) (Fig. 4d). The two endangered species, *A. suarezensis* and *A. grandidieri*, despite their close phylogenetic distance, have very different ecological niches, which suggests local adaptation after geographic isolation^36^ (Fig. 4d). Principal component analysis of environmental drivers of ecological niche differentiation indicated that the spatial heterogeneity of temperature, altitude and precipitation in Madagascar (Extended Data Fig. 5) could be the principal environmental factors leading to the present distribution of baobabs, with *A. madagascariensis* and *A. rubrostipa* mainly influenced by temperature and altitude, whereas the remaining species were mainly affected by precipitation and altitude (Extended Data Fig. 6).

**Extended Data Fig. 7:**
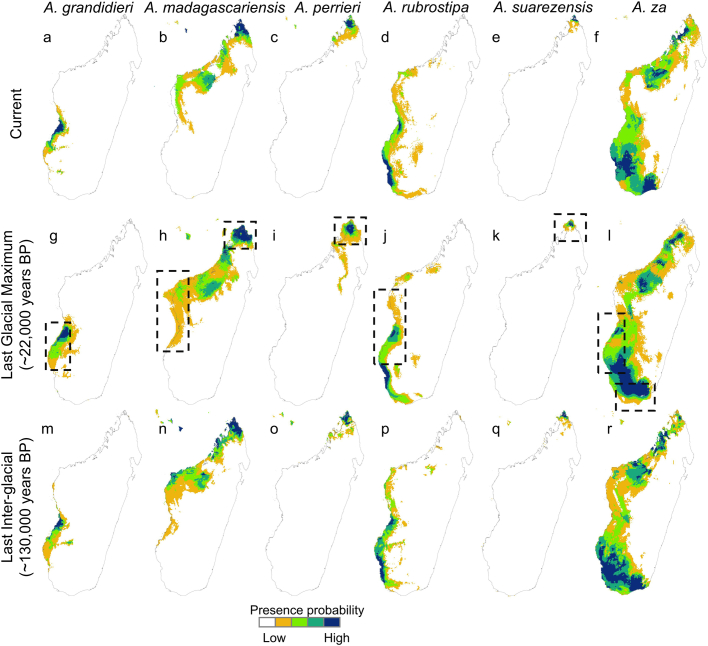
The potential distribution of six Malagasy baobab species in two past epochs modeled using MaxEnt software based on their current occurrence. Coloured patches reveal predicted current (a-f) and past (g-r) occurrences of suitable habitats for each species. The dashed boxes in g-l show probable range expansions of all species about 22,000 years ago, during the last glacial maximum (LGM). During that period, *A. grandidieri*, *A. madagascariensis* and *A. rubrostipa* probably had more extensive suitable habitat areas on the western coast of Madagascar, a habitat that has now largely disappeared (g, h, j). It is also likely that *A. suarezensis*, *A. perrieri* and *A. madagascariensis* had more suitable habitats in the north of Madagascar during the LGM period (h, j, k). In contrast, *A. za* is likely to have had more suitable habitats on the south of the island at that time (l).

**Extended Data Fig. 8:**
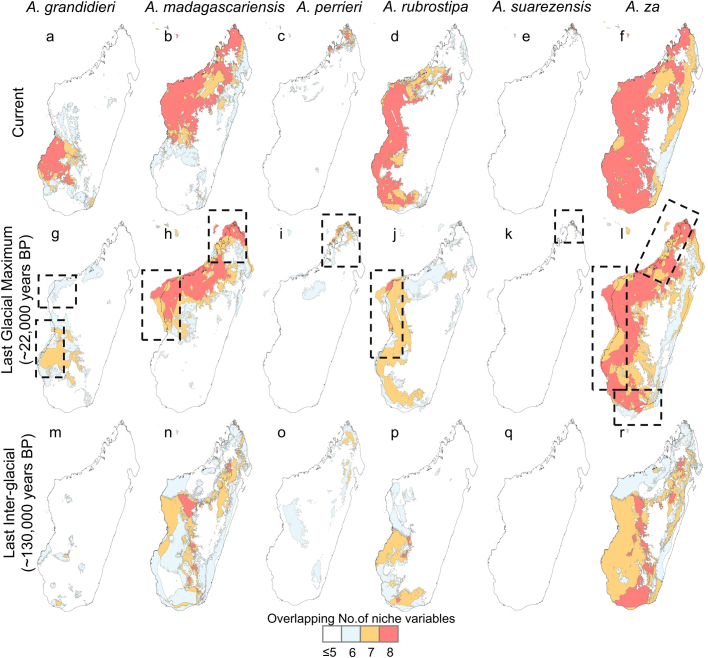
Potential suitable habitats for Malagasy baobab species and their possible recent dispersal trends. Predicted current and past distributions of suitable habitats for six species, derived from the spatial overlay of ecological amplitude layers of each species under each environmental factor (niche variables, spatial superimposition modeling). A higher number of parameter overlaps represents a higher probability of suitable habitat. The results show similar patterns to the paleo-distributions shown in Extended Data Fig. 7, except that this approach predicts more current suitable habitats for *A. za* on the northwest side of the island.

The varied environmental drivers of ecological niche differentiation in Malagasy baobabs, encouraged us to project their historical distribution using species distribution modelling (MaxEnt)^37^ (Extended Data Fig. 7) and spatial superimposition modelling (Extended Data Fig. 8). Both models indicated that almost all species previously had considerably larger ranges on the western coast of the island during the last glacial maximum (LGM; about 22,000 years before present; Extended Data Figs. 7 and 8), when global sea levels were low^38^. The results also imply that *A. suarezensis* and *A. perrieri* had more suitable habitats in the north of Madagascar at that time, suggesting that their distribution may have been continuous rather than fragmented as it is today.

We examined the likely effects of global mean sea level (GMSL)^38^ changes over the last 10 Myr on the distribution of Malagasy baobabs (Fig. 4e). The results indicated that about 4 Ma the island was approximately 11% smaller than today and 19% larger around 0.02 Ma (Supplementary Table 11). Given that past local sea levels were also influenced by the Earth’s viscoelastic structure and global ice sheet history^39^, the relative sea level (RSL) changes along the west coast of Madagascar from 0.12 Ma to the present were predicted, adopting from ref. ^40^ (Supplementary Fig. 12). The results were generally consistent with the predictions from GMSL changes (Fig. 4e). The present area of the island is likely to be relatively small, having declined from its largest extent during the LGM (when RSL reached about −120 m; Fig. 4e). During times of relatively low sea level, vast areas of Madagascar were probably suitable for baobab population expansion and dispersal, whereas periods of high sea level led to smaller suitable areas, population fragmentation, species isolation and reduced gene flow. From 10 Ma to present, mountain uplift (total 1–2 km at 200–400 m per Myr from about 15 Ma to present) and volcanic activity in the north of Madagascar^30,41^ (Fig. 4e) have also occurred^10,42^ and this also probably influenced areas suitable for baobabs (Fig. 4e). Changes in sea level and palaeogeographic events probably explain the limited distributions of *A. suarezensis* and *A. perrieri* in the north today, arising from a probable wider population range in the past (Fig. 4e). Those species with wider ecological valence (that is, *A. za* and *A. madagascariensis*) may have had more opportunities for population expansion, generating a dynamic that could have contributed to population declines of other baobab species. These interfered population dynamics are corroborated by the genomic signatures observed in the PSMC model. For instance, a remarkable decrease in population size probably occurred in *A. suarezensis* and *A. perrieri* over the last 1 Myr, a time when the northern area was impacted by sea level rises and volcanic activity (Fig. 4e).

## The continental origin of baobabs

**Extended Data Fig. 9:**
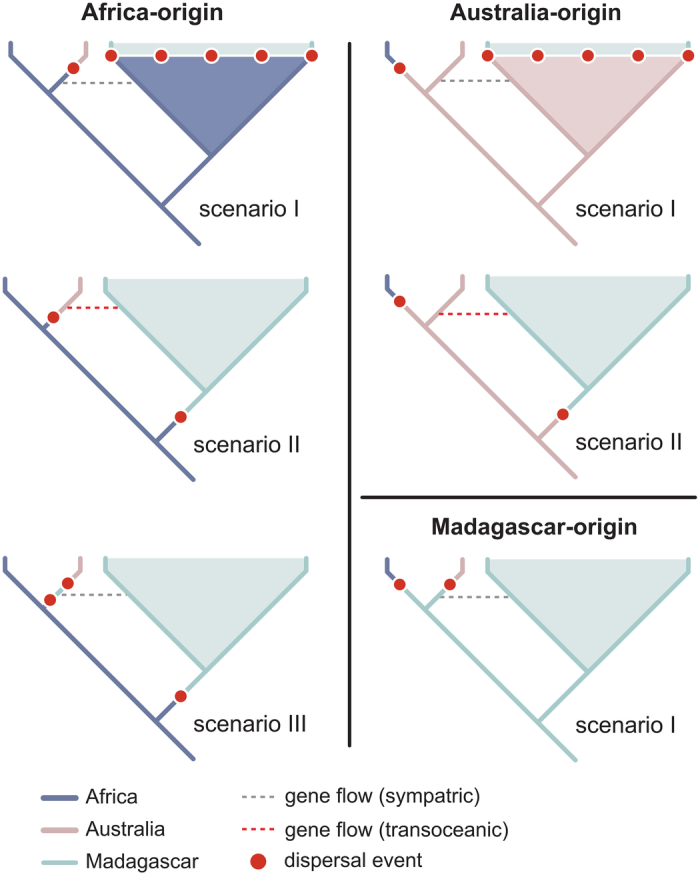
Different scenarios explaining three hypotheses of geographic origin for baobabs. The branch colors denote the geographic lineages of the species under each scenario with the occurrence of predicted, independent dispersal events (red dots, multiple dispersals are represented by five red dots, regardless of the exact number of times).

In the absence of fossils, it has been difficult to determine the ancestral location of origin for the stem group of *Adansonia* (Supplementary Note 2). However, the results presented here help dissect three hypotheses about the centre of origin for the crown group of baobabs (Extended Data Fig. 9). Critically, evidence of introgression between species indicates that *A. gregorii* is likely to have been in sympatry with the substantially diverged Malagasy baobabs during the diversification of the genus. Assuming so, there are three scenarios which explain the data from the perspective of an out-of-Africa hypothesis. One scenario is that the Malagasy baobabs must have colonized Madagascar by means of several independent dispersal events before going extinct in Africa. This assumes that the diversification of the Malagasy lineage occurred on the continent of Africa before these independent dispersals, which seems improbable. In a second scenario, if an ancestor of the Malagasy baobabs colonized Madagascar and diversified there (requiring only one dispersal event from Africa), then it necessitates transoceanic gene flow between the Australia-residing *A. gregorii* and each of the species in Madagascar independently. This also seems improbable because there are no validated instances for such transoceanic gene flow. In the third out-of-Africa scenario, there could have been two transoceanic dispersals from Africa to Madagascar, one being the ancestor of the Malagasy baobabs which diversified to form the Malagasy baobabs and another being the *A. gregorii* lineage which hybridized with the Malagasy baobabs before going extinct in Madagascar, requiring also an extra transoceanic dispersal of *A. gregorii* to Australia. Such a complex migration and extinction pattern for *A. gregorii* is not as parsimonious as other hypotheses.

With respect to the ‘Australia-origin’ hypothesis, as with the ‘Africa-origin’ hypothesis above, it would require either several independent dispersals or high levels of transoceanic gene flow. Beyond that, the out-of-Australia hypothesis is challenged by a probable bottleneck in the ancestry of *A. gregorii*, which is an expected product of long-distance dispersal.

Compared with the above two hypotheses, the ‘Madagascar-origin’ hypothesis offers the most reasonable explanation of the present data. First, the Madagascar-origin hypothesis requires only one dispersal event for *A. gregorii* from Madagascar to Australia, alleviating the requirements of unlikely transoceanic gene flow or several migrations. Second, only the Madagascar-origin allows for the assumption that geographical isolation between the Malagasy lineage and the other two baobab species started after the diversification of the Malagasy baobabs, enabling gene flow among all baobabs. This scenario is compatible with the gene flow detected between Malagasy baobabs and *A. gregorii* or *A. digitata*. Moreover, the hypothesis also explains the unexpectedly high proportion of gene trees (81.2% in gene-based trees and 68.1% in syntenic-guided trees) supporting the admixture of the Malagasy lineage and the other two lineages. Assuming a Madagascar-origin, the minor yet stable inheritance of ancestral *Adansonia* genes in *A. perrieri* was derived from long-term sympatry of *Adansonia* on the island. A stronger inference for a Madagascar-origin will be possible if/when relevant fossils and other robust historical information become available.

**Fig. 5:**
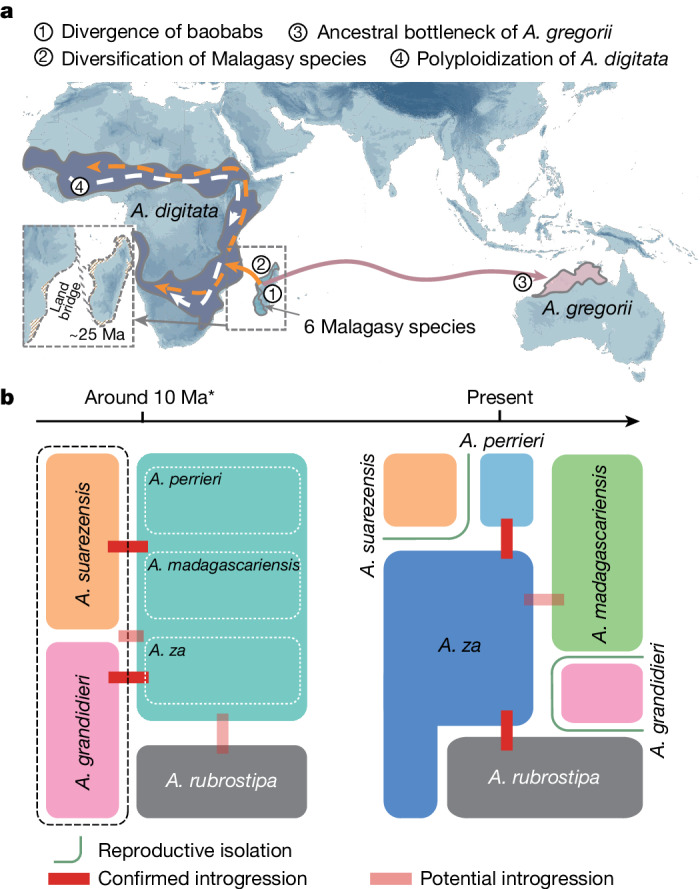
The evolutionary history of baobab trees. **a**, A summary of the predicted evolutionary history of baobabs under the Madagascar-origin hypothesis showing the probable migration routes of *A. digitata* and *A. gregorii*. The location of a controversial ancient land bridge^49,50^ between Africa and Madagascar is shown (dotted box) along with key evolutionary events. **b**, A summary of the diversification of Malagasy baobab species, including dynamic changes in population sizes, recurrent introgression and geographic isolation from 10 Ma to present. The size of each species block around 10 Ma corresponds to the estimated population size based on a PSMC model and predicted ecological inferences, and the thick red lines show proposed gene flow based on *f*-branch analysis (translucent red lines indicate proposed introgression that is undetectable using present methods). *Indicates the time approximation given by the PSMC model.

Building on the Madagascar-origin hypothesis, it is possible that the autopolyploid *A. digitata* arose in Madagascar, then dispersed to east Africa where it spread across the entire continent. However, that scenario is apparently compromised by the occurrence of most ancestral plastid haplotypes of *A. digitata* in West Africa^43^. Such haplotypes are indicative of an early autopolyploid colonizer being of West African origin, before it later spread eastwards and southwards across much of savannah Africa. To reconcile the conflict, a diploid progenitor of *A. digitata* may have dispersed from Madagascar to Africa (Fig. 5a). Then, probably in west Africa, autopolyploidy occurred leading to the extant tetraploid cytotype which replaced the diploid progenitor. There are several examples showing that autopolyploids can differ in their ecological tolerances from their diploid progenitors, perhaps arising from polysomic inheritance, increased heterozygosity (heterosis) and altered gene expression, all of which can contribute to an increased competitive advantage and range shifts^44^. Potentially, *A digitata* benefited substantially from the neo-polyploidy event, which led to its present wide distribution across Africa (Fig. 5a).

Madagascar is renowned for its high rates of endemism^10^, including *Adansonia*. *Adansonia’*s phenology shifts coupled with geographic isolation and interspecific hybridization have collectively contributed to the diversity of Malagasy baobabs. For the two phylogenetic sections of Malagasy baobabs (Brevitubae and Longitubae), we predict that the fixation of diverged flowering time was a critical step in establishing reproductive barriers (more discussion on baobab pollination ecology is provided in Supplementary Note 3). We also predict that global changes in sea level and local palaeogeographic events, resulting in population isolation and expansion, were important in shaping their population dynamics and facilitated local adaptation. Hybridization between Brevitubae and core Longitubae is also likely to have contributed to the diversification of Malagasy baobabs^45^ (Fig. 5b). These processes are continuing, as indicated by reports of recent hybridization between *A. rubrostipa* and *A. za*^21^. Although diversification of *Adansonia* in Madagascar seems most likely, there is potentially an unresolved story relating to the dispersal of the lineage leading to *Adansonia*, given that the present distribution of the closest relatives to *Adansonia* are South America^46^.

## The conservation status of baobabs

The genomic and ecological analyses presented here enable a re-evaluation of the conservation status of baobabs. According to the IUCN Red List criteria, the two Brevitubae species are now listed as endangered (with *A. suarezensis* coded B1ab+2ab and *A. grandidieri* A4c, IUCN Red List, 2023). Our predictions, based on PSMC analyses and the identification of potentially suitable habitats, show considerable population size reductions for these species from 1 to 0.1 Ma and from the LGM (about 22 thousand years ago (ka)) to the present (Supplementary Fig. 13). Despite these reductions being less than 80% over this period (that is, below the threshold for assessment as critically endangered), the large amounts of long ROH in *A. suarezensis* and *A. grandidieri* and the small *N*_e_ (based on the PSMC analyses) are features that indicate constrained population sizes^26^. These features plus their distinct ecological niches, narrow ecological valence and low genetic diversity^22^ suggest that the two species are likely to have reduced resilience to ecological perturbations and habitat fragmentation^36^. In addition, coupled logistic models for species experiencing ecological competition fit the past population dynamics of species in Brevitubae and Longitubae (*A. perrieri* excluded) (Fig. 4b), whereas previous studies modelling the distribution of *A. suarezensis* and *A. grandidieri* forward in time to 2050 and 2080 (ref. ^47^) suggest that climate change will pose severe threats for *A. suarezensis*, leading to its extinction before 2080. We therefore propose the conservation status of *A. suarezensis* be moved from endangered to critically endangered on the basis of criterion A3ce of the IUCN Red List. Beyond that, only three of the six subpopulations of *A. suarezensis* are now included in the protected area network in Madagascar (IUCN Red List, 2023). We therefore recommend that conservation efforts should be focused on the three subpopulations outside the protected area network.

For *A. grandidieri*, given the interspecific competition indicated, we recommend that the IUCN rejustify its endangered status regarding criterion A4ce. More conservation measures and studies are required, especially the evaluation of its population dynamics with respect to other sympatric baobab species. In particular, its suitable habitat completely overlaps with *A. za* (Fig. 4d), a species predicted to have undergone large population expansions in the past (Fig. 4a) and characterized by the widest ecological valence amongst baobabs (Fig. 4c).

Despite *A. perrieri* (critically endangered) having relatively high GWH, mediated by introgression from other baobabs, especially from *A. za* (Fig. 2a), the numerous long ROHs suggest inbreeding and a very small *N*_e_. In addition, PSMC analysis shows that its *N*_e_ has been continuously decreasing for about a million years (Fig. 4a). It is likely that *A. za* has been genetically swamping *A. perrieri*, rather than enabling its genetic rescue from the disadvantages of a small *N*_e_. Moreover, hybridization between *A. perrieri* and *A. za* seems to be continuing, given the occurence of morphologically intermediate individuals^21^. Hence, we highlight the potentially negative genetic influence of *A. za* on *A. perrieri* and recommend that individuals in sympatry should be discouraged if species distinctiveness is desirable.

Further research into the population genetics of baobabs is needed, especially for *A. rubrostipa* and *A. madagascariensis*, which overlap with *A. za* in their distribution and flowering time. Similarly, the present genetic understanding of baobabs would benefit from broader sampling to bridge the gaps in the evolutionary history of baobabs. The key priority for safeguarding *Adansonia*, especially the two species in section Brevitubae and *A. perrieri*, should be focused on providing data to guide both in situ and ex situ conservation programmes, to enable these iconic species to recover and thrive.

## Methods

### Plant materials and whole-genome sequencing

Young leaves of *A. digitata* and *A. gregorii* were collected from trees in wild populations. The ripe fruits of Malagasy baobabs were collected from natural populations and seeds were further germinated for fresh young leaves at a local nursery garden in Antananarivo (Madagascar). Information on voucher specimens and the locality of the individuals sampled are provided in Supplementary Fig. 14. Genomic DNAs were extracted locally using the DNeasy Plant Mini kit (Qiagen) with a modified protocol. A washing step was included before CTAB buffer extraction to exclude the secondary metabolites in baobab leaves. The washing buffer contained 50 mM Tris-HCl, 5 mM EDTA-2Na, 0.35 M d-sorbitol, 1% (w/v) polyvinylpyrrolidone (PVP-K30) and 1% beta-mercaptoethanol. The RNA was isolated using TRIzol (Invitrogen) and further treated with RNase-free DNase I (Promega).

Next-generation sequencing (NGS) sequence datasets were generated (DNBSEQ short reads, PacBio long reads and Hi-C short reads) for genome assemblies. The fragmented reads (2 × 150 bp) with insert sizes of 300–500 bp for paired-end reads were produced using the DNBSEQ platforms. For the PacBio long reads, the high-molecular-weight gDNA with an insert size of approximately 20 kb was collected using BluePippin (Sage Science) and was sequenced on PacBio Sequel I/ Sequel II platform. The DNA libraries were further sequenced on PacBio Sequel I SMRT cells with continuous long reads or PacBio Sequel II SMRT cells in circular consensus sequence (CCS) mode. Fresh young leaves were fixed in a 2% formaldehyde solution for Hi-C library construction. The nuclei/ chromatin was digested with MboI (New England Biolabs) as previously described^51^. Finally, the Hi-C libraries were sequenced with DNBSEQ to produce 150 bp paired-end reads.

### *K-*mer analysis for genome size estimation

The short reads were first filtered by SOAPnuke (v.1.5.6) (-n 0.01 -l 15 -q 0.4 -i -Q 2 -G -M 2 -A 0.5 -d)^52^. The clean data were then used for *K-*mer analysis to estimate genome size using jellyfish (v.2.3.0)^53^ and Genomescope1.0 (ref. ^54^). The ploidy level of *A. digitata* was further analysed using Genomescope 2.0 and Smudgeplot (v.0.2.5)^55^.

### Genome assembly and contigs anchoring

Falcon v.0.5 (ref. ^56^), a hierarchical, haplotype-aware genome software, was used to generate the initial contigs. Meanwhile, the pa_HPCdaligner_option with parameters set to ‘-v -D24 -B8 -M32 -h300 -w8 -e.75 -l2000 -s1000 -k18’ and the ovlp_HPCdaligner_option with ‘-v -D251 -B24 -M36 -h600 -e.96 -l1000 -s1000 -w6 -k25 -T8’ were used for pre-assembly and read corrections. The initial contigs were further polished with all sequenced PacBio long reads using the Arrow algorithm (https://github.com/lixuenan/GenomicConsensus). Then, the polished genome sequences were error-corrected using the short insert size reads by Pilon (v.1.23)^57^ with default settings. The whole-genome assemblies were further processed with purge_haplotigs (https://bitbucket.org/mroachawri/purge_haplotigs/src/master/) to remove redundant sequences.

For the PacBio CCS HiFi data, the primary contigs were assembled using Hifiasm (v.0.15.1)^58^ with default parameters. To anchor the contigs to chromosomes, the Hi-C cleaned reads were aligned to the assembled genome. The global mapped reads were obtained using bwa (v.0.7.12). The Juicer pipeline (v.1.5)^59^ was applied to process the alignments of Hi-C reads. The 3d-dna (v. 180922)^60^ was used to link the contig assemblies into chromosome-length scaffolds guided by linking information. The Hi-C contact maps were processed with 500 kb using the visualized module of 3d-dna and reviewed in JuiceBox (v.1.9.0) (https://github.com/aidenlab/Juicebox).

### Genome annotation and assessment

Two strategies, including homology-based and de novo methods, were applied for repetitive sequence annotation. The homology-based method was performed using the database of repeat sequences in RepBase (v.21.12) (http://www.girinst.org/repbase)^61^. On the basis of Repbase, RepeatModeler (v.2.0)^62^ (http://www.repeatmasker.org/RepeatModeler/) and LTR_FINDER (v.1.07)^63^ (http://tlife.fudan.edu.cn/ltr_finder/) were also used to identify repetitive sequences in baobab genomes. For the de novo method, we first generated a repetitive sequence database using de novo prediction based on the features of sequence alignment and transposable element structure. Then, the repetitive sequences were refined and verified by REPEATMASKER (v.3.3.0; http://www.repeatmasker.org). In addition, tandem repeats were identified using Tandem Repeats Finder (v.4.09)^64^ (http://tandem.bu.edu/trf/trf.html).

Three different algorithms, transcriptome data-based prediction, homology-based prediction and ab initio prediction, were collectively applied for gene structure prediction and annotation. The following pipeline was used for each baobab species studied here. (1) RNA-seq reads from root, stem and leaf were, respectively, mapped to the assembled genome using hista2 (v.2.1.0)^65^. The transcripts were generated by stringtie (v.2.1.6)^66^ on the basis of the aligned results. (2) The protein sequences of *Arabidopsis thaliana*^67^, *Gossypium arboretum*^68^, *Durio zibethinus*^69^, *T. cacao*^70^ and *G. raimondii*^71^ were aligned to the baobab genomes using GeMoMa (v.1.6.4)^72^ with default parameters. On the basis of the results of homology prediction, the genes with complete integrity of structure were preserved to train the gene model analysis which was then used with AUGUSTUS v.3.2.3 (ref. ^73^) and SNAP (v.2006-07-28)^74^. (3) For de novo prediction, two gene prediction programmes, AUGUSTUS (v.3.2.1)^73^ and SNAP were used to predict coding regions in the repeat-masked genome. Finally, the information of repetitive sequences, transcripts and homology-based gene predictions were integrated into the Maker-P (v.2.31.11)^75^ with the following parameters ‘max_dna_len = 100000, split_hit = 25000, always_complete = 1, split_hit = 25000, est2genome = 0, protein2genome = 0’.

### Historical activity of LTR retrotransposons

The LTR retrotransposon candidates (LTR-RTs) were de novo detected in the genomes of the eight baobab species through LTR_Finder (v.1.0.7)^63^ and LTRharvest incorporated in Genome Tools (v.1.5.8)^76^ with default parameters. The two datasets were integrated to remove the false positives using the LTR_retriever package (v.1.9) with default parameters. To further classify LTR-RTs into detailed clades, the TEsorter (v.1.3.0)^77^ (https://github.com/zhangrengang/TEsorter) hidden Markov model profile-based classifier was used with default settings, taking as reference the protein domains found in the REXdb Viridiplantae version of the database^78^. Meanwhile, LTR_retriever (v.1.9)^79^ was also applied for estimating the insertion times of LTR-RTs. The rice mutation rate of 1.3 × 10^−8^ mutations per site per year was adopted in this study.

### Syntenic analysis and whole-genome duplication detection

The syntenic gene pairs between species were identified using JCVI (v.1.1.11, a Python version of MCscan, https://github.com/tanghaibao/jcvi/wiki/MCscan-(Python version)). By using the coding sequences (CDS) and annotation gff3 files as the input data, the syntenic blocks for each paired species were detected using ‘jcvi.compara.catalog ortholog’ with the parameter ‘–cscore = 0.7’. The syntenic blocks were filtered by ‘jcvi.compara.synteny screen’ with parameters ‘–minspan = 90 –simple’. The MCScanX-170908 (ref. ^80^) was used to construct chromosomal syntenic blocks at interspecific and intraspecific levels (parameters: -a -e 1e-5 -s 5). With the HKY substitution model^81^, the paralogous and orthologous gene pairs in syntenic blocks were used to calculate fourfold synonymous third-codon transversion rate (4dTv) distances to look for evidence of WGDs^82^. The *K*_s_ distribution was calculated using the Nei–Gojobori method implemented in yn00 programme of PAML (v.4.5)^83^.

### Orthologous gene set construction

A total of 15 species were used for orthogroup sorting, including the whole *Adansonia* genus (eight baobab species), four other Malvaceae species (*T. cacao*, *D. zibethinus*, *G. arboretum* and *G. raimondii*) and three outgroup species (*Populus trichocarpa*, *A. thaliana* and *Carica papaya*). The protein sequence references were collected from the available data sources (Supplementary Table 2). The longest transcripts were chosen for those genes with alternative splicing variants. The similarities between protein sequences were calculated using BLASTP (2.9.0+) with an e-value threshold of 1 × 10^−5^. Then, OrthoMCL (v.1.4)^84^ was used to identify gene families on the basis of the similarities of the genes. Markov chain clustering (MCL) was used with default parameter settings except with a main inflation value of 1.5.

### Phylogenetic inference and molecular dating

A total of 1,086 single-copy orthologous genes were obtained from the above orthogroup sorting and were further aligned by MUSCLE (v.3.8.31)^85^. The protein alignments were converted to corresponding CDS. Each amino acid was substituted for the corresponding triplet bases from its CDS according to the same ID information. These coding sequences for each single-copy gene family were concatenated to form one super gene for each species. The secondary nucleotides of each codon with phasing alignment were extracted and used for constructing the phylogenetic tree using MrBayes (v.3.1.2)^86^ under the GTR model. The divergence time between species was estimated using the MCMCTREE algorithm from the PAML package (v.4.5)^83^, using an autocorrelated clock model based on the inferred phylogenetic relationships. Two calibration times from the TimeTree database (http://www.timetree.org/) for (1) the divergence between *A. thaliana* and *C. papaya* (54–90 Ma, (2) *A. thaliana* and *P. trichocarpa* (100–120 Ma) and (3) the bombacoid–malvoid split^87^ (66.3–42.6 Ma) were used for the molecular dating. Coalescence gene trees were constructed with each gene applied using RAxML-8.2.11. Then coalescence gene trees were reconstructed using ASTRAL-5.6.1 (ref. ^88^) and each topology was calculated.

For the syntenic-based phylogenetic analysis, the eight baobab genomes were aligned to the *G. raimondii* ‘D5-4’ reference genome (https://www.cottongen.org/species/Gossypium raimondii/NSF-D5) using LASTZ 1.04 (https://github.com/lastz/lastz/) with default parameters. The pairwise alignments were systematically chained and integrated to generate high-quality blocks. Subsequently, the non-syntenic regions were excluded and pairwise local alignments were merged to generate a final multispecies whole-genome alignment using MULTIZ (v.11.2)^89^. Syntenic fragments of more than 3 kb were considered for further concatenation. The concatenated fragment represented by *G. raimondii* was subjected to a sliding window-based phylogenetic analysis with a window size of 20 kb. Each individual window was used to construct a phylogenetic tree using RAxML-8.2.11. The likelihoods of each potential topology were input into CONSEL (v.1.20)^90^ and an approximately unbiased test was conducted with a significance level of *P* > 0.95.

De novo assembly of the plastome sequence of each baobab species was performed by NOVOPlasty 4.3.1 (ref. ^91^). After alignment with MUSCLE, we used RAxML to infer maximum-likelihood trees using the GTR-Γ model with 1,000 bootstrap replicates. The *Bombax ceiba* plastome sequence was set as the outgroup.

### Variant calling and filtering

For each baobab species, the clean paired-end reads from NGS were mapped back onto its assembled genome with bwa (v.0.7.17)^92^ using default parameters. Samtools (v.1.9)^93^ was then used to sort the aligned reads and Picard (v.2.21.6)^94^ was used to remove redundant reads. Variant detection and filtering were carried out using GATK (v.4.1.2.0)^95^ with the filtering parameters set to ‘QD < 2.0, QUAL < 30.0, SOR > 3.0, FS > 60.0, MQ < 40.0, MQRankSum < −12.5, ReadPosRankSum < −8.0’. The output VCF file set was named as ‘align_SELF’. To avoid any probable conflicting results caused by choice of reference in downstream analyses, we aligned the cleaned NGS reads of each baobab species to the assembled genome of all other baobab genomes. After variant calling and filtering, all output files based on the same species’ genome reference formed a dataset and named as ‘align_REF’.

### Identification of copy number variations

To detect CNVs in all baobab species and the outgroup *B. ceiba*^96^, the sorted BAM file of align_REF was processed by CNVpytor (v.1.2.1)^97^ for genome alignment based on the genome of *A. gregorii*. The CNV calling was carried out by setting a bin size of 1,000 bp and the following criteria were set to filter false positive candidates: the CNV calls with *P* < 0.0001, sizes greater than 5 kb, a fraction of mapped reads with zero quality (*q*_0_) > 50% and the distance to the nearest gap in reference genome (*d*_G_) > 10,000. Duplication and deletion information was extracted from the CNV calls and then used to reconstruct the neighbour-joining tree with the R package ape (v.5.6-2).

### Gene flow

The Patterson’s *D* and *f*_b_ admixture ratio for all possible combinations of baobab species were calculated using Dtrios in Dsuite (v.0.5 r47)^98^ on the basis of the align_REF dataset. For the calculation of all baobab species, *B. ceiba* was chosen as the outgroup, whereas, for that of the Malagasy baobabs, *A. gregorii* was chosen as the outgroup. To further specify local introgressed genomic regions, Patterson’s *D* was calculated for the whole genome using ABBA-BABAwindows.py in genomics_general-master2 (v.0.4) with a sliding window of 100,000 bp. We defined the introgressed regions that were ranked in the top 1%, top 5% and top 10% of the absolute *D* value, respectively.

The tree-based method QuIBL (https://github.com/miriammiyagi/QuIBL)^17^ was used to differentiate introgression from ILS. Because only four lineages can be involved in a single run of QuIBL, we focused on two previous trees: (((*A. digitata*, *A. gregorii*), *A. rubrostipa*), *B. ceiba*) (*B. ceiba* as outgroup) and (((*A. perrieri*, *A. rubrostipa*), *A. grandidieri*), *A. gregorii*) (*A. gregorii* as outgroup). To carry out the QuIBL analysis, we used RAxML to construct the input files of QuIBL on the basis of the fasta alignments of the 999 single-copy orthologous genes. We distinguished between an ILS-only model and an introgression + ILS model using the BIC test with a strict cutoff of ΔBIC > 10.

To construct the species networks, we used the tree-based maximum-pseudolikelihood technique implemented in PhyloNet v.3.8.2 (refs. ^99,100^). The set of 999 SCN gene trees was used as the input, the same as that in ASTRAL. Twenty-seven parallel network searches were carried out by allowing zero to five reticulation events. The number of runs of the search (-x) was set as 50. To identify the potential influence of edges with low supporting rate, we ran it three times with no bootstrap threshold, with a threshold of 50 (-b 50) and with a threshold of 70 (-b 70). All other parameters remained as default settings. To estimate the best network among models with different numbers of reticulation events, we performed model selection using the Akaike information criterion and the BIC.

### Genome-wide heterozygosity estimation

We characterized the GWH for all baobab species in theta. The theta of each chromosome was calculated in MlRho (v.2.9)^101^ on the basis of the align_SELF dataset of each diploid species.

### Demographic inference and ROH estimation

We applied the pairwise sequentially Markovian coalescent (PSMC)^28^ method to explore the demographic history of all diploid baobab species. On the basis of the align_SELF dataset for each species, PSMC (v.0.6.5-r67) analyses were conducted with parameters ‘-N25 -t15 -r5 -p ‘4 + 25*2 + 4 + 6’’. A generation time (*g*) of 13 years and a substitution rate (*μ*) of 2 × 10^−8^ were used to estimate historical changes of effective population size over time. The consistency of the demographic predictions was tested by performing 100 bootstrap replicates. ROH segments were characterized by genome regions with a low best-fit *K* value (less than or equal to 2) following the method suggested in refs. ^25,102^.

### Coupled logistic modelling

We demonstrate a solution to a pair of coupled logistic equations to simulate population size changes of two species with ecological competition^29^ using the R package deSolve (v.1.35):

$$\begin{array}{c}{\rm{d}}X=a\times X\times (1-(X+Y)/m)\\ {\rm{d}}Y=b\times Y\times (1-(X+Y)/n)\end{array}$$

where *X* and *Y* are the population size of either species 1 or 2; *a* and *b* are the constant growth rate of population size of each species in the absence of interactions with other species; and *m* and *n* are the equilibrium population size of each species.

Three stages were divided in the simulation: at the first and third stages, two species were parameterized with the same settings to simulate population growth; a perturbation was simulated at the second stage in both species, with the perturbation to species 1 delayed for 5 × 10^5^ unit of model time.

### Species distribution data

The present distribution data (in both longitude and latitude) for the six Malagasy species were obtained from publically available sources, including the Global Biodiversity Information Facility (https://www.gbif.org/; 28 November 2022, 10.15468/dl.ajuz23), as well as our own field surveys made in 2018 (ref. ^103^). Repetitious and erroneous records were removed from the integrated dataset giving a total of 742 occurrence points (90 for *A. grandidieri*, 49 for *A. suarezensis*, 87 for *A. madagascariensis*, 41 for *A. perrieri*, 256 for *A. rubrostipa* and 219 for *A. za*) which were kept for the subsequential analyses.

### Environmental variables

We extracted the present- and palaeo-climate data for 19 bioclimatic variables from the WorldClim database v.2.0 (ref. ^34^). The last interglacial (LIG; about 120,000–140,000 years before present) and the LGM (about 21,000 years ago) were defined as the palaeo-climate in this study. The elevation data were also collected from the WorldClim database. All variables were downloaded at a spatial resolution of 2.5 min (about 5 km resolution at the equator). The LIG variables were downloaded at a resolution of 30 arcsec and resampled in ArcGIS 10.2 at a spatial resolution of 2.5 min. To circumvent the effect of highly correlated variables, a Pearson correlation analysis (Pearson *r* < 0.8)^104^ for all 19 bioclimatic variables was conducted and eight bioclimatic variables with the lowest correlations were selected for subsequent analysis, these being: annual mean temperature (Bio 1), mean diurnal range (Bio 2), isothermality (Bio 3), temperature seasonality (Bio 4), annual precipitation (Bio 12), precipitation of the wettest month (Bio 13) and precipitation of the coldest quarter (Bio 19). More details of the variables are given in Supplementary Table 9.

### Species distribution modelling

The MaxEnt 3.4.1 program (http://biodiversityinformatics.amnh.org)^37^ was used to model the present- and palaeo-distribution of the six Malagasy baobab species. Auto feature function was used for the modelling, 75% of the point occurrences were randomly selected for the model training and cross-validation. Meanwhile, 25% of the data was set aside for model testing and independent validation when the test sample was chosen at random. Cross-validation was used as the replicated run type and other parameters were set to default values. We computed the area under the curve in test data^105^ to validate the specificity and sensitivity of our model in this study.

### Palaeo-distribution prediction by ecological niche consistency

The ecological amplitude refers to the tolerance range of a plant to environmental factors, that is, the range between the highest and lowest values tolerated for each environmental factor documented. The ecological amplitude threshold of a species under each environmental factor was extracted through ArcGIS on the basis of its present distribution. Subsequently, the spatial pattern of the ecological amplitude threshold for each environment factor in Madagascar at different time periods was determined spatially by ArcGIS. Correspondingly, the eight environmental factors were superimposed on the layers and the areas with the most overlapping layers were considered to correspond to the potential habitat of a species.

### The analysis of niche overlap

The statistical tests for niche equivalency and similarity were computed from the density in environmental niche space^106^. The niche equivalency can assess whether the niches of two species being compared are equal (show constant overlap), moderately similar (show some overlap) or significantly different (show no overlaps) when occurrences are randomly shuffled across the ranges. We performed the niche equivalence test by comparing the niche overlap (Schoener’s *D*) values for sets of two species under the overlap of a null distribution. The null distribution was created by extracting the climatic variables from a randomly selected set of coordinates across the background region of the study area. Correspondingly, Schoener’s *D* varied from 0 (no overlap) to 1 (complete overlap). The comparative computing was replicated 1,000 times^107^. The niche similarity test was performed to assess the statistical significance of a measured niche difference against niches of the null model taken randomly in a given background area. These analyses were carried out in R software with the package ecospat (v.3.4)^108^.

### The geological history of Madagascar

The GMSL data are mainly from results obtained from the Pacific benthic foraminiferal δ^18^O and Mg/Ca records^41^. The RSL simulation we have adopted is taken from ref. ^40^. A total of 30 sample sites were randomly selected along the west coast of Madagascar for the assessment. The historical area of island and palaeo-coastline under GMSL and RSL were reconstructed by the Global Elevation Layer (https://www.gmrt.org/) using ArcGIS, respectively. The topography map of Madagascar at 5–15 Ma was referenced from ref. ^49^.

The simulation of potential distribution areas of baobabs (by MaxEnt) in the LIG (Extended Data Fig. 7) was applied when reconstructing the suitable areas under different sea levels. Considering that elevation is one of the main factors contributing to the distribution of almost all Malagasy baobabs (Extended Data Fig. 6), we adjusted the easternmost boundary of baobab distribution according to sea-level changes whereas the westernmost boundary coincided with sea level. The cumulative uplift history and palaeogeographic events are introduced according to the results of refs. ^31,49^.

### Ethics and inclusion statement

This study was performed with full participation of universities and institutes in Africa. The local researchers were involved in research design, sample collection and relevant data analyses. All participants who met the criteria have been included as co-authors, whereas those who made a partial contribution have all been included in the Acknowledgements. The sampling of endangered species (IUCN) in this study was carried out under the permission of the Malagasy government. To protect the endangered species, only fallen fruits were collected and seeds were further germinated to obtain leaves for further genomic studies. Local guides received fees for their services on sample collection and field surveys. Four Madagascar students were supported by this research programme as PhD or Master candidates at the University of Chinese Academy of Science.

### Reporting summary

Further information on research design is available in the Nature Portfolio Reporting Summary linked to this article.

## Online content

Any methods, additional references, Nature Portfolio reporting summaries, source data, extended data, supplementary information, acknowledgements, peer review information; details of author contributions and competing interests; and statements of data and code availability are available at 10.1038/s41586-024-07447-4.

### Supplementary information

Supplementary Information Supplementary Notes 1–3, Figs. 1–14 and Tables 1–11.

Reporting Summary

## Data Availability

The raw sequencing data reported in this paper are publicly available and have been deposited under accession number CRA015211 in the Genome Sequence Archive^109^ of the National Genomics Data Center^110^, China National Center for Bioinformation/Beijing Institute of Genomics, Chinese Academy of Sciences (GSA: CRA015211), which are publicly accessible at https://ngdc.cncb.ac.cn/gsa. Data of the assembly and annotation of eight baobab species are deposited in figshare at 10.6084/m9.figshare.25422502.v2 (ref. ^111^). The distribution data of baobabs used in our analyses are freely available in GBlF (10.15468/dl.ajuz23).
