# Supplementary material for: The rise of baobab trees in Madagascar
Source: Nature. 2024 May 15;629(8014):1091–9. doi: 10.1038/s41586-024-07447-4 (PMC11136661; doi:10.1038/s41586-024-07447-4)
Supplement: Supplementary file 1 — Supplementary Notes 1–3, Figs. 1–14 and Tables 1–11. [file 41586_2024_7447_MOESM1_ESM.pdf]

nature portfolio

https://doi.org/10.1038/s41586-024-07447-4

Supplementary information

The rise of baobab trees in Madagascar

In the format provided by the authors and unedited

Supplementary Note 1 | The genome size of baobabs and historical activity of long terminal retrotransposons

Adansonia species have relatively small estimated genome sizes (1C DNA content), ranging from 625 Mb (*A. rubrostipa*) to 736 Mb (*A. grandidieri*) with an average of 40,196 protein coding genes (**Figure 1** and **Table 1**). No obvious genome upsizing or downsizing was observed in *Adansonia* compared to its relatives in Malvaceae (based on data for 94 Malvaceae species taken from the Plant DNA C-Values Database[1], with the Malvaceae minimum, maximum and mean genome size being 188 Mb/1C, 8,281 Mb/1C and 1,500 Mb/1C). All species had abundant long terminal retrotransposons (LTRs) in their genomes (35.13% - 44.59%, **Table 2**), with evidence of recent bursts of activity in each species within the last two million years (My) **(Figure 2)**.

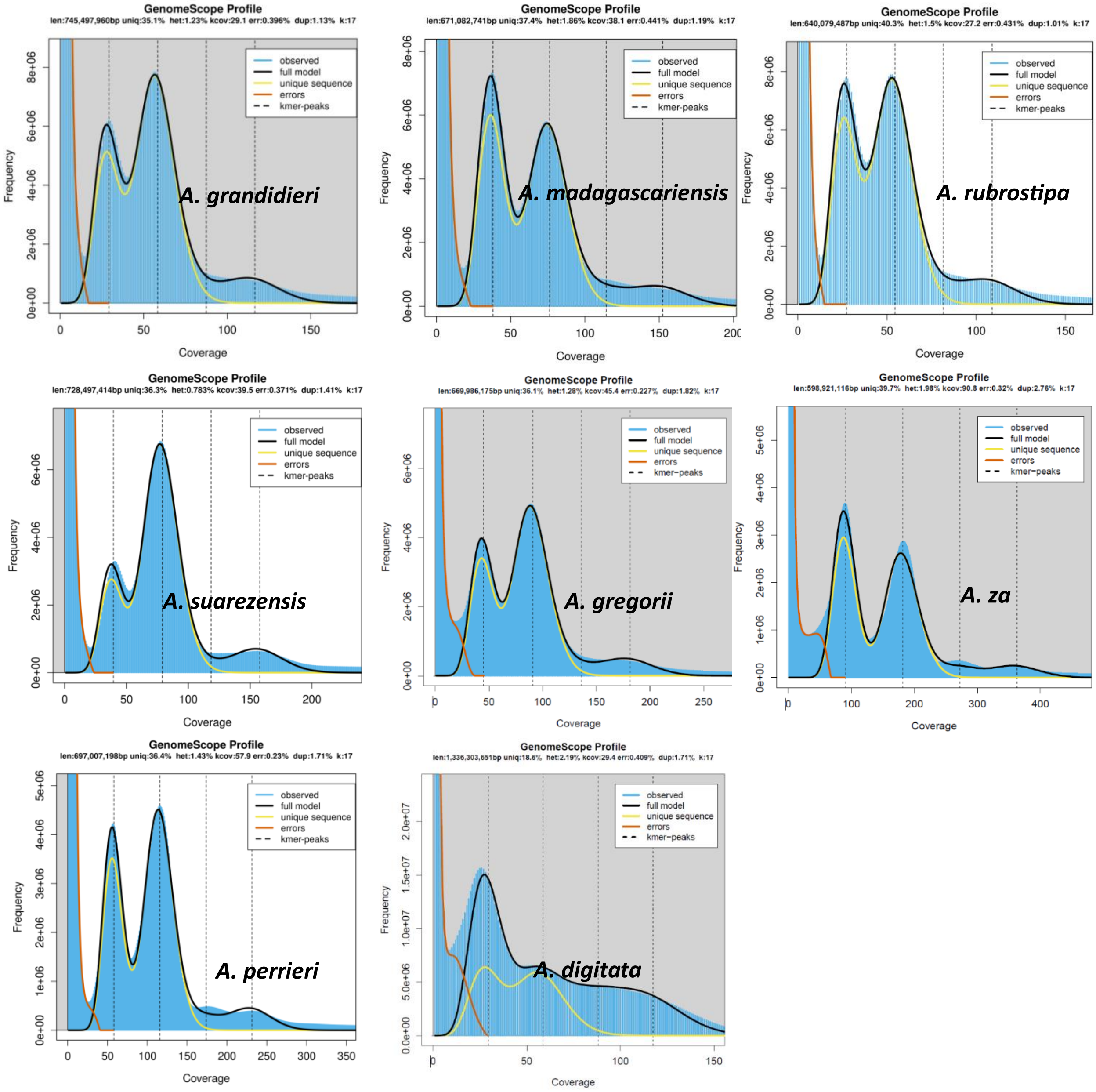

Figure 1 | GenomScope estimation of genome heterozygosity using 17-mer sequence counts from the baobab trees.

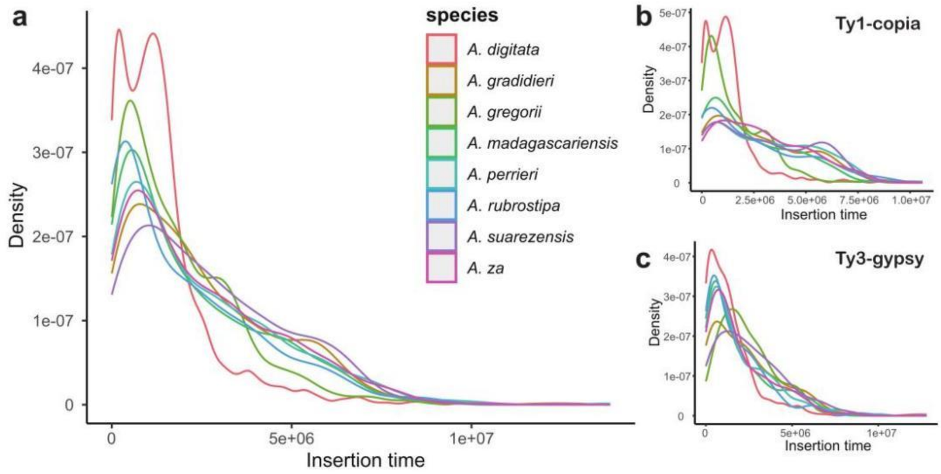

Figure 2 | The historical activity of long terminal repeat retrotransposons (LTR-RTs) in baobab genomes. **a,** Estimation of the activity of LTR-RTs showing a burst in activity in all baobab species within the last two million years. **b,** Separate analyses of Ty1-copia and Ty3-gypsy elements which represent the two major types of LTR-RTs in baobabs indicate that they both show evidence of recent bursts of activity.

Table 1 | The statistics of assembly and genomic characters.

**Species**	**Ploidy**	**Estimated Genome Size**	**Repeat Rate**	**Heterozygosity Rate***	**Assembled Size (bp)**	**Contig N50 (bp)**	**Gene Number**
A. grandidieri	diploid	745,497,960	64.90%	1.23%	735,789,292	3,349,818	39,821
A. madagascariensis	diploid	671,082,741	62.60%	1.86%	651,081,316	2,787,292	40,987
A. rubrostipa	diploid	640,079,487	59.70%	1.50%	625,135,551	3,217,624	40,332
A. suarezensis	diploid	728,497,414	63.70%	0.78%	707,244,577	4,720,927	39,551
A. gregorii	diploid	669,098,396	63.90%	1.27%	669,432,670	2,642,181	40,225
A. za	diploid	616,730,499	61.50%	1.87%	616,219,272	2,347,558	40,236
A. perrieri	diploid	697,007,198	63.33%	1.43%	695,430,667	16,661,966	40,012
A. digitata	tetraploid	1,336,303,655	81.40%	2.19%	668,573,124	1,015,549	40,403

Note: *A. perrieri* was sequenced with PacBio HiFi and assembled with Hifiasm. The other species were sequenced with PacBio CLR and assembled with Falcon. Heterozygosity Rate* was estimated using 17-*mers*.

Table 2 | The repeat sequence statistics and classification of long terminal repeats (LTR, number / length).

**Species**	***A. grandidieri***	***A. gregorii***	***A. madagascariensis***	***A. rubrostipa***	***A. suarezensis***	***A. za***	***A. digitata***	***A. perrieri***
Repeat (%)	60.04	49.57	52.81	52.99	58.49	48.74	47.42	58.23
LTR (%)	44.59	37.56	37.85	38.16	35.13	36.00	38.13	49.56
LTR number	171,931	173,288	209,184	191,794	170,759	234,592	205,794	252,462
LTR length (bp)	324,862,879	264,143,061	264,523,271	241,857,041	253,136,476	223,288,764	246,240,088	421,387,413
Ty1-Copia number	76,076	63,311	92,445	77,808	73,524	73,207	89,521	62,989
Ty1-Copia length (bp)	62,933,975	61,229,554	68,763,498	60,947,445	65,011,395	55,688,701	80,267,242	47,172,723
Ty3-Gypsy number	88,886	78,824	110,428	112,948	94,264	88,738	74,447	103,429
Ty3-Gypsy length (bp)	164,852,902	121,644,112	175,940,229	180,531,503	175,588,108	123,562,875	122,376,343	170,046,957

References

1. Pellicer, J. & Leitch, I. J. The Plant DNA C-values database (release 7.1): an updated online repository of plant genome size data for comparative studies. *New Phytol.* **226**, 301-305 (2020).

Supplementary Note 2 | Difficulty in resolving the origin of the stem group of *Adansonia*

According to a previous phylogenetic assessment in the subfamily Bombacoideae (Malvaceae), *Adansonia* was placed into a clade with four other extant Neotropical genera[1] with the plesiomorphy of a spongy endocarp (Adansonieae). However, the disjunct distribution of species within the clade has triggered debate as to the center of origin for *Adansonia*[1,2]. The fossil record of Bombacoideae[3] indicates a worldwide distribution of this subfamily between the Upper Cretaceous and the Palaeocene (84 - 53 Mya) (e.g. *Bombacoxylon gallettii*[3] in Ethiopia (Terminal Cretaceous-Eocene) and *Bombacoxylon langstonia*[4] in USA (Late Cretaceous)). However, the much younger stem age of *Adansonia* inferred here (~ 41.1 Mya) makes it difficult to determine the ancestral state of this genus without reliable fossils from Adansonieae.

References

1. Carvalho-Sobrinho, J. G., et al. Revisiting the phylogeny of Bombacoideae (Malvaceae): Novel relationships, morphologically cohesive clades, and a new tribal classification based on multilocus phylogenetic analyses. *Mol. Phylogenet. Evol.* **101**, 56-74 (2016).
2. Duarte, M. C., et al. Phylogenetic analyses of Eriotheca and related genera (Bombacoideae, Malvaceae). *Syst. Bot.* **36**, 690-701 (2011).
3. Kamal El-Din M. M. *Bombacoxylon owenii* (Carr.) Gottwald from Gebel Shabraweet, Eastern Desert, Egypt. *Taeckholmia* **22**, 91-99 (2002).
4. Wheeler & Lehman. Late Cretaceous woody dicots from the Ajuga and Javelina Formations, Big Bend National Park, Texas, USA. *IAWA J.* **21**, 86-120 (2004).

Supplementary Note 3 | Baobabs and animal pollination

We mapped the pollination syndromes of different *Adansonia* species onto the newly defined species tree, and combined this with data on the inferred evolutionary origins of their pollinators. Both generalized and specialized pollination occurs in baobabs, mediated by moths and mammals[1,2] (**Figure 1** and **Table 1**). Fruit bats (Pteropodidae) are thought to be the major pollinators of *A. digitata*[3] whereas the hawkmoth (*Agrius convolvuli*) and long-tongued bats pollinate *A. gregorii*[2]. Hawkmoths also pollinate species in *Longitubae* whereas the two *Brevitubae* species are pollinated by lemurs and fruit bats[2].

Both moths (Bombycoidea) and bats are involved in generalized pollination of species in Bombacoideae[4,5]. Moths are thought to have evolved c. 75 Mya[6] and bats c. 64 Mya[7]. Both events are earlier than the origin of Bombacoideae (~ 50 Mya) (**Fig. 1b**). Thus, *Adansonia* is likely to have diversified from ancestors with the plesiomorphic character of a generalized mode of moth/mammal pollination. This interpretation contradicts previous assertions of specialized pollination by hawkmoths[2]. We note that genes in the ANT (integrase-type DNA-binding superfamily protein) gene family, which control floral size (e.g. the size of floral tube and petal)[8], are significantly expanded in *Adansonia* (**Figure 2**) and similarly higher copy numbers of ANT were also found in *B. ceiba* (six copies). Potentially these genes provide a genetic basis for shaping the typically large flowers in Bombacoideae. Further genomic information and quantified pollination studies in this subfamily would help to determine if the larger floral size was driven by the most effective pollinators[9], as the larger body size of bats may lead to enlarged petals and/or the long-tongued hawkmoths to elongated floral tubes.

It is noteworthy that the current major animal pollinators of *Adansonia* are fruit bats and hawkmoths. The two animal lineages appeared at c. 25 Mya[4] and c. 15 Mya[10] respectively. Concurrently, the pollination system within baobabs seems to have switched with the divergence of the three geographic lineages (c. 21.6 Mya) (**Figure 1**). Similar switches in pollinators have been seen in other plant groups (e.g., *Dalechampia*[11]) and associated with trans-continental dispersal between Africa and Madagascar. Potentially, *Brevitubae* species decoupled ancestral interactions with hawkmoths with the shift of anthesis to the dry season from the rainy season seen in other baobab species[2] (**Figure 1**). Indeed, flowering phenology can be tightly associated with the activity of insect pollinators and influenced by small shifts in temperature (i.e. +/- 3-5 ℃)[12,13] (**Figure 1**). In addition, lemurs, which diversified in Madagascar over 60 Mya[14], are reported to provide a minor pollination service in *A. rubrostipa* and *A. za*[2]. More meticulous observations of *Longitubae* species during the rainy season are needed to determine if lemur pollination is indeed another plesiomorphic character of *Adansonia*.

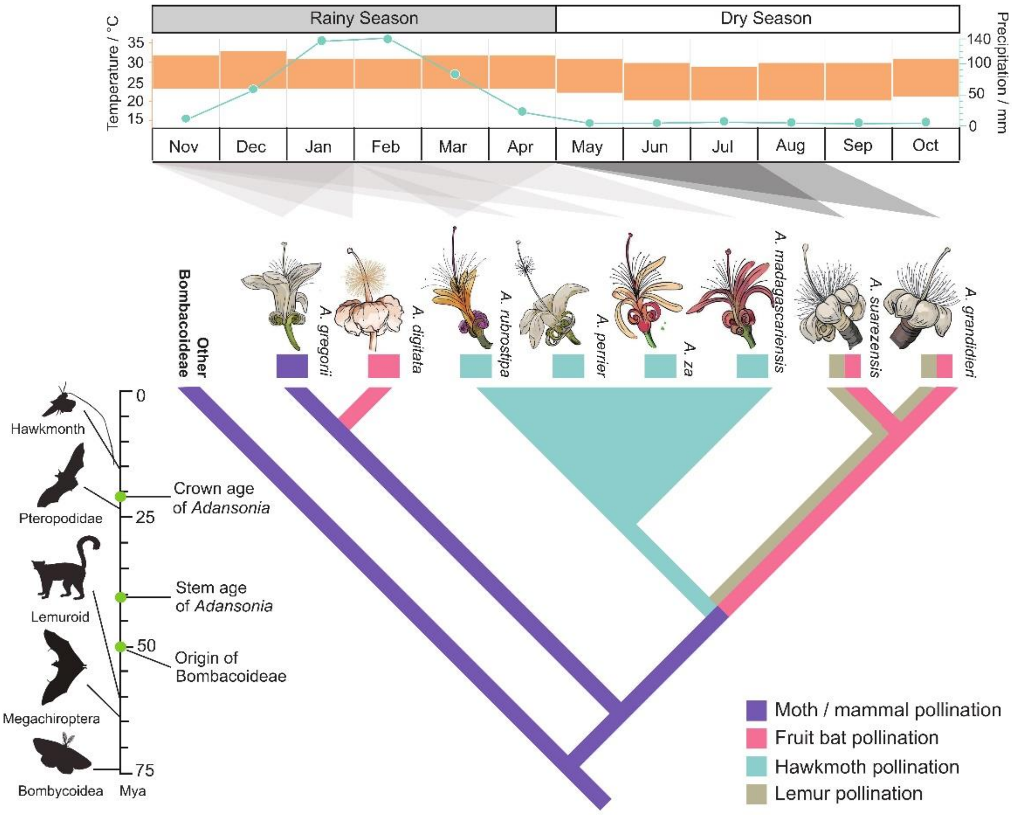

Figure 1 | The evolution of pollination ecology in *Adansonia*. The upper graph shows the monthly variation in temperature and precipitation over the rainy and dry seasons in Madagascar. The flowering duration of each baobab species is shown in the grey triangles that connect the upper graph with the species in the phylogeny below. The figure shows the later flowering phenology of *Brevitubae* (i.e. *A. suarezensis* and *A. grandidieri*) compared with the other species. The pollinator guilds are indicated by the branch colours on the *Adansonia* phylogeny. The timeline to the left of the phylogeny predicts the earliest occurrence of each pollinator guild, and the origins of *Adansonia* and the subfamily Bombacoideae to which *Adansonia* belongs.

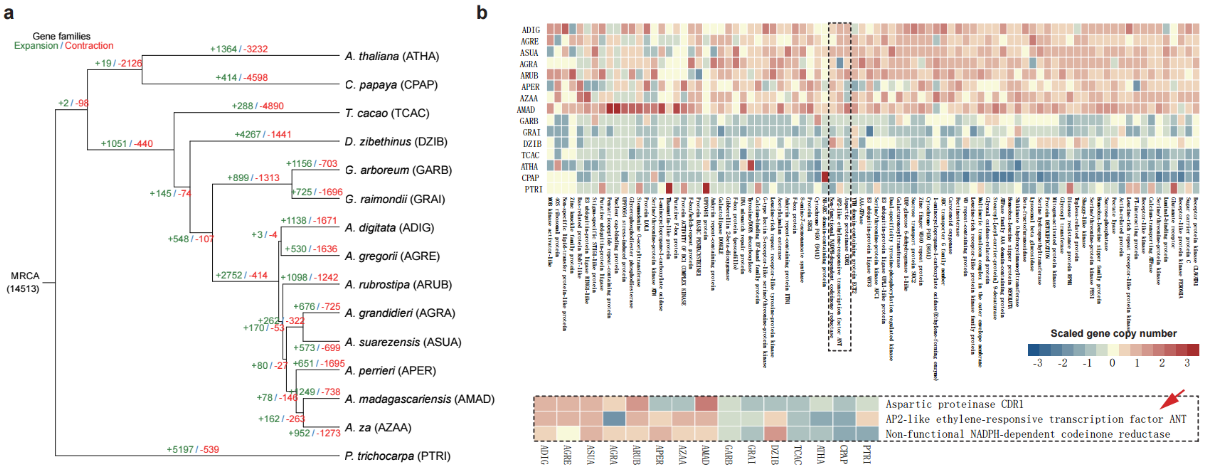

Figure 2 | Orthogroup divergence in *Adansonia*. a, The number of inferred gene gains (expansions in green) and losses (contractions in red) of orthogroups are indicated along the branches of the phylogeny. **b,** The GO terms for specific-expanded and contracted orthogroups in each species, with the four-letter acronyms for each species (row name) as defined in (**a**). The dashed box in (**b**) is expanded below the figure. The middle row shows the ANT (integrase-type DNA-binding superfamily protein) gene family (red arrow), a gene family that controls floral size and is expanded in all but one *Adansonia* species.

Table 1 | Documented pollinators of baobabs.

Baobab species	Pollinator
A. digitata[1, 2, 15, 16, 17, 18]	Straw-coloured fruit bat (*Eidolon helvum*)*<br>Gambian epauletted fruit bat (*Epomophorus gambianus gambiensis*)*<br>Peters' epaulette bat (*E. crypturus*, syn. *E. gambiensis crypturus*)*<br>Dog rousette bat (*Rousettus egyptiacus*)<br>Peters' dwarf epauletted fruit bat (*Micropteropus pusillus*)<br>Veld-kamp's dwarf epauletted bat (*Nanonycteris veldkampii*)<br>Epomorphorine bats (*Rousettus egyptiacus*)<br>Lesser bush baby (*Galago senegalensis*)<br>Greater bush baby (*Otolemur crassicaudatus*, syn. *Galago crassicaudatus*)<br>Bluebottles (*Chrysomyia marginalis*)<br>American bollworm (*Heliothis armigera*), red bollworm (*Diparopsis castanea*), spiny bollworm (*Earias biplaga*)
A. gregorii[16, 19]	Northern blossom bat (*Macroglossus minimus pygmaeus*)<br>Long-tongued bat (*Glossophaga* sp.)<br>Hawk moths (*Agrius convolvuli*)*<br>Honey-eaters (family Meliphagidae)<br>European honeybee (*Apis mellifera*)
A. grandidieri[16, 20]	Fork-marked lemur (*Phaner furcifer*)<br>Fat-tailed dwarf lemur (*Cheirogaleus medius*)<br>Coquerel's dwarf lemur (*Mirza coquereli*)<br>Honeybees (*Apis mellifera*), small sweat bees (*Trigona* spp.)<br>Madagascar straw-coloured fruit bat (*Eidolon dupreanum*)*<br>Hawk moths (*Nephele comma*)<br>Madagascar green sunbird (*Nectarinia notata*), Souimanga sunbird (*N. souimanga*)
A. suarezensis[16, 20, 21]	Madagascar straw-coloured fruit bat (*Eidolon dupreanum*)*<br>Madagascar flying fox (*Pteropus rufus*)<br>Amber mountain fork-tailed lemur (*Phaner furcifer electromontis*)
A. rubrostipa[1, 16]	Long-tongued hawk moths (*Coelonia solanii*)*<br>Fat-tailed dwarf lemur (*Cheirogaleus medius*)<br>Pale fork-marked lemur (*Phaner furcifer pallescens*)

A. perrieri[16]	Long-tongued sphingids (*Coelonia solani*, *Xanthopan morgani*)*
A. madagascariensis[1, 16, 20]	Long-tongued hawk moths (*Coelonia solanii*)<br>Comoros lesser flying fox (*Pteropus seychellensis comorensis*)
A. za[1, 16]	Long-tongued hawk moths (*Coelonia solanii*)<br>Two other hawk moths (*Coelonia brevis*, *Panogena jasminii*)<br>Pale forked-marked lemurs (*Phaner furcifer pallescens*)<br>Butterflies<br>Verreaux's sifaka (*Propithecus verreauxi verreauxi*)

Note: * indicates species are considered to be major pollinators.

References

1. Wickens, G. E. The Baobabs: pachycauls of Africa, Madagascar and Australia. Springer (2008).
2. Baum, D. A. The comparative pollination and floral biology of baobabs (*Adansonia* - Bombacaceae). *Ann. Mo. Bot. Gard.* **82**, 322-348 (1995).
3. Jaeger, P. Épanouissement et pollinisation de la fleur du baobab. Compte Rendu Hebd. *Séances Acad. Sci*. **220**, 369-371 (1945).
4. Kaisila, J. The Eqyptian fruit-bat, *Rousettus aegyptiacus* Geoffr. (Megachiroptera, Pteropodidae) visiting flowers of *Bombax malabaricum*. *Ann. Zool. Fenn.* **3**, 1-3 (1966).
5. Alverson, W. S. New species and combinations of *Catostemma* and *Pachira* (Bombacaceae) from the Venezuelan Guayana. *Novon* **4**, 3-8 (1994).
6. Kawahara, A. Y. et al. Phylogenomics reveals the evolutionary timing and pattern of butterflies and moths. *Proc. Natl Acad. Sci. USA*. **116**, 45 (2019).
7. Teeling, E. C. et al. A molecular phylogeny for bats illuminates biogeography and the fossil record. *Science* **307**, 580-584 (2005).
8. Manchado-Rojo, M., Weiss, J. & Egea-Cortines, M. Validation of Aintegumenta as a gene to modify floral size in ornamental plants. *Plant Biotech. J.* **12**, 1053-1065 (2014).
9. Rosas-Guerrero, V., Aguilar, R., Ashworth, L., Lopezarazia-Mikel, M. & Bastida, J. M. A quantitative review of pollination syndromes: do floral traits predict effective pollinators? *Ecol. Lett.* **17**, 388-400 (2014).
10. Almeida, F. C., Giannini, N. P. & Simmons, N. B. The evolutionary history of the African fruit bats (Chiroptera: Pteropodidae). *Acta Chiropt.* **18**, 73-90 (2016).
11. Armbruster, W. S. & Baldwin, B. G. Switch from specialized to generalized pollination. *Nature* **394**, 632 (1998).
12. Elzinga, J. A., Atlan, A., Biere, A., Gigord, L. & Weis, A. E. Time after time: flowering phenology and biotic interactions. *Trends Ecol. Evol*. **8**, 22 (2007).
13. Kehrberger, S. & Holzschuh, A. How does timing of flowering affect competition for pollinators, flower visitation and seed set in an early spring grassland plant? *Sci. Rep.* **9**, 15593 (2019).
14. Herrera, J. P. & Dávalos, L. M. Phylogeny and divergence times of lemurs inferred with recent and ancient fossils in the tree. *Syst. Biol.* **65**, 772-791 (2016).
15. Marshall, A. G. & McWilliam, A. Ecological observations on epomorphine fruit-bats

(Magachiroptera) in West African savanna woodland. *J Zoo* **198**, 3–67 (1982).
16. Baum, D. A. The comparative pollination and floral biology of baobabs (*Adansonia* - Bombacaceae). *Ann Mo Bot Gard* **82**, 322–348 (1995).
17. Coe, M. J. & Isaac, F. M. Pollination of the baobab (*Adansonia digitata* L.) by the lesser bushbaby (*Galagocras sicaudatus* E. Geoffroy). *E Afr Wildl J* **3**, 123–124 (1965).
18. McCann, C. Notes on the fulvous fruit bat (*Rousettus leschenaulti* Desm.). *J Bombay Nat Hist Soc* **41**, 805–816 (1940).
19. Lowe, P. *The Boab Tree*. Lothian Books, Port Melbourne (1998).
20. Andriafidison, F. et al. Nectarivory by endemic Malagasy fruit bats during the dry season. *Biotropica* **38**, 85–90 (2006).
21. Jolly, A. & Jolly, R. *Malagasy economics and conservation*. Pergamon Press, Oxford (1984).

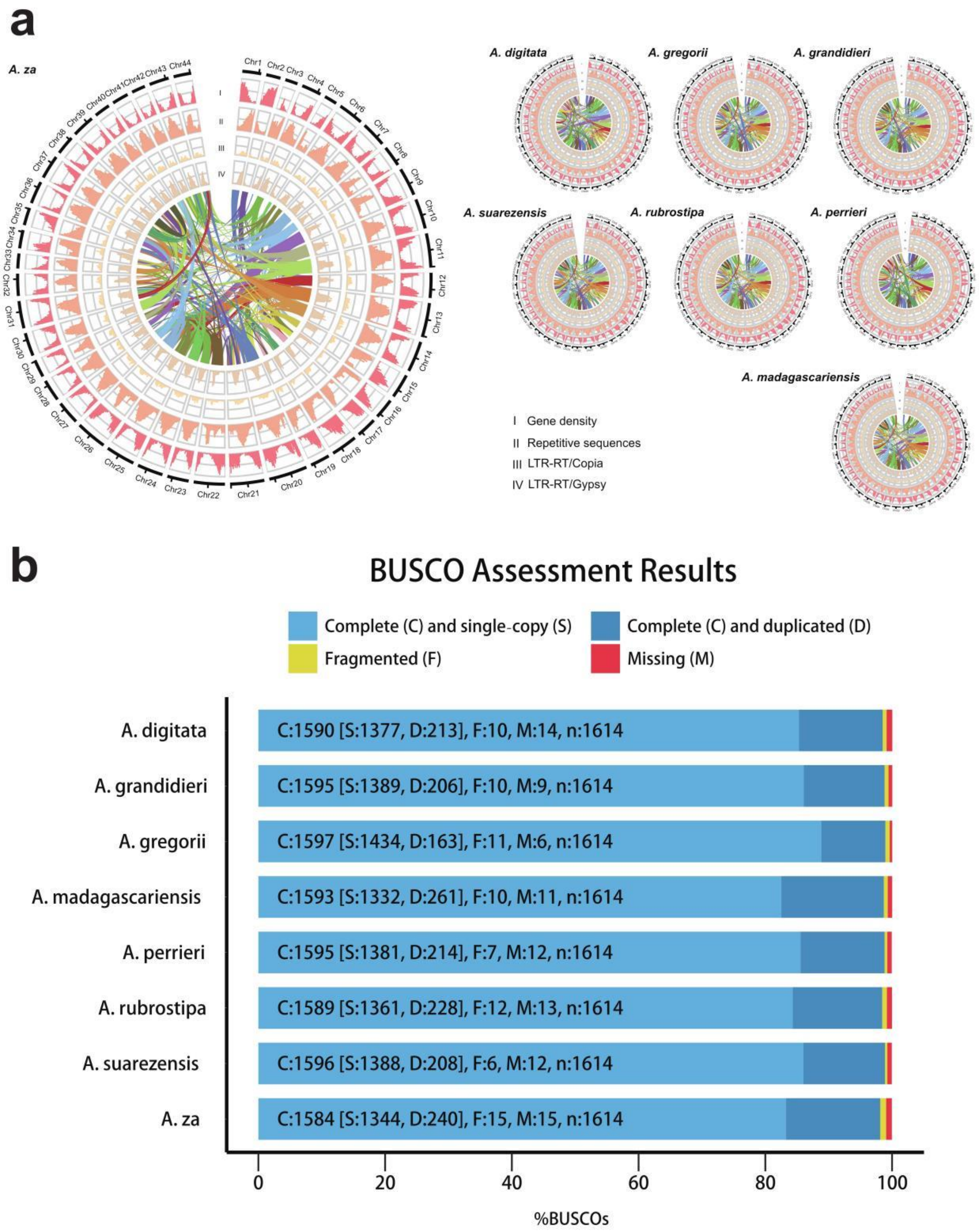

Supplementary Fig. 1 | The assembly of *Adansonia* genomes. **a**, Circos plots of each baobab species showing the genomic characters and intra-genomic collinearity of the chromosomes. The bands denote the collinear regions with paralogs on different chromosomes. **b**, The BUSCO (Benchmarking Universal Single-Copy Orthologs) analysis of the eight baobab genome assemblies.

A. grandidieri

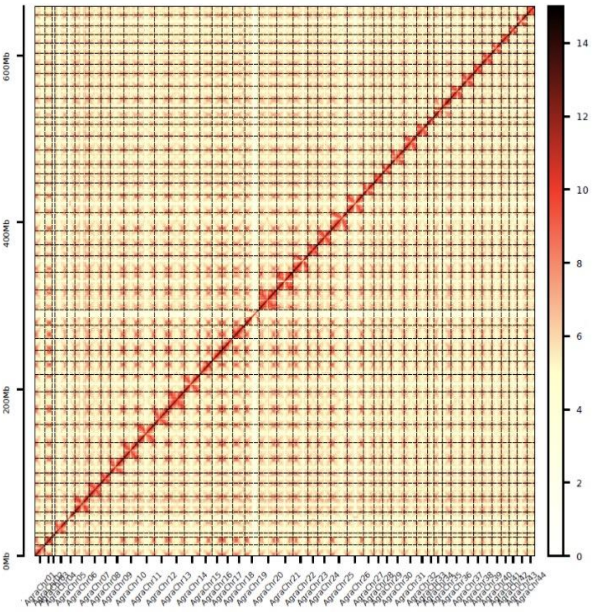

A. madagascariensis

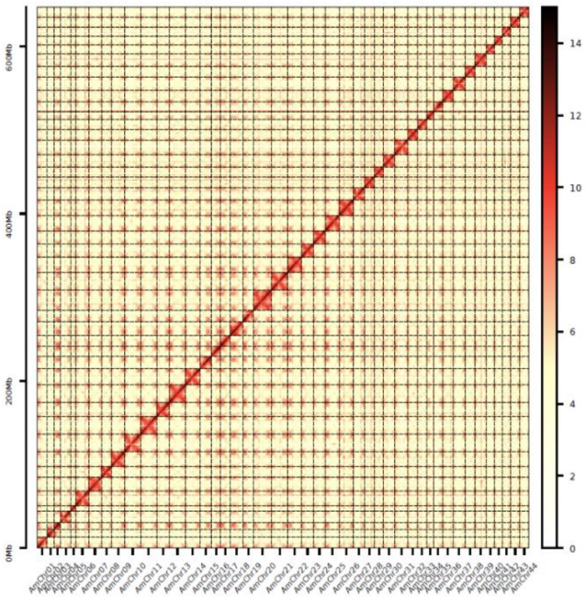

A. rubrostipa

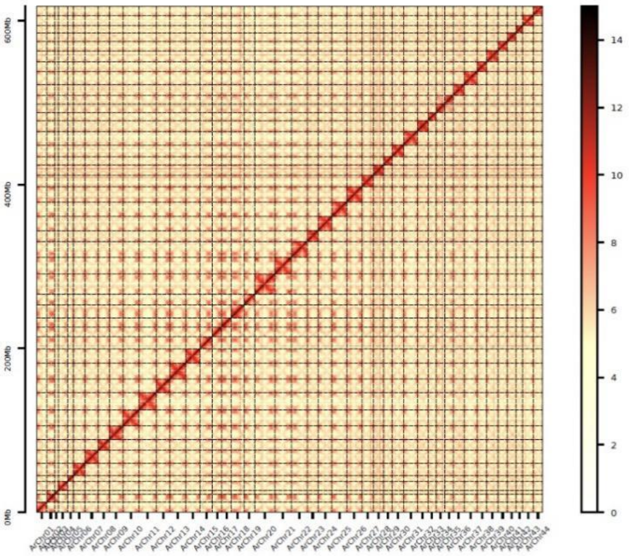

A. suarezensis

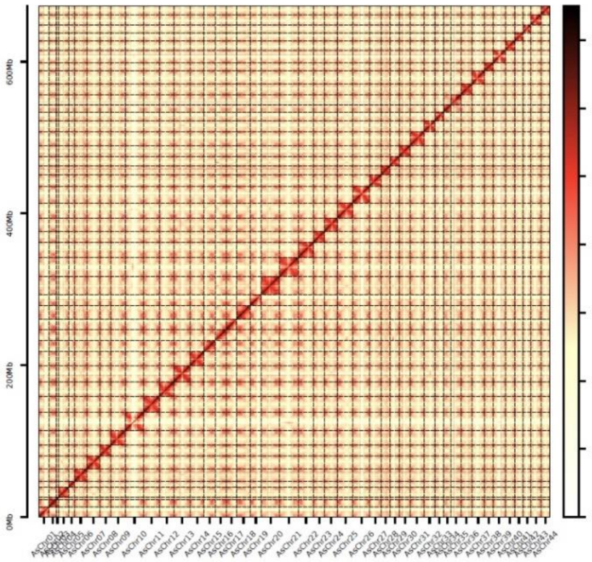

(Continued)

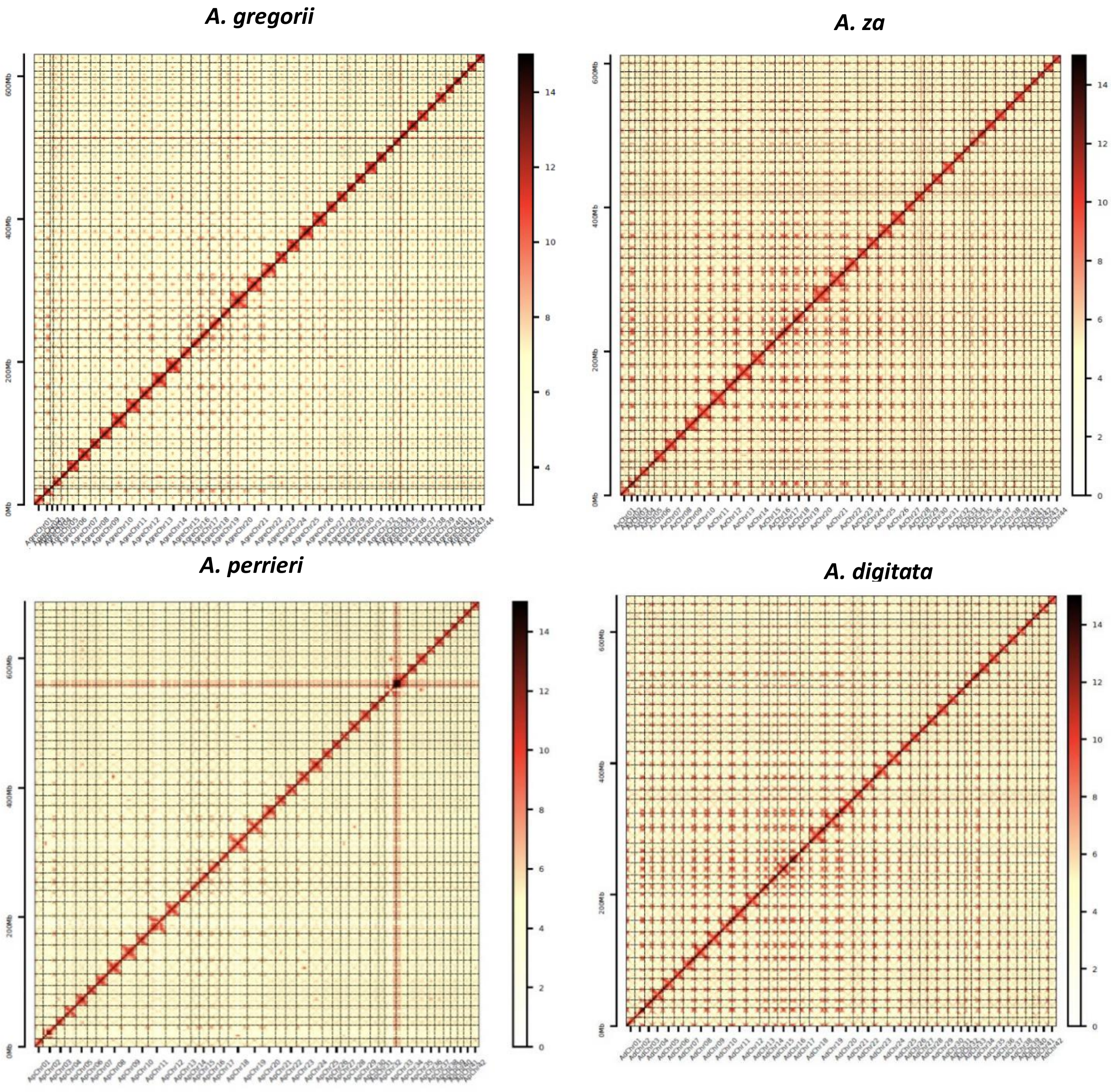

Supplementary Fig. 2 | The contact maps for chromosomes of the eight baobab species. X axis is chromosome number and Y axis the length of the chromosomes (Mb).

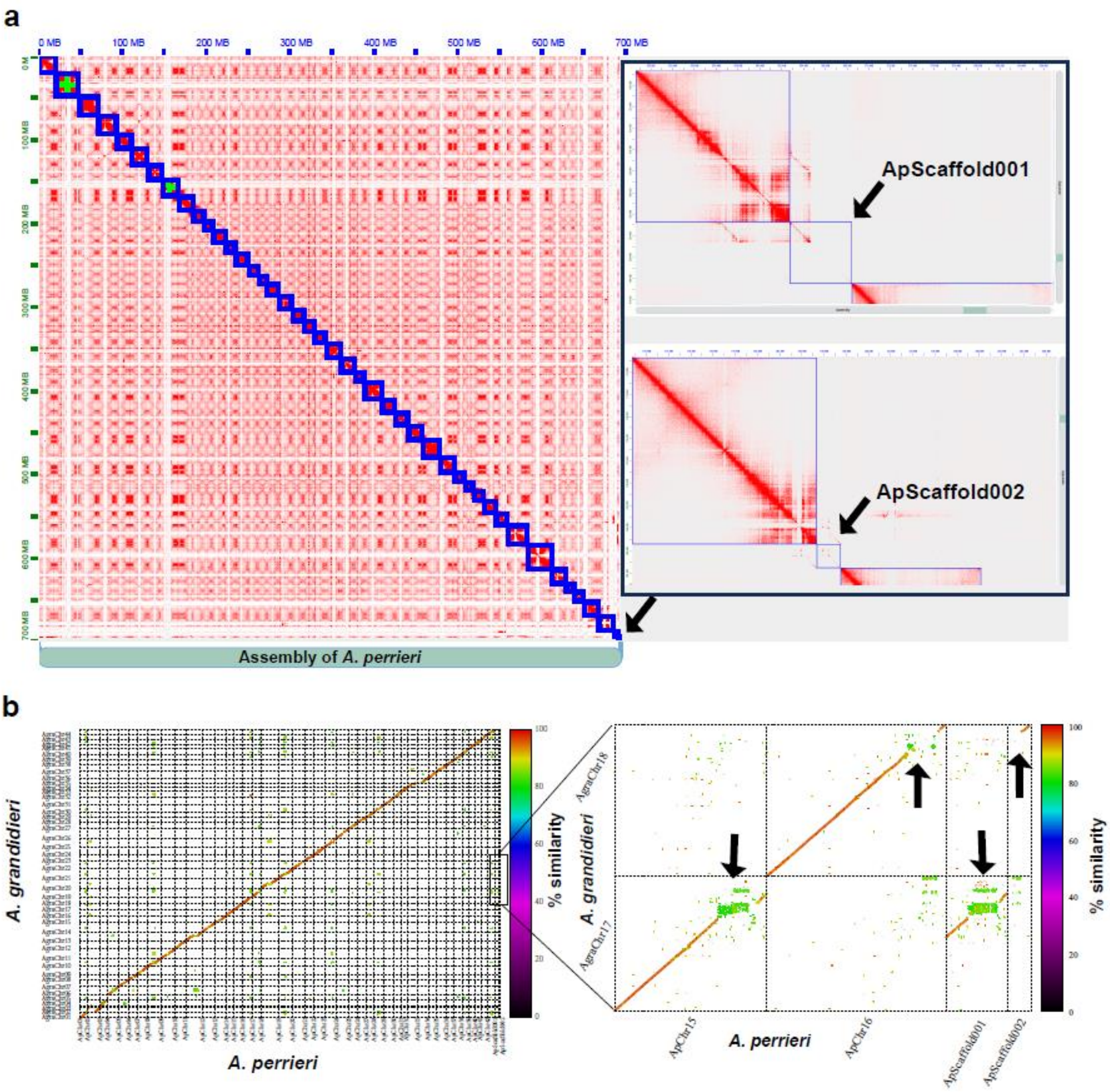

Supplementary Fig. 3 | The genomic inference of *n* = 42 in *A. perrieri*. **a,** The Hi-C contact map showed little evidence supporting the two small scaffolds as true chromosomes. The arrows point to the extremely weak signals in the contact inferences. **b,** The syntenic alignment showed that only 42 scaffolds of *A. perrieri* mapped well to the 44 scaffolds of *A. grandidieri*, indicating collinear inter-chromosomal relationships. The arrows highlight that the two small scaffolds of *A. perrieri* (i.e. ApScaffold001 and ApScaffold002) showed some sequence similarity to the well-defined chromosomes of *A. grandidieri* (i.e. AgraChr 17 and AgraChr 18) which have been verified to have collinear inter-chromosomal relationships with the chromosomes of *A. perrieri* (i.e. ApChr15 and ApChr16).

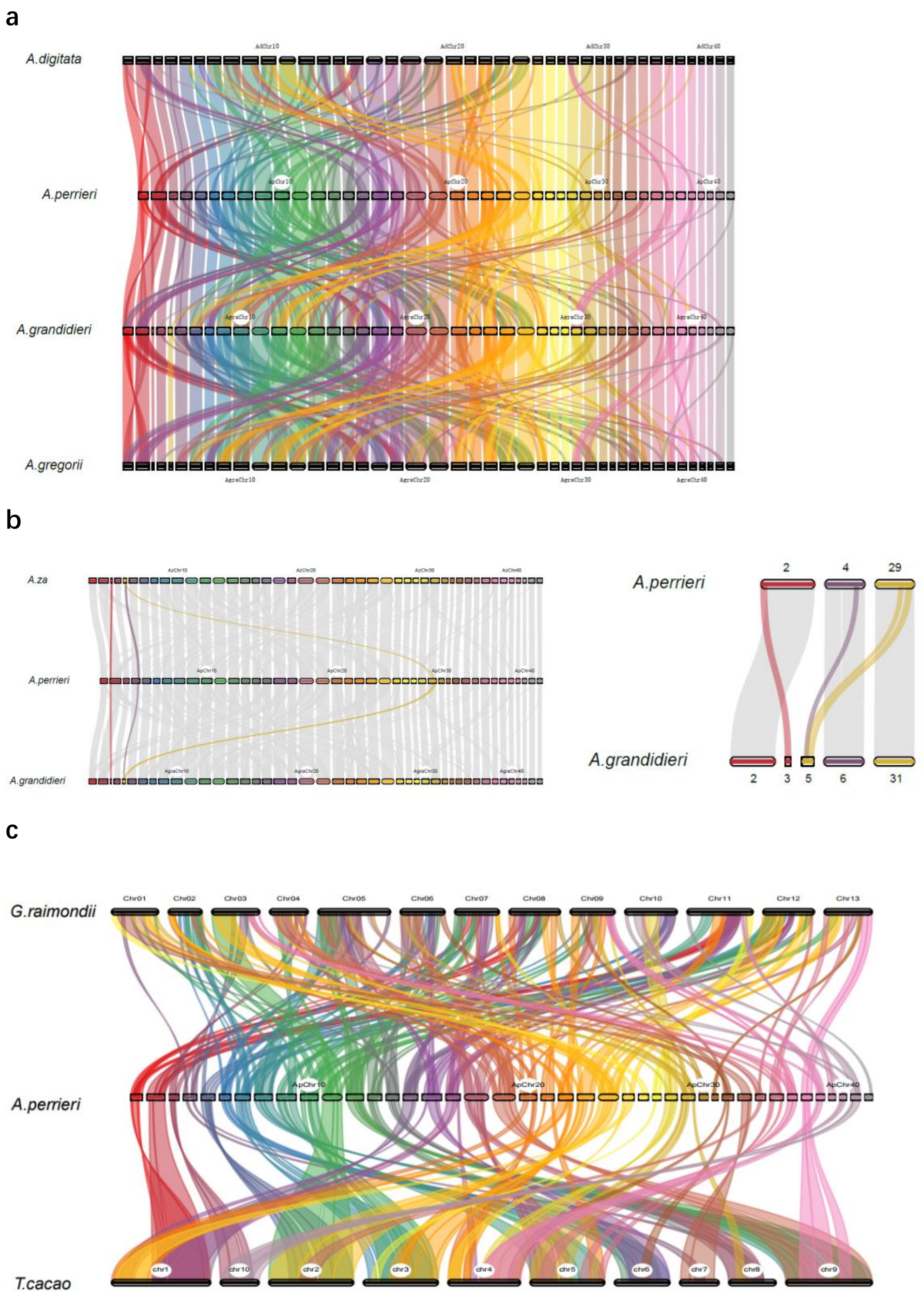

Supplementary Fig. 4 | Syntenic analysis within *Adansonia* and genome-wide comparisons to outgroup species. **a,** Macro-synteny alignments among *A. digitata*, *A. perrieri, A. grandidieri* and *A. gregorii*. **b**, Syntenic alignments indicate that there were probable chromosome rearrangements and fusions in the lineage leading to *A. perrieri*, which explains the two chromosome number difference between *A. perrieri* and the other diploid *Adansonia* species. **c,** Macro-synteny alignments between *A. perrieri* and outgroup Malvaceae species (i.e. *Gossypium raimondii* and *Theobroma cacao*). Detailed statistics of syntenic relationships are provided in **Supplementary Table 1**.

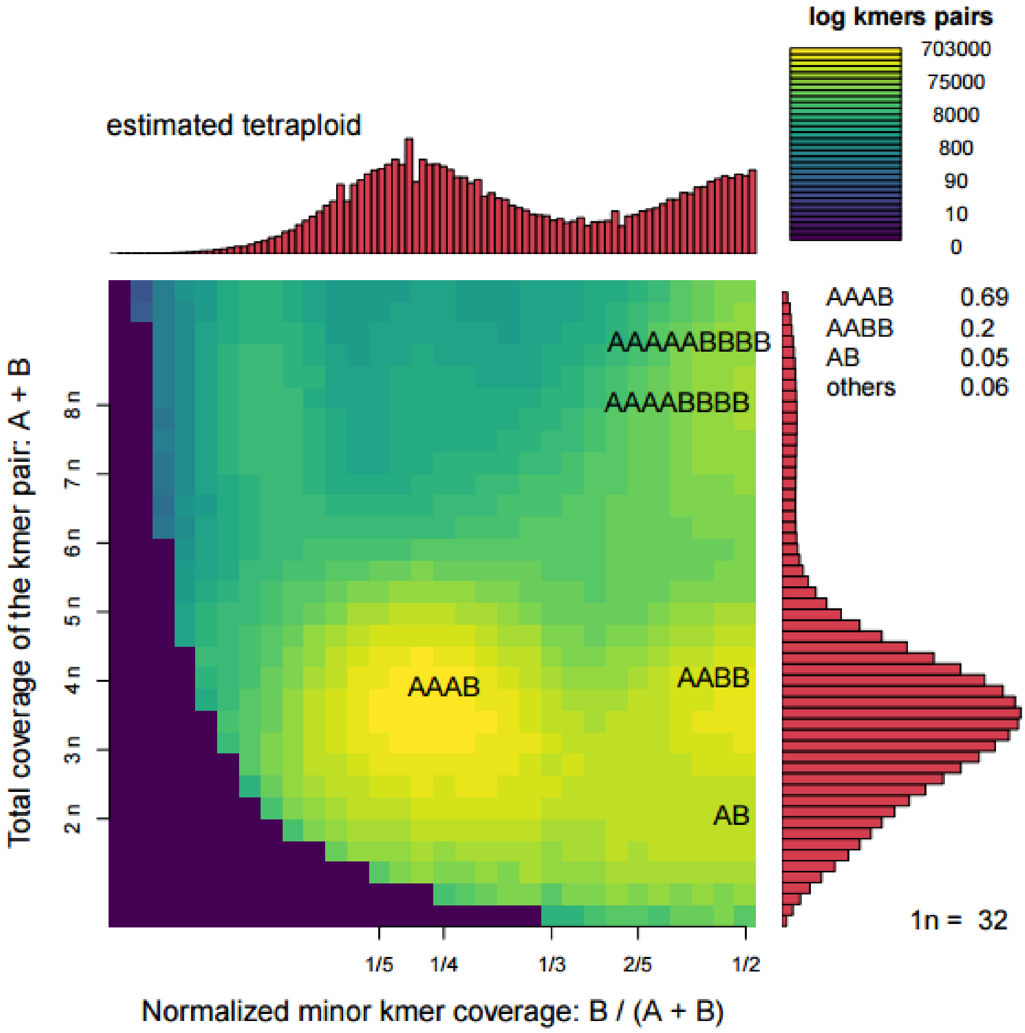

Supplementary Fig. 5 | Identification of *A. digitata* as an autopolyploid using Smudgeplot. Most of the *Kmer* pairs are distributed in the space (yellow) interpreted as indicating autopolyploidy (AAAB).

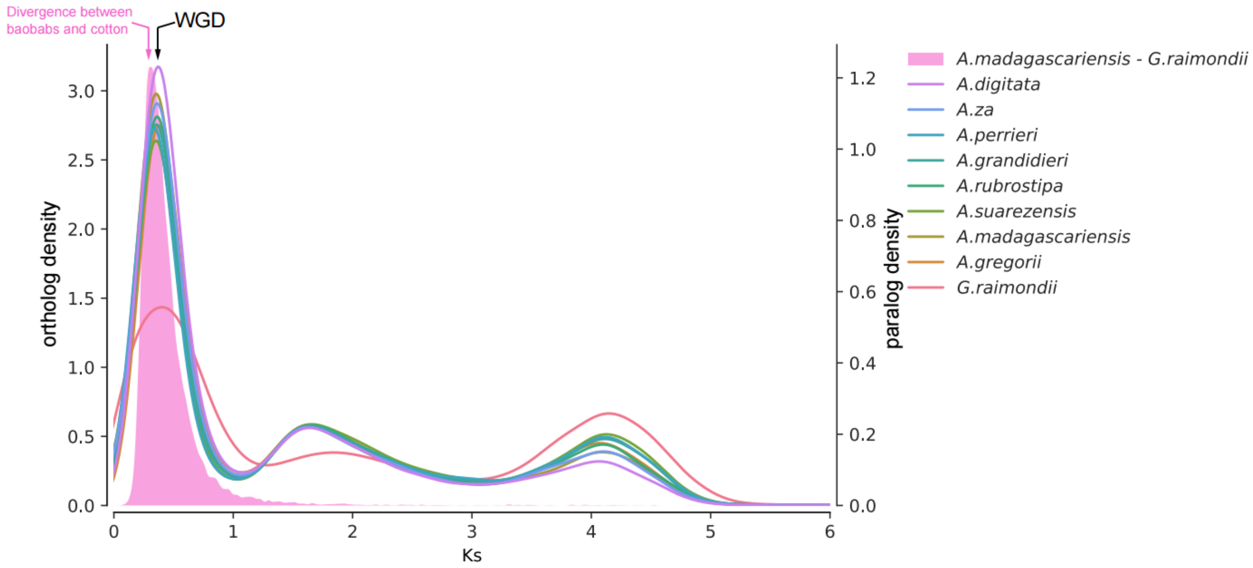

Supplementary Fig. 6 | The ancient whole genome duplication (WGD) event detected in *Adansonia*. The distributions of synonymous substitutions per synonymous site (Ks) for the whole paranome of each baobab species. Similar peak distributions between species indicate a shared WGD event occurred in their most recent common ancestor. For one-to-one orthologs between *A. madagascariensis* and *Gossypium raimondii*, the peak represents the species divergence events and Ks values correspond with the degree of orthologue divergence.

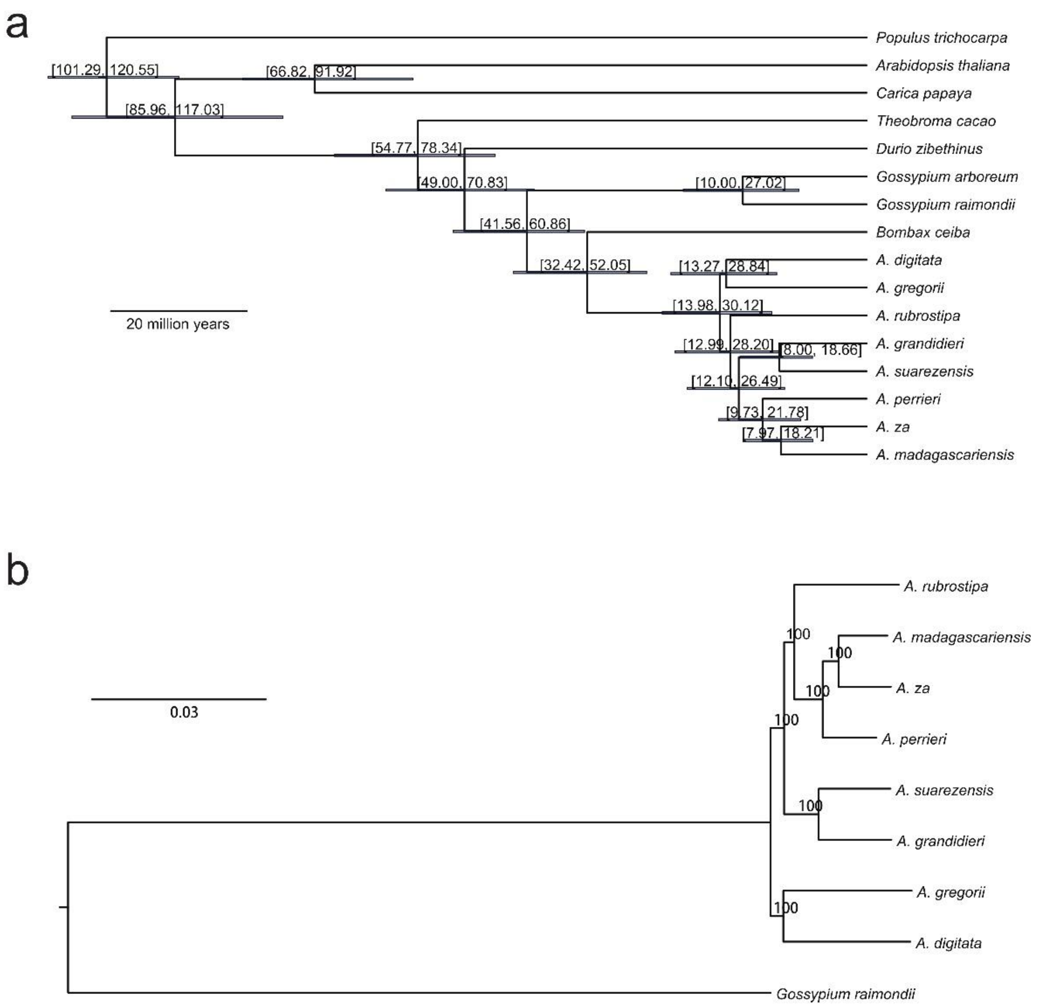

Supplementary Fig. 7 | **a,** The maximum likelihood tree from the concatenated datasets of baobabs. The maximum likelihood tree from the concatenation of 999 SCN genes and molecular dating of the divergence of baobabs, and **b,** phylogenetic inference from concatenation of the 26.31 Mb fragment.

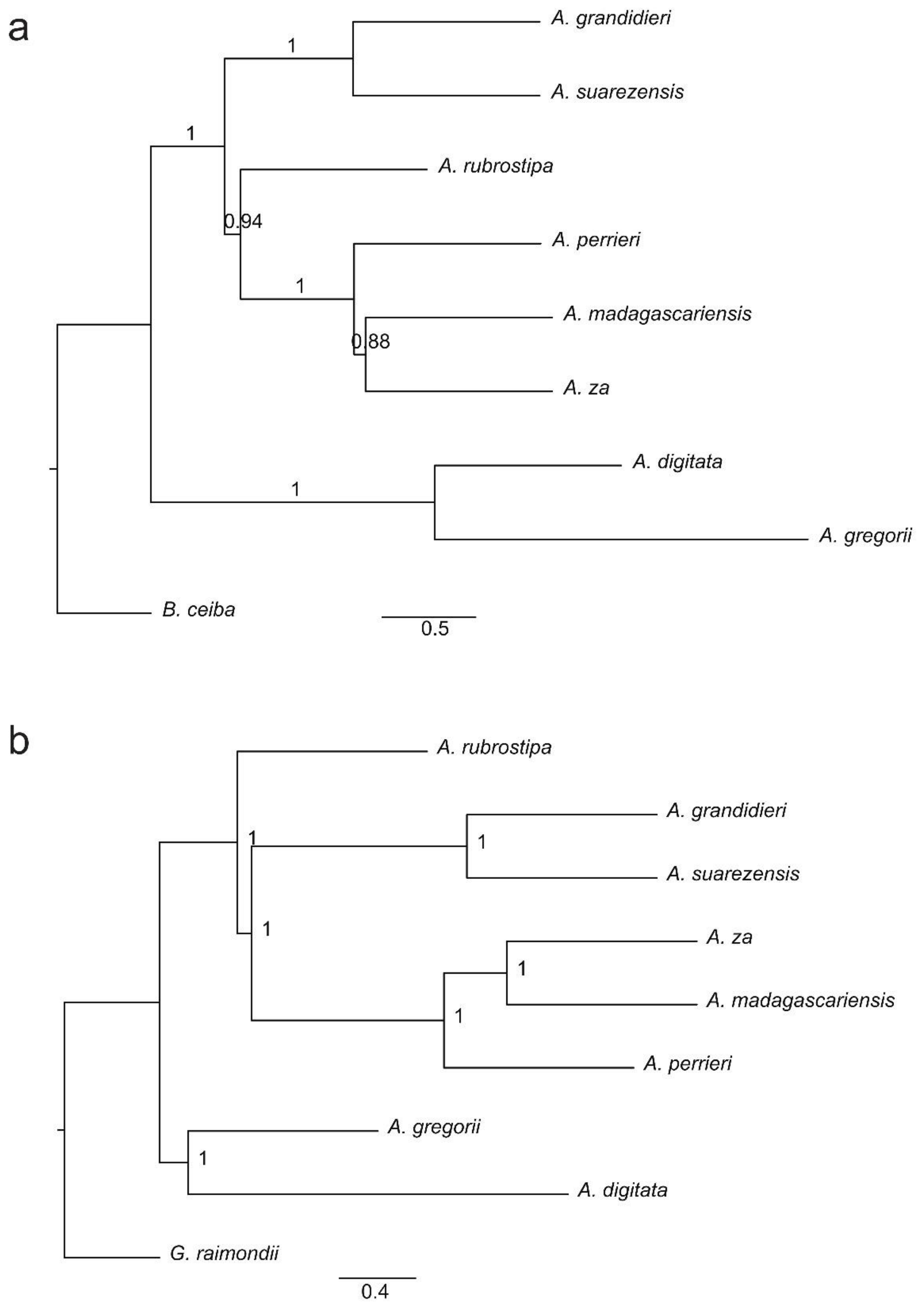

Supplementary Fig. 8 | a, The coalescence tree inferred with ASTRAL analysis using 999 SCN genes, and **b,** the coalescence tree inferred with ASTRAL using the full length of all synteny-guided genomic blocks.

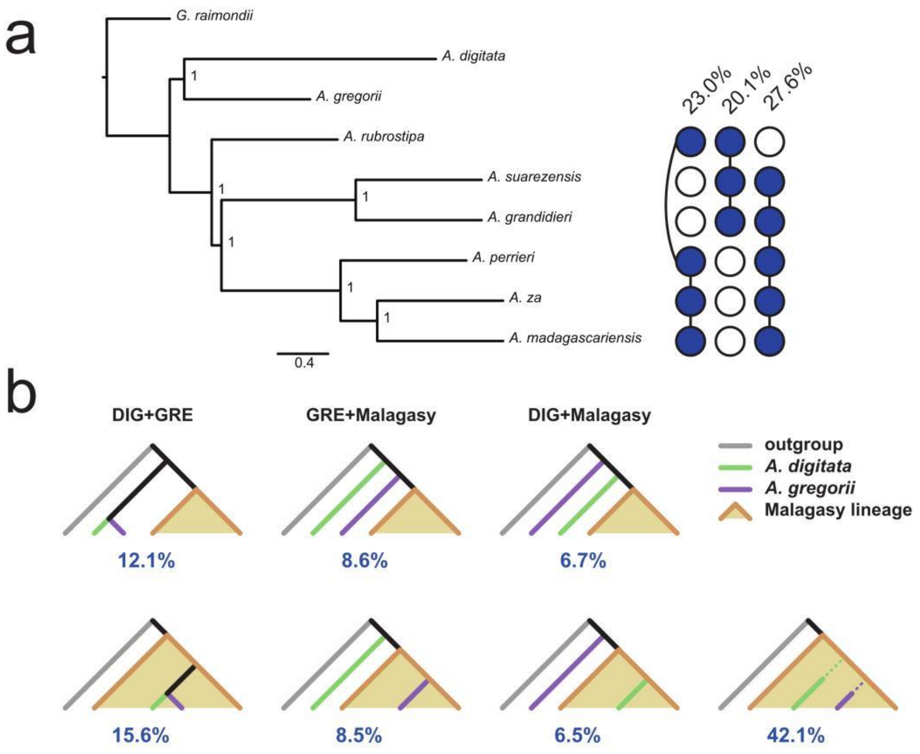

Supplementary Fig. 9 | Phylogenetic inference of small synteny-guided genomic blocks (2 kb). **a,** The commonest coalescence tree inferred from ASTRAL analysis and the summary of the percentage of gene tree topologies in this dataset are provided to reflect the conflicting relationships within the Malagasy clade. **b,** The statistics of conflicting maximum likelihood gene tree topologies of the three main *Adansonia* lineages.

a Ref. *A. digitata*

Africa lineage; Australia lineage; *Brevitubae*; *Longtitubae*

D-statistics outgroup: *A. gregorii* / *D*-statistics outgroup: *B. ceiba*	*A. digitata*	*A. gregorii*	*A. suarezensis*	*A. grandidieri*	*A. rubrostipa*	*A. perrieri*	*A. za*	*A. madagascariensis*
A. digitata								
A. gregorii	-0.042							
A. suarezensis	0.011	0.011		0.000	-0.056	0.003	0.000	0.002
A. grandidieri	0.003	-0.004	0.000		-0.043	0.000	-0.005	0.000
A. rubrostipa	0.002	-0.046	-0.058	-0.047		0.030	0.037	0.032
A. perrieri	0.005	-0.004	0.013	-0.002	0.033		0.021	-0.021
A. za	0.009	-0.003	0.008	-0.006	0.036	0.020		0.000
A. madagascariensis	0.012	-0.005	-0.003	-0.005	0.035	-0.020	0.000	

b Ref. *A. grandidieri*

Africa lineage; Australia lineage; *Brevitubae*; *Longtitubae*

D-statistics outgroup: *A. gregorii* / *D*-statistics outgroup: *B. ceiba*	*A. digitata*	*A. gregorii*	*A. suarezensis*	*A. grandidieri*	*A. rubrostipa*	*A. perrieri*	*A. za*	*A. madagascariensis*
A. digitata								
A. gregorii	0.000							
A. suarezensis	-0.093	-0.071		0.000	-0.052	0.037	0.036	0.048
A. grandidieri	-0.375	-0.337	0.000		-0.075	-0.009	-0.009	0.023
A. rubrostipa	-0.058	0.076	0.036	0.004		-0.009	0.008	0.001
A. perrieri	0.102	0.110	0.143	-0.099	-0.006		0.024	-0.024
A. za	0.107	0.111	0.140	-0.098	0.006	0.022		0.000
A. madagascariensis	0.098	0.109	0.141	-0.078	0.000	-0.022	0.000	

c Ref. *A. gregorii*

Africa lineage; Australia lineage; *Brevitubae*; *Longtitubae*

D-statistics outgroup: *A. gregorii* / *D*-statistics outgroup: *B. ceiba*	*A. digitata*	*A. gregorii*	*A. suarezensis*	*A. grandidieri*	*A. rubrostipa*	*A. perrieri*	*A. za*	*A. madagascariensis*
A. digitata								
A. gregorii	-0.102							
A. suarezensis	0.004	0.022		0.000	-0.051	0.014	0.012	-0.013
A. grandidieri	-0.004	0.012	0.000		-0.052	0.005	0.000	-0.019
A. rubrostipa	-0.002	0.013	-0.059	-0.047		0.036	0.049	0.017
A. perrieri	0.001	0.013	0.011	-0.002	0.033		0.039	-0.039
A. za	0.005	0.011	0.007	-0.004	0.039	0.020		0.000
A. madagascariensis	-0.004	0.031	-0.003	-0.009	0.034	-0.020	0.000	

d Ref. *A. madagascariensis*

Africa lineage; Australia lineage; *Brevitubae*; *Longtitubae*

D-statistics outgroup: *A. gregorii* / *D*-statistics outgroup: *B. ceiba*	*A. digitata*	*A. gregorii*	*A. suarezensis*	*A. grandidieri*	*A. rubrostipa*	*A. perrieri*	*A. za*	*A. madagascariensis*
A. digitata								
A. gregorii	0.000							
A. suarezensis	0.146	0.158		0.000	-0.023	0.021	0.011	-0.037
A. grandidieri	0.138	0.147	0.000		-0.012	0.023	0.011	-0.031
A. rubrostipa	0.143	0.157	-0.016	0.016		0.024	0.031	-0.021
A. perrieri	-0.048	-0.033	0.134	0.124	0.092		0.095	-0.095
A. za	-0.108	-0.096	0.067	0.059	0.034	0.000		0.000
A. madagascariensis	-0.318	-0.284	-0.159	-0.164	-0.187	-0.249	0.249	

e Ref. *A. perrieri*

Africa lineage; Australia lineage; *Brevitubae*; *Longtitubae*

D-statistics outgroup: *A. gregorii* / *D*-statistics outgroup: *B. ceiba*	*A. digitata*	*A. gregorii*	*A. suarezensis*	*A. grandidieri*	*A. rubrostipa*	*A. perrieri*	*A. za*	*A. madagascariensis*
A. digitata								
A. gregorii	0.000							
A. suarezensis	0.141	0.152		0.000	-0.023	-0.050	0.022	0.026
A. grandidieri	0.135	0.143	0.000		-0.013	-0.039	0.017	0.023
A. rubrostipa	0.140	0.152	-0.017	0.017		-0.041	0.042	0.036
A. perrieri	-0.345	-0.313	-0.182	-0.192	-0.220		0.000	0.000
A. za	-0.053	-0.043	0.114	0.102	0.084	0.001		0.000
A. madagascariensis	-0.062	-0.046	0.109	0.101	0.084	-0.001	0.000	

f Ref. *A. rubrostipa*

Africa lineage; Australia lineage; *Brevitubae*; *Longtitubae*

D-statistics outgroup: *A. gregorii* / *D*-statistics outgroup: *B. ceiba*	*A. digitata*	*A. gregorii*	*A. suarezensis*	*A. grandidieri*	*A. rubrostipa*	*A. perrieri*	*A. za*	*A. madagascariensis*
A. digitata								
A. gregorii	0.000							
A. suarezensis	0.007	0.029		0.000	-0.057	-0.001	-0.001	0.004
A. grandidieri	-0.004	0.014	0.000		-0.039	0.002	-0.001	0.002
A. rubrostipa	-0.363	-0.312	0.093	0.100		0.014	0.031	0.051
A. perrieri	-0.013	0.003	0.014	-0.003	0.154		0.024	-0.024
A. za	-0.012	0.000	0.008	-0.004	0.157	0.022		0.000
A. madagascariensis	0.055	-0.002	-0.003	-0.012	0.118	-0.022	0.000	

g Ref. *A. suarezensis*

Africa lineage; Australia lineage; *Brevitubae*; *Longtitubae*

D-statistics outgroup: *A. gregorii* / *D*-statistics outgroup: *B. ceiba*	*A. digitata*	*A. gregorii*	*A. suarezensis*	*A. grandidieri*	*A. rubrostipa*	*A. perrieri*	*A. za*	*A. madagascariensis*
A. digitata								
A. gregorii	0.000							
A. suarezensis	-0.384	-0.341		0.000	-0.088	-0.009	-0.007	0.023
A. grandidieri	-0.094	-0.075	0.000		-0.040	0.037	0.036	0.048
A. rubrostipa	0.061	0.079	-0.010	0.061		-0.012	0.010	0.002
A. perrieri	0.105	0.111	-0.094	0.137	-0.006		0.021	-0.021
A. za	0.110	0.112	-0.095	0.137	0.006	0.021		0.000
A. madagascariensis	0.101	0.111	-0.080	0.140	0.000	-0.021	0.000	

h Ref. *A. za*

Africa lineage; Australia lineage; *Brevitubae*; *Longtitubae*

D-statistics outgroup: *A. gregorii* / *D*-statistics outgroup: *B. ceiba*	*A. digitata*	*A. gregorii*	*A. suarezensis*	*A. grandidieri*	*A. rubrostipa*	*A. perrieri*	*A. za*	*A. madagascariensis*
A. digitata								
A. gregorii	0.000							
A. suarezensis	0.145	0.155		0.000	-0.021	0.021	-0.038	0.014
A. grandidieri	0.138	0.145	0.000		-0.012	0.023	-0.034	0.013
A. rubrostipa	0.141	0.154	-0.016	0.016		0.025	-0.015	0.024
A. perrieri	-0.051	-0.036	0.129	0.118	0.089		-0.054	0.054
A. za	-0.304	-0.276	-0.145	-0.154	-0.181	-0.245		0.000
A. madagascariensis	-0.116	-0.096	0.061	0.052	0.031	0.000	0.245	

Supplementary Fig. 10 | *D*-statistics calculated using different references. The dark boxes indicate undetectable introgression between species pairs. *D*-statistics were calculated from the analysis of introgression between baobab species (P2 = horizontal species and P3 = vertical species), where the value reported is the mean value calculated considering each species in turn as species P1 in the analysis. The reference genome assembly and the outgroup species used are indicated (**a**-**h**). See also **Supplementary Table 4**.

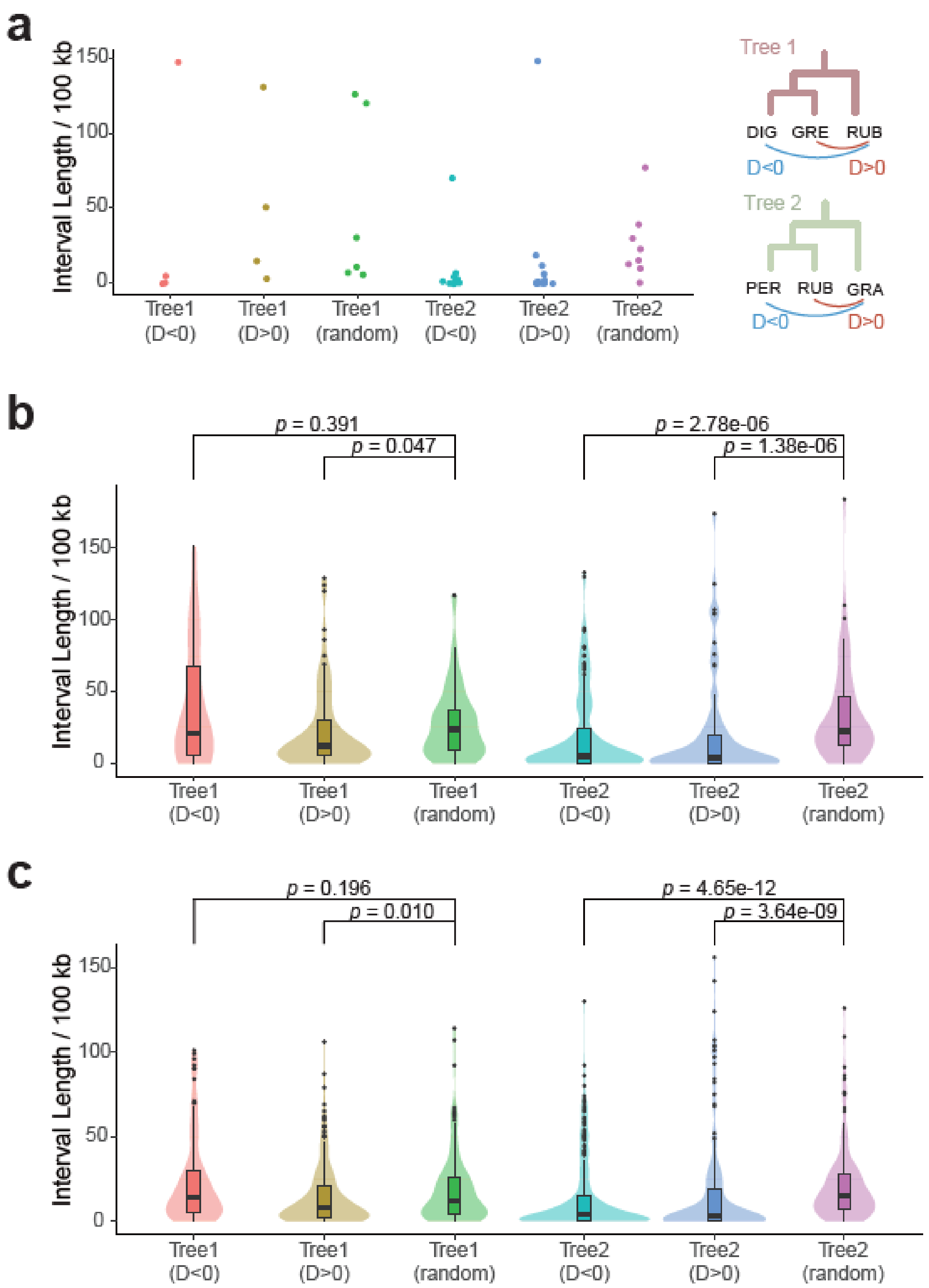

Supplementary Fig. 11 | Kernel density (violin) plots of the interval length between adjacent introgressed windows (100 kb), ranking in top 1% (**a**), top 5% (**b**) and top 10% (**c**) of the absolute *D* value. **a**, left to right, *n* = 4, 4, 6, 10, 12, 8; **b**, left to right, *n* = 43, 109, 63, 136, 71, 62; **c**, left to right, *n* = 116, 250, 157, 272, 142, 158. For **b** and **c**, one-sided Wilcoxon-test. In box plots, the centre line is the median, the box limits show the interquartile range (25th to 75th percentile), whiskers extend to quartiles ±1.5 × interquartile range, and dots show potential outliers.

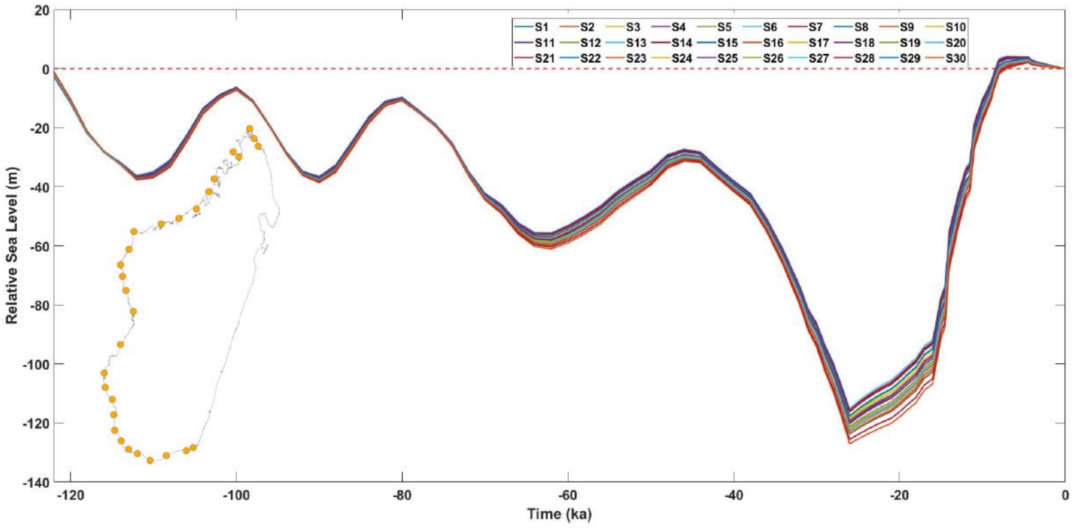

Supplementary Fig. 12 | The change in relative sea levels (RSL) along the shoreline of Madagascar from 0.12 Mya to present. The map shows the geographical locations of 30 randomly selected sampling sites (S1-S30) on the west coast of the island. The curves are predictions of local sea level at the 30 sites. The RSL simulation we have adopted is taken from Borreggine et al. (2023)[1].

References

1. Borreggine, M. et al. Sea-level rise in Southwest Greenland as a contributor to Viking abandonment. *Proc. Natl Acad. Sci. USA* **120**, e2209615120 (2023).

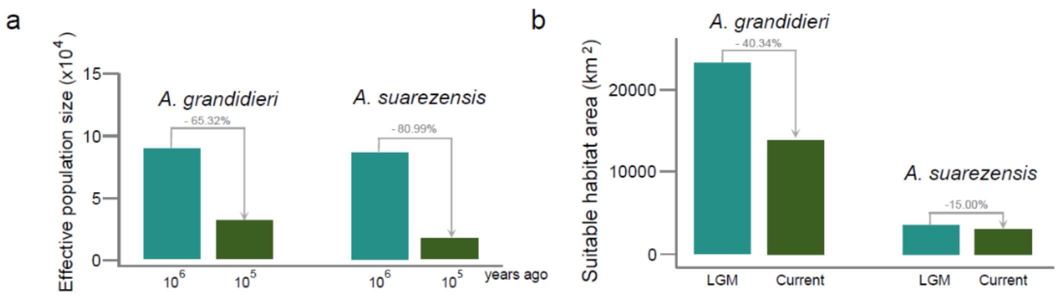

Supplementary Fig. 13 | The estimations of population size reductions for *A. suarezensis* and *A. grandidieri* in the past. **a,** historical changes in effective population size predicted from PSMC analysis (from 1 Mya to 0.1 Mya). **b,** the reduction in population size based on changes in area of potentially suitable habitats (from LGM (~ 22 kya) to present).

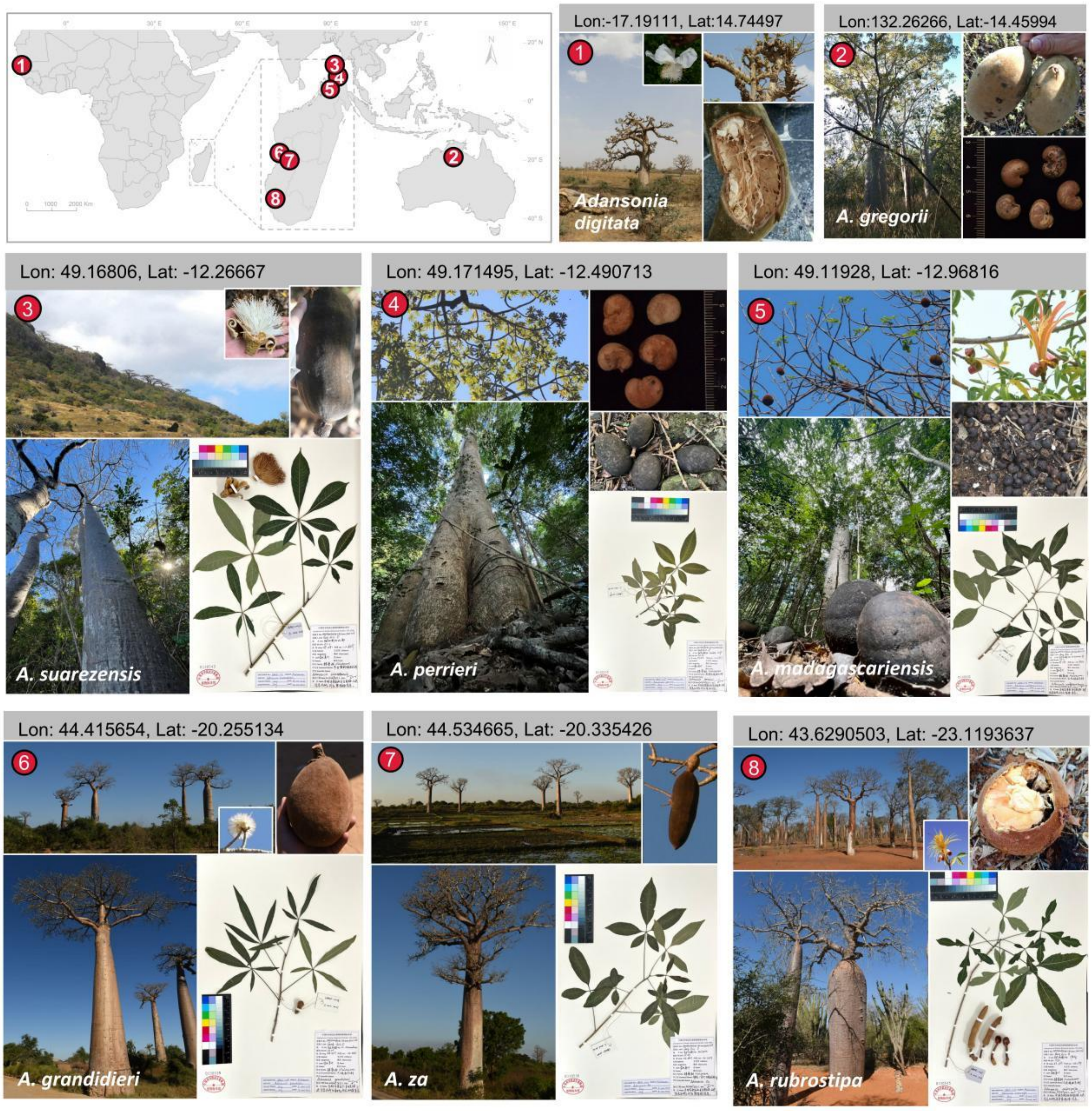

Supplementary Fig. 14 | Information on the individuals of eight baobab species sequenced. A view of the landscape of the natural population from which the individual was sampled, distribution locality, morphological traits as well as the voucher specimens.

Supplementary Table 1 | The statistics of syntenic blocks and genes within *Adansonia* species.

Species vs Species	Syntenic Blocks	Average Number of Syntenic Gene Pairs Per Block	Gene Pairs Included in Syntenic Block	Mean Block Length (bp)
A. digitata vs *A. digitata*	2,106	15	31,677	539,598
A. grandidieri vs *A. grandidieri*	2,546	16	40,965	841,754
A. gregorii vs *A. gregorii*	2,222	16	36,412	787,351
A. madagascariensis vs *A. madagascariensis*	2,404	17	39,876	752,718
A. perrieri vs *A. perrieri*	2,178	17	37,831	1,534,649
A. rubrostipa vs *A. rubrostipa*	2,526	16	39,982	771,915
A. suarezensis vs *A. suarezensis*	2,604	16	42,271	897,069
A. za vs *A. za*	2,349	16	38,394	747,147
A. digitata vs *A. madagascariensis*	1,107	72	79,487	1,019,130 / 958,017
A. grandidieri vs *A. madagascariensis*	1,096	73	79,656	999,548 / 1,002,735
A. gregorii vs *A. madagascariensis*	1,106	69	76,728	965,025 / 987,848
A. rubrostipa vs *A. madagascariensis*	1,085	73	79,532	980,783 / 986,446
A. suarezensis vs *A. madagascariensis*	1,085	74	79,760	1,002,823 / 1,010,827
A. za vs *A. madagascariensis*	955	78	74,859	1,030,605 / 1,035,653
A. perrieri vs *A. digitata*	1,121	70	78,444	1,119,492 / 1,113,840
A. perrieri vs *A. gregorii*	1,118	68	75,676	1,191,988 / 1,117,642

Supplementary Table 2 | The data sources of outgroup species used in this study.

Species	Links for accessing to data
Arabidopsis thaliana	https://phytozome-next.jgi.doe.gov/info/Athaliana_TAIR10
Bombax ceiba	https://gigadb.org/dataset/100445
Carica papaya	https://phytozome-next.jgi.doe.gov/info/Cpapaya_ASGPBv0_4
Durio zibethinus	https://www.ncbi.nlm.nih.gov/assembly/GCF_002303985.1/
Gossypium arboreum	https://www.cottongen.org/species/Gossypium_arboreum/A2_WHU
Gossypium raimondii	https://www.cottongen.org/species/Gossypium_raimondii/NSF-D5
Theobroma cacao	https://cocoa-genome-hub.southgreen.fr/download
Populus trichocarpa	https://phytozome-next.jgi.doe.gov/info/Ptrichocarpa_v3_1

Supplementary Table 3 | Counts of all lineage-specific duplications and deletions identified in baobabs.

DIG	GRE	SUA	GRA	RUB	PER	ZA	MAD	Duplication	Deletion
1	0	0	0	0	0	0	0	147	211
0	1	0	0	0	0	0	0	164	0
0	0	1	0	0	0	0	0	61	64
0	0	0	1	0	0	0	0	25	65
0	0	0	0	1	0	0	0	67	130
0	0	0	0	0	1	0	0	68	52
0	0	0	0	0	0	1	0	52	24
0	0	0	0	0	0	0	1	47	24
1	1	0	0	0	0	0	0	44	0
1	0	1	0	0	0	0	0	10	6
1	0	0	1	0	0	0	0	2	6
1	0	0	0	1	0	0	0	6	17
1	0	0	0	0	1	0	0	7	5
1	0	0	0	0	0	1	0	4	4
1	0	0	0	0	0	0	1	4	3
0	1	1	0	0	0	0	0	2	0
0	1	0	1	0	0	0	0	2	0
0	1	0	0	1	0	0	0	2	0
0	1	0	0	0	1	0	0	8	0
0	1	0	0	0	0	1	0	2	0
0	1	0	0	0	0	0	1	1	0
0	0	1	1	0	0	0	0	33	67
0	0	1	0	1	0	0	0	21	10
0	0	1	0	0	1	0	0	12	0
0	0	1	0	0	0	1	0	2	1
0	0	1	0	0	0	0	1	4	0
0	0	0	1	1	0	0	0	3	11
0	0	0	1	0	1	0	0	9	3
0	0	0	1	0	0	1	0	2	1
0	0	0	1	0	0	0	1	3	1
0	0	0	0	1	1	0	0	11	6
0	0	0	0	1	0	1	0	15	3
0	0	0	0	1	0	0	1	3	3
0	0	0	0	0	1	1	0	28	8
0	0	0	0	0	1	0	1	26	7
0	0	0	0	0	0	1	1	32	27
1	1	1	0	0	0	0	0	1	0
1	1	0	1	0	0	0	0	0	0
1	1	0	0	1	0	0	0	0	0
1	1	0	0	0	1	0	0	5	0
1	1	0	0	0	0	1	0	0	0
1	1	0	0	0	0	0	1	0	0
1	0	1	1	0	0	0	0	8	17
1	0	1	0	1	0	0	0	4	5
1	0	1	0	0	1	0	0	1	0

1	0	1	0	0	0	1	0	0	0
1	0	1	0	0	0	0	1	1	0
1	0	0	1	1	0	0	0	2	1
1	0	0	1	0	1	0	0	0	2
1	0	0	1	0	0	1	0	0	0
1	0	0	1	0	0	0	1	0	0
1	0	0	0	1	1	0	0	1	0
1	0	0	0	1	0	1	0	1	2
1	0	0	0	1	0	0	1	1	0
1	0	0	0	0	1	1	0	2	2
1	0	0	0	0	1	0	1	1	1
1	0	0	0	0	0	1	1	7	7
0	1	1	1	0	0	0	0	1	0
0	1	1	0	1	0	0	0	2	0
0	1	1	0	0	1	0	0	2	0
0	1	1	0	0	0	1	0	1	0
0	1	1	0	0	0	0	1	0	0
0	1	0	1	1	0	0	0	0	0
0	1	0	1	0	1	0	0	1	0
0	1	0	1	0	0	1	0	0	0
0	1	0	1	0	0	0	1	0	0
0	1	0	0	1	1	0	0	1	0
0	1	0	0	1	0	1	0	2	0
0	1	0	0	1	0	0	1	0	0
0	1	0	0	0	1	1	0	1	0
0	1	0	0	0	1	0	1	2	0
0	1	0	0	0	0	1	1	3	0
0	0	1	1	1	0	0	0	20	17
0	0	1	1	0	1	0	0	3	13
0	0	1	1	0	0	1	0	3	2
0	0	1	1	0	0	0	1	3	4
0	0	1	0	1	1	0	0	5	2
0	0	1	0	1	0	1	0	2	0
0	0	1	0	1	0	0	1	4	1
0	0	1	0	0	1	1	0	13	1
0	0	1	0	0	1	0	1	7	3
0	0	1	0	0	0	1	1	12	0
0	0	0	1	1	1	0	0	0	0
0	0	0	1	1	0	1	0	6	1
0	0	0	1	1	0	0	1	1	2
0	0	0	1	0	1	1	0	1	0
0	0	0	1	0	1	0	1	3	0
0	0	0	1	0	0	1	1	1	1
0	0	0	0	1	1	1	0	3	0
0	0	0	0	1	1	0	1	11	6
0	0	0	0	1	0	1	1	13	3
0	0	0	0	0	1	1	1	61	54
1	1	1	1	0	0	0	0	7	0
1	1	1	0	1	0	0	0	0	0
1	1	1	0	0	1	0	0	2	0
1	1	1	0	0	0	1	0	0	0

1	1	1	0	0	0	0	1	0	0
1	1	0	1	1	0	0	0	0	0
1	1	0	1	0	1	0	0	0	0
1	1	0	1	0	0	1	0	0	0
1	1	0	1	0	0	0	1	0	0
1	1	0	0	1	1	0	0	1	0
1	1	0	0	1	0	1	0	0	0
1	1	0	0	1	0	0	1	0	0
1	1	0	0	0	1	1	0	0	0
1	1	0	0	0	1	0	1	0	0
1	1	0	0	0	0	1	1	1	0
1	0	1	1	1	0	0	0	5	7
1	0	1	1	0	1	0	0	4	2
1	0	1	1	0	0	1	0	0	1
1	0	1	1	0	0	0	1	0	1
1	0	1	0	1	1	0	0	1	1
1	0	1	0	1	0	1	0	0	2
1	0	1	0	1	0	0	1	0	0
1	0	1	0	0	1	1	0	1	1
1	0	1	0	0	1	0	1	0	0
1	0	1	0	0	0	1	1	3	1
1	0	0	1	1	1	0	0	0	1
1	0	0	1	1	0	1	0	0	1
1	0	0	1	1	0	0	1	0	0
1	0	0	1	0	1	1	0	0	0
1	0	0	1	0	1	0	1	0	1
1	0	0	1	0	0	1	1	1	0
1	0	0	0	1	1	1	0	0	1
1	0	0	0	1	1	0	1	0	1
1	0	0	0	1	0	1	1	3	2
1	0	0	0	0	1	1	1	11	19
0	1	1	1	1	0	0	0	2	0
0	1	1	1	0	1	0	0	7	0
0	1	1	1	0	0	1	0	1	0
0	1	1	1	0	0	0	1	2	0
0	1	1	0	1	1	0	0	0	0
0	1	1	0	1	0	1	0	0	0
0	1	1	0	1	0	0	1	0	0
0	1	1	0	0	1	1	0	0	0
0	1	1	0	0	1	0	1	1	0
0	1	1	0	0	0	1	1	1	0
0	1	0	1	1	1	0	0	1	0
0	1	0	1	1	0	1	0	0	0
0	1	0	1	1	0	0	1	0	0
0	1	0	1	0	1	1	0	0	0
0	1	0	1	0	1	0	1	0	0
0	1	0	1	0	0	1	1	0	0
0	1	0	0	1	1	1	0	0	0
0	1	0	0	1	1	0	1	1	0
0	1	0	0	1	0	1	1	0	0
0	1	0	0	0	1	1	1	6	0

0	0	1	1	1	1	0	0	7	3
0	0	1	1	1	0	1	0	3	2
0	0	1	1	1	0	0	1	3	1
0	0	1	1	0	1	1	0	3	0
0	0	1	1	0	1	0	1	10	0
0	0	1	1	0	0	1	1	12	4
0	0	1	0	1	1	1	0	6	1
0	0	1	0	1	1	0	1	7	0
0	0	1	0	1	0	1	1	7	2
0	0	1	0	0	1	1	1	32	3
0	0	0	1	1	1	1	0	6	1
0	0	0	1	1	1	0	1	0	1
0	0	0	1	1	0	1	1	0	1
0	0	0	1	0	1	1	1	10	7
0	0	0	0	1	1	1	1	47	26
1	1	1	1	1	0	0	0	0	0
1	1	1	1	0	1	0	0	11	0
1	1	1	1	0	0	1	0	1	0
1	1	1	1	0	0	0	1	1	0
1	1	1	0	1	1	0	0	1	0
1	1	1	0	1	0	1	0	0	0
1	1	1	0	1	0	0	1	0	0
1	1	1	0	0	1	1	0	0	0
1	1	1	0	0	1	0	1	2	0
1	1	1	0	0	0	1	1	0	0
1	1	0	1	1	1	0	0	1	0
1	1	0	1	1	0	1	0	0	0
1	1	0	1	1	0	0	1	0	0
1	1	0	1	0	1	1	0	0	0
1	1	0	1	0	1	0	1	0	0
1	1	0	1	0	0	1	1	1	0
1	1	0	0	1	1	1	0	0	0
1	1	0	0	1	1	0	1	0	0
1	1	0	0	1	0	1	1	0	0
1	1	0	0	0	1	1	1	4	0
1	0	1	1	1	1	0	0	6	0
1	0	1	1	1	0	1	0	5	1
1	0	1	1	1	0	0	1	1	0
1	0	1	1	0	1	1	0	3	1
1	0	1	1	0	1	0	1	0	1
1	0	1	1	0	0	1	1	4	3
1	0	1	0	1	1	1	0	1	0
1	0	1	0	1	1	0	1	0	0
1	0	1	0	1	0	1	1	1	0
1	0	1	0	0	1	1	1	9	2
1	0	0	1	1	1	1	0	0	1
1	0	0	1	1	1	0	1	0	0
1	0	0	1	1	0	1	1	1	0
1	0	0	1	0	1	1	1	1	1
1	0	0	0	1	1	1	1	11	14
0	1	1	1	1	1	0	0	2	0

0	1	1	1	1	0	1	0	0	0
0	1	1	1	1	0	0	1	1	0
0	1	1	1	0	1	1	0	1	0
0	1	1	1	0	1	0	1	2	0
0	1	1	1	0	0	1	1	0	0
0	1	1	0	1	1	1	0	1	0
0	1	1	0	1	1	0	1	0	0
0	1	1	0	1	0	1	1	0	0
0	1	1	0	0	1	1	1	4	0
0	1	0	1	1	1	1	0	0	0
0	1	0	1	1	1	0	1	0	0
0	1	0	1	1	0	1	1	0	0
0	1	0	1	0	1	1	1	1	0
0	1	0	0	1	1	1	1	4	0
0	0	1	1	1	1	1	0	6	4
0	0	1	1	1	1	0	1	14	7
0	0	1	1	1	0	1	1	9	8
0	0	1	1	0	1	1	1	49	14
0	0	1	0	1	1	1	1	46	1
0	0	0	1	1	1	1	1	14	12
1	1	1	1	1	1	0	0	0	0
1	1	1	1	1	0	1	0	0	0
1	1	1	1	1	0	0	1	0	0
1	1	1	1	0	1	1	0	4	0
1	1	1	1	0	1	0	1	5	0
1	1	1	1	0	0	1	1	1	0
1	1	1	0	1	1	1	0	1	0
1	1	1	0	1	1	0	1	0	0
1	1	1	0	1	0	1	1	0	0
1	1	1	0	0	1	1	1	0	0
1	1	0	1	1	1	1	0	1	0
1	1	0	1	1	1	0	1	0	0
1	1	0	1	1	0	1	1	0	0
1	1	0	1	0	1	1	1	0	0
1	1	0	0	1	1	1	1	1	0
1	0	1	1	1	1	1	0	5	1
1	0	1	1	1	1	0	1	6	0
1	0	1	1	1	0	1	1	4	3
1	0	1	1	0	1	1	1	21	8
1	0	1	0	1	1	1	1	17	3
1	0	0	1	1	1	1	1	5	3
0	1	1	1	1	1	1	0	0	0
0	1	1	1	1	1	0	1	2	0
0	1	1	1	1	0	1	1	3	0
0	1	1	1	0	1	1	1	16	0
0	1	1	0	1	1	1	1	1	0
0	1	0	1	1	1	1	1	1	0
0	0	1	1	1	1	1	1	127	216
1	1	1	1	1	1	1	0	1	0
1	1	1	1	1	1	0	1	7	0
1	1	1	1	1	0	1	1	1	0

1	1	1	1	0	1	1	1	43	0
1	1	1	0	1	1	1	1	2	0
1	1	0	1	1	1	1	1	5	0
1	0	1	1	1	1	1	1	119	134
0	1	1	1	1	1	1	1	22	0

Note: The left part of the table illustrates the species combinations for the occurrence (1) or absence (0) of identified indels in CNV analyses; specific duplications and deletions of the species combination from CNV analysis based on using the reference genome of *A. gregorii* were denoted in the right part of the table. DIG = *A. digitata*; GRA = *A. grandidieri*; GRE = *A. gregorii*; MAD = *A. madagascariensis*; PER = *A. perrieri*; RUB = *A. rubrostipa*; SUA = *A. suarezensis*; ZA = *A. za*.

Supplementary Table 4 | The results of *Dsuite* for baobabs based on different reference genomes and outgroup species.

Ref. *A. digitata*

outgroup	P1	P2	P3	*D* statistic	*Z*-score	*p*-value	*f*4-ratio
B. ceiba	**GRE**	**GRA**	**DIG**	**0.0405652**	**13.6579**	**2.30E-16**	**0.0112061**
B. ceiba	GRA	MAD	DIG	0.00703494	2.09291	0.0363571	0.00130205
B. ceiba	GRA	PER	DIG	0.00157696	0.485029	0.627656	0.000293005
B. ceiba	RUB	GRA	DIG	0.00213514	0.668323	0.503927	0.000439454
B. ceiba	GRA	SUA	DIG	0.0103899	2.99477	0.00274648	0.00103768
B. ceiba	GRA	ZA	DIG	0.00420317	1.26492	0.2059	0.000781053
B. ceiba	**GRE**	**MAD**	**DIG**	**0.0455257**	**16.1142**	**2.30E-16**	**0.0124003**
B. ceiba	**GRE**	**PER**	**DIG**	**0.0417858**	**14.8704**	**2.30E-16**	**0.0114213**
B. ceiba	**GRE**	**RUB**	**DIG**	**0.0379956**	**12.3688**	**2.30E-16**	**0.0106507**
B. ceiba	**GRE**	**SUA**	**DIG**	**0.0441722**	**17.3214**	**2.30E-16**	**0.0121188**
B. ceiba	**GRE**	**ZA**	**DIG**	**0.0433199**	**15.6285**	**2.30E-16**	**0.0118356**
B. ceiba	PER	MAD	DIG	0.00851671	2.42577	0.015276	0.000838774
B. ceiba	RUB	MAD	DIG	0.00901914	2.72116	0.00650529	0.00176166
B. ceiba	SUA	MAD	DIG	0.00172307	0.476387	0.633799	0.00031567
B. ceiba	ZA	MAD	DIG	0.00502376	1.32518	0.185111	0.000421549
B. ceiba	RUB	PER	DIG	0.00425153	1.3126	0.189317	0.000835734
B. ceiba	PER	SUA	DIG	0.00373884	1.12485	0.260652	0.000686196
B. ceiba	PER	ZA	DIG	0.00502762	1.31642	0.188034	0.000494927
B. ceiba	RUB	SUA	DIG	0.00673364	2.15784	0.0309401	0.00137943
B. ceiba	RUB	ZA	DIG	0.00671898	2.30128	0.0213756	0.00131749
B. ceiba	ZA	SUA	DIG	0.00185916	0.544134	0.58635	0.000341483
B. ceiba	MAD	GRA	GRE	0.00250703	0.67003	0.502839	0.000346625
B. ceiba	PER	GRA	GRE	0.00158234	0.424983	0.670849	0.000219965
B. ceiba	GRA	RUB	GRE	0.00487072	1.16346	0.244644	0.000749969
B. ceiba	**GRA**	**SUA**	**GRE**	**0.0191033**	**4.44224**	**8.90E-06**	**0.00133683**
B. ceiba	ZA	GRA	GRE	0.00157842	0.433691	0.664513	0.000219393
B. ceiba	MAD	PER	GRA	0.00820343	1.74254	0.0814141	0.00131016

B. ceiba	**GRA**	**MAD**	**RUB**	**0.0661992**	**10.8762**	**2.30E-16**	**0.0206096**
B. ceiba	GRA	SUA	MAD	0.0142315	2.44503	0.0144839	0.00270943
B. ceiba	MAD	ZA	GRA	0.00476402	1.00634	0.314253	0.000641145
B. ceiba	**GRA**	**PER**	**RUB**	**0.0657586**	**11.5692**	**2.30E-16**	**0.0205521**
B. ceiba	**GRA**	**SUA**	**PER**	**0.0191309**	**3.19501**	**0.00139824**	**0.00340285**
B. ceiba	ZA	PER	GRA	0.00418049	1.00221	0.31624	0.000667976
B. ceiba	SUA	GRA	RUB	0.0133683	2.15086	0.0314875	0.00186235
B. ceiba	GRA	ZA	RUB	0.0675374	10.727	2.30E-16	0.0211189
B. ceiba	**GRA**	**SUA**	**ZA**	**0.0179656**	**3.09793**	**0.0019488**	**0.00347328**
B. ceiba	MAD	PER	GRE	0.00112981	0.317528	0.750843	7.90E-05
B. ceiba	MAD	RUB	GRE	0.0068885	1.88131	0.0599299	0.00100909
B. ceiba	**MAD**	**SUA**	**GRE**	**0.0119901**	**3.49573**	**0.00047277**	**0.00164354**
B. ceiba	MAD	ZA	GRE	0.0018934	0.566478	0.571069	0.000112395
B. ceiba	PER	RUB	GRE	0.00571648	1.58101	0.113876	0.000842072
B. ceiba	**PER**	**SUA**	**GRE**	**0.0113644**	**3.51744**	**0.000435731**	**0.00156242**
B. ceiba	PER	ZA	GRE	0.00064072	0.159294	0.873437	4.49E-05
B. ceiba	RUB	SUA	GRE	0.00363529	0.911024	0.362283	0.000558901
B. ceiba	ZA	RUB	GRE	0.00501851	1.43854	0.150282	0.000739254
B. ceiba	**ZA**	**SUA**	**GRE**	**0.0109588**	**3.26439**	**0.001097**	**0.00150794**
B. ceiba	PER	MAD	RUB	0.00230384	0.432514	0.665368	0.000354187
B. ceiba	MAD	PER	SUA	0.0134178	2.89948	0.00373777	0.00211857
B. ceiba	**MAD**	**ZA**	**PER**	**0.020078**	**6.54808**	**5.83E-11**	**0.0106686**
B. ceiba	**SUA**	**MAD**	**RUB**	**0.0717342**	**12.3532**	**2.30E-16**	**0.0220796**
B. ceiba	MAD	ZA	RUB	0.000679512	0.19309	0.846889	8.80E-05
B. ceiba	MAD	ZA	SUA	0.009836	2.07708	0.0377943	0.00130984
B. ceiba	**SUA**	**PER**	**RUB**	**0.0719317**	**14.3774**	**2.30E-16**	**0.0221635**
B. ceiba	PER	ZA	RUB	0.0027787	0.481021	0.630502	0.000427181
B. ceiba	ZA	PER	SUA	0.00517796	1.09198	0.274843	0.000817575
B. ceiba	**SUA**	**ZA**	**RUB**	**0.0735574**	**12.9694**	**2.30E-16**	**0.0227064**
GRE	MAD	PER	GRA	0.000404625	0.0729562	0.941841	7.89E-05
**GRE**	**GRA**	**MAD**	**RUB**	**0.0620133**	**6.98902**	**2.77E-12**	**0.0218369**

GRE	GRA	SUA	MAD	0.00364224	0.580366	0.561668	0.000815792
GRE	ZA	MAD	GRA	0.00534993	1.07604	0.28191	0.000862476
**GRE**	**GRA**	**PER**	**RUB**	**0.0604082**	**7.75016**	**9.18E-15**	**0.0215481**
GRE	GRA	SUA	PER	0.00593082	0.830852	0.406057	0.00124654
GRE	ZA	PER	GRA	0.00402777	0.648571	0.516616	0.00079042
GRE	SUA	GRA	RUB	0.0157215	2.26938	0.023245	0.00269059
**GRE**	**GRA**	**ZA**	**RUB**	**0.0651431**	**7.16539**	**7.76E-13**	**0.0232321**
GRE	GRA	SUA	ZA	0.00429744	0.636339	0.524556	0.000974209
GRE	PER	MAD	RUB	0.00242711	0.316408	0.751693	0.000459012
GRE	MAD	PER	SUA	0.00105226	0.165326	0.868687	0.000202072
**GRE**	**MAD**	**ZA**	**PER**	**0.0210819**	**4.69818**	**2.62E-06**	**0.0113967**
**GRE**	**SUA**	**MAD**	**RUB**	**0.0684681**	**8.77453**	**2.30E-16**	**0.0239238**
GRE	MAD	ZA	RUB	0.004145	1.00769	0.313602	0.000649577
GRE	ZA	MAD	SUA	0.00391903	0.757079	0.449003	0.000621162
**GRE**	**SUA**	**PER**	**RUB**	**0.0682169**	**10.5705**	**2.30E-16**	**0.0241228**
GRE	PER	ZA	RUB	0.00566459	0.780477	0.43511	0.0010806
GRE	ZA	PER	SUA	0.00284955	0.474256	0.635317	0.000549821
**GRE**	**SUA**	**ZA**	**RUB**	**0.0721829**	**9.61906**	**2.30E-16**	**0.0255503**

Ref. *A. grandidieri*

**outgroup**	**P1**	**P2**	**P3**	***D*statistic**	***Z*-score**	***p*-value**	***f*4-ratio**
B. ceiba	**DIG**	**GRE**	**GRA**	**0.0525873**	**12.771**	**2.30E-16**	**0.0219962**
B. ceiba	**GRA**	**MAD**	**DIG**	**0.446542**	**72.0278**	**2.30E-16**	**0.084651**
B. ceiba	**GRA**	**PER**	**DIG**	**0.447481**	**78.5106**	**2.30E-16**	**0.0860868**
B. ceiba	**DIG**	**RUB**	**GRA**	**0.40123**	**117.809**	**2.30E-16**	**0.185854**
B. ceiba	**GRA**	**SUA**	**DIG**	**0.450319**	**69.8648**	**2.30E-16**	**0.0469422**
B. ceiba	**GRA**	**ZA**	**DIG**	**0.449598**	**73.2293**	**2.30E-16**	**0.086367**
B. ceiba	**DIG**	**GRE**	**MAD**	**0.0448239**	**13.9038**	**2.30E-16**	**0.0170031**
B. ceiba	**DIG**	**GRE**	**PER**	**0.0429516**	**12.7216**	**2.30E-16**	**0.0152571**
B. ceiba	**DIG**	**GRE**	**RUB**	**0.0452137**	**19.1663**	**2.30E-16**	**0.0138599**
B. ceiba	**DIG**	**GRE**	**SUA**	**0.0491216**	**13.587**	**2.30E-16**	**0.016284**
B. ceiba	**DIG**	**GRE**	**ZA**	**0.0417999**	**13.1762**	**2.30E-16**	**0.0160276**
B. ceiba	MAD	PER	DIG	0.0048411	1.67693	0.093556	0.000415841
B. ceiba	**MAD**	**RUB**	**DIG**	**0.0343383**	**8.39263**	**2.30E-16**	**0.00611871**
B. ceiba	**SUA**	**MAD**	**DIG**	**0.235997**	**57.8637**	**2.30E-16**	**0.040605**

B. ceiba	**MAD**	**ZA**	**DIG**	**0.013029**	**4.64797**	**3.35E-06**	**0.000947137**
B. ceiba	**PER**	**RUB**	**DIG**	**0.0304045**	**8.37447**	**2.30E-16**	**0.0054666**
B. ceiba	**SUA**	**PER**	**DIG**	**0.239113**	**65.0836**	**2.30E-16**	**0.0415746**
B. ceiba	PER	ZA	DIG	0.00619007	1.65236	0.0984608	0.000531836
B. ceiba	**SUA**	**RUB**	**DIG**	**0.241015**	**74.0817**	**2.30E-16**	**0.0464499**
B. ceiba	**ZA**	**RUB**	**DIG**	**0.0278709**	**7.68593**	**1.52E-14**	**0.00499654**
B. ceiba	**SUA**	**ZA**	**DIG**	**0.241622**	**57.0587**	**2.30E-16**	**0.0419899**
B. ceiba	**GRA**	**MAD**	**GRE**	**0.426046**	**73.7222**	**2.30E-16**	**0.0663168**
B. ceiba	**GRA**	**PER**	**GRE**	**0.425305**	**76.0926**	**2.30E-16**	**0.0669869**
B. ceiba	**GRE**	**RUB**	**GRA**	**0.364971**	**97.7299**	**2.30E-16**	**0.167821**
B. ceiba	**GRA**	**SUA**	**GRE**	**0.433802**	**85.5217**	**2.30E-16**	**0.036437**
B. ceiba	**GRA**	**ZA**	**GRE**	**0.426052**	**72.6492**	**2.30E-16**	**0.0670745**
B. ceiba	**PER**	**MAD**	**GRA**	**0.0191375**	**4.34494**	**1.39E-05**	**0.00371688**
B. ceiba	**RUB**	**MAD**	**GRA**	**0.105193**	**24.615**	**2.30E-16**	**0.0426657**
B. ceiba	**GRA**	**SUA**	**MAD**	**0.457779**	**62.8075**	**2.30E-16**	**0.0925832**
B. ceiba	**ZA**	**MAD**	**GRA**	**0.0203857**	**5.19635**	**2.03E-07**	**0.00334077**
B. ceiba	**RUB**	**PER**	**GRA**	**0.0950925**	**19.2835**	**2.30E-16**	**0.0387007**
B. ceiba	**GRA**	**SUA**	**PER**	**0.471743**	**63.1098**	**2.30E-16**	**0.0893435**
B. ceiba	PER	ZA	GRA	0.00130009	0.29345	0.769178	0.000251302
B. ceiba	**GRA**	**SUA**	**RUB**	**0.44429**	**59.2326**	**2.30E-16**	**0.0675448**
B. ceiba	**RUB**	**ZA**	**GRA**	**0.0954647**	**23.9269**	**2.30E-16**	**0.0387525**
B. ceiba	**GRA**	**SUA**	**ZA**	**0.468924**	**58.5907**	**2.30E-16**	**0.09526**
B. ceiba	MAD	PER	GRE	0.000922902	0.306737	0.759044	6.39E-05
B. ceiba	**MAD**	**RUB**	**GRE**	**0.0352713**	**8.71216**	**2.30E-16**	**0.00516344**
B. ceiba	**SUA**	**MAD**	**GRE**	**0.224857**	**55.2861**	**2.30E-16**	**0.0317655**
B. ceiba	MAD	ZA	GRE	0.00337893	1.13382	0.256871	0.000197337
B. ceiba	**PER**	**RUB**	**GRE**	**0.0336522**	**8.00459**	**1.20E-15**	**0.00495541**
B. ceiba	**SUA**	**PER**	**GRE**	**0.22574**	**55.7653**	**2.30E-16**	**0.0321571**
B. ceiba	PER	ZA	GRE	0.00181172	0.542246	0.587649	0.000125223
B. ceiba	**SUA**	**RUB**	**GRE**	**0.23225**	**62.3948**	**2.30E-16**	**0.0367535**
B. ceiba	**ZA**	**RUB**	**GRE**	**0.0329499**	**8.81823**	**2.30E-16**	**0.0048456**
B. ceiba	**SUA**	**ZA**	**GRE**	**0.226594**	**54.1426**	**2.30E-16**	**0.032254**
B. ceiba	PER	MAD	RUB	0.00464911	0.97677	0.328683	0.000678259
B. ceiba	PER	MAD	SUA	0.000697086	0.158648	0.873946	0.000106596
B. ceiba	**MAD**	**ZA**	**PER**	**0.0216808**	**5.67178**	**1.41E-08**	**0.0111412**

B. ceiba	**RUB**	**MAD**	**SUA**	**0.101184**	**27.0421**	**2.30E-16**	**0.0315109**
B. ceiba	MAD	ZA	RUB	0.00388405	1.17015	0.241943	0.000476312
B. ceiba	ZA	MAD	SUA	0.00367699	0.744525	0.456559	0.000474017
B. ceiba	**RUB**	**PER**	**SUA**	**0.100123**	**22.4857**	**2.30E-16**	**0.0313644**
B. ceiba	PER	ZA	RUB	0.007655	2.2141	0.0268217	0.00111749
B. ceiba	ZA	PER	SUA	0.00227275	0.595369	0.551597	0.000346442
B. ceiba	**RUB**	**ZA**	**SUA**	**0.0984147**	**23.6463**	**2.30E-16**	**0.0307128**
**GRE**	**PER**	**MAD**	**GRA**	**0.0281993**	**4.33701**	**1.44E-05**	**0.00648098**
**GRE**	**RUB**	**MAD**	**GRA**	**0.095246**	**8.41405**	**2.30E-16**	**0.0418262**
**GRE**	**GRA**	**SUA**	**MAD**	**0.0626173**	**12.0608**	**2.30E-16**	**0.0143643**
**GRE**	**ZA**	**MAD**	**GRA**	**0.0336531**	**4.91443**	**8.90E-07**	**0.00636666**
**GRE**	**RUB**	**PER**	**GRA**	**0.0743444**	**6.7572**	**1.41E-11**	**0.0328034**
**GRE**	**GRA**	**SUA**	**PER**	**0.0810204**	**14.4298**	**2.30E-16**	**0.0171263**
GRE	ZA	PER	GRA	0.000369896	0.0542991	0.956697	8.42E-05
**GRE**	**GRA**	**SUA**	**RUB**	**0.0557112**	**11.3412**	**2.30E-16**	**0.00977331**
**GRE**	**RUB**	**ZA**	**GRA**	**0.0762329**	**6.97111**	**3.14E-12**	**0.0334701**
**GRE**	**GRA**	**SUA**	**ZA**	**0.0784379**	**13.3034**	**2.30E-16**	**0.0178597**
GRE	PER	MAD	RUB	0.00773791	1.1813	0.237483	0.001497
GRE	PER	MAD	SUA	0.0151175	2.36176	0.0181885	0.00305682
**GRE**	**MAD**	**ZA**	**PER**	**0.0241912**	**3.68343**	**0.000230113**	**0.0131096**
**GRE**	**RUB**	**MAD**	**SUA**	**0.0964124**	**8.64391**	**2.30E-16**	**0.0369837**
GRE	MAD	ZA	RUB	0.0051845	1.1765	0.239397	0.000828255
**GRE**	**ZA**	**MAD**	**SUA**	**0.018171**	**3.0904**	**0.00199884**	**0.00302035**
**GRE**	**RUB**	**PER**	**SUA**	**0.0843272**	**7.78409**	**7.02E-15**	**0.0326048**
GRE	PER	ZA	RUB	0.0112157	1.95984	0.050014	0.00218209
GRE	PER	ZA	SUA	0.000493064	0.0735368	0.941379	9.92E-05
**GRE**	**RUB**	**ZA**	**SUA**	**0.084406**	**7.71782**	**1.18E-14**	**0.0324927**

Ref. *A. gregorii*

**outgroup**	**P1**	**P2**	**P3**	***D*statistic**	***Z*-score**	***p*-value**	***f*4-ratio**
B. ceiba	**DIG**	**GRA**	**GRE**	**0.0991939**	**23.4421**	**2.30E-16**	**0.0275912**
B. ceiba	GRA	MAD	DIG	0.000126307	0.0267187	0.978684	2.14E-05
B. ceiba	GRA	PER	DIG	0.00294966	0.806464	0.419976	0.000502679

B. ceiba	GRA	RUB	DIG	0.000835257	0.275221	0.783147	0.000157188
B. ceiba	GRA	SUA	DIG	0.00970202	2.50013	0.0124147	0.00085385
B. ceiba	GRA	ZA	DIG	0.0052605	1.13021	0.258386	0.000895827
B. ceiba	**DIG**	**MAD**	**GRE**	**0.109029**	**33.06**	**2.30E-16**	**0.0299203**
B. ceiba	**DIG**	**PER**	**GRE**	**0.100594**	**30.5866**	**2.30E-16**	**0.0276176**
B. ceiba	**DIG**	**RUB**	**GRE**	**0.096811**	**24.5137**	**2.30E-16**	**0.0273376**
B. ceiba	**DIG**	**SUA**	**GRE**	**0.104042**	**25.4401**	**2.30E-16**	**0.0287376**
B. ceiba	**DIG**	**ZA**	**GRE**	**0.100355**	**27.792**	**2.30E-16**	**0.0275158**
B. ceiba	MAD	PER	DIG	0.00521917	1.11356	0.265467	0.000458258
B. ceiba	MAD	RUB	DIG	0.000956524	0.303593	0.761438	0.000171025
B. ceiba	MAD	SUA	DIG	0.00572606	1.22861	0.219218	0.000960347
B. ceiba	**MAD**	**ZA**	**DIG**	**0.0104898**	**3.33629**	**0.000849033**	**0.000783829**
B. ceiba	RUB	PER	DIG	0.00214214	0.717221	0.473238	0.000386766
B. ceiba	PER	SUA	DIG	0.00250598	0.647074	0.517584	0.000422977
B. ceiba	PER	ZA	DIG	0.00373157	0.836877	0.402662	0.000328952
B. ceiba	RUB	SUA	DIG	0.00403921	1.11365	0.26543	0.000757778
B. ceiba	RUB	ZA	DIG	0.00397256	1.20313	0.228927	0.000714768
B. ceiba	SUA	ZA	DIG	1.76E-05	0.0036297	0.997104	2.97E-06
B. ceiba	**GRA**	**MAD**	**GRE**	**0.0131162**	**3.2912**	**0.000997604**	**0.00239861**
B. ceiba	GRA	PER	GRE	0.00171186	0.373674	0.708647	0.000313338
B. ceiba	GRA	RUB	GRE	0.000156505	0.0381099	0.9696	3.18E-05
B. ceiba	**GRA**	**SUA**	**GRE**	**0.0144014**	**4.83176**	**1.35E-06**	**0.00136925**
B. ceiba	GRA	ZA	GRE	0.000556556	0.114295	0.909004	0.000101731
B. ceiba	MAD	PER	GRA	0.0083826	1.792	0.0731326	0.00134704
B. ceiba	**GRA**	**MAD**	**RUB**	**0.0662023**	**9.9036**	**2.30E-16**	**0.0205827**
B. ceiba	GRA	SUA	MAD	0.0132131	2.62032	0.00878484	0.00253519
B. ceiba	MAD	ZA	GRA	0.00657062	1.69973	0.0891814	0.000891865
B. ceiba	**GRA**	**PER**	**RUB**	**0.0660685**	**11.0269**	**2.30E-16**	**0.0206165**
B. ceiba	**GRA**	**SUA**	**PER**	**0.0165275**	**3.54051**	**0.000399356**	**0.00297385**

B. ceiba	ZA	PER	GRA	0.00262405	0.616593	0.537504	0.000422243
B. ceiba	SUA	GRA	RUB	0.0144035	2.6113	0.00901994	0.00202398
B. ceiba	**GRA**	**ZA**	**RUB**	**0.0691551**	**9.75002**	**2.30E-16**	**0.0215891**
B. ceiba	**GRA**	**SUA**	**ZA**	**0.0155839**	**3.05316**	**0.00226442**	**0.00304555**
B. ceiba	**PER**	**MAD**	**GRE**	**0.0202695**	**4.24086**	**2.23E-05**	**0.00190564**
B. ceiba	**RUB**	**MAD**	**GRE**	**0.0128154**	**3.2132**	**0.00131263**	**0.00247832**
B. ceiba	SUA	MAD	GRE	0.00649651	1.62526	0.104107	0.00117489
B. ceiba	**ZA**	**MAD**	**GRE**	**0.0260645**	**5.45997**	**4.76E-08**	**0.00208321**
B. ceiba	RUB	PER	GRE	0.00160605	0.428552	0.66825	0.000311212
B. ceiba	PER	SUA	GRE	0.00571283	1.35926	0.174065	0.0010302
B. ceiba	ZA	PER	GRE	0.00253686	0.517821	0.604583	0.000236678
B. ceiba	RUB	SUA	GRE	0.00614033	1.53843	0.123944	0.00123933
B. ceiba	RUB	ZA	GRE	0.000678592	0.158538	0.874033	0.000131341
B. ceiba	ZA	SUA	GRE	0.00675408	1.42758	0.153414	0.00121798
B. ceiba	PER	MAD	RUB	0.000665004	0.124996	0.900526	0.000103102
B. ceiba	MAD	PER	SUA	0.0131043	2.9761	0.00291941	0.00208518
B. ceiba	**MAD**	**ZA**	**PER**	**0.0204664**	**5.48647**	**4.10E-08**	**0.010896**
B. ceiba	**SUA**	**MAD**	**RUB**	**0.0730338**	**9.47033**	**2.30E-16**	**0.0224035**
B. ceiba	MAD	ZA	RUB	0.00455871	1.49126	0.135894	0.000595662
B. ceiba	MAD	ZA	SUA	0.0095291	2.29038	0.0219994	0.00128049
B. ceiba	**SUA**	**PER**	**RUB**	**0.0727669**	**10.4924**	**2.30E-16**	**0.0223862**
B. ceiba	PER	ZA	RUB	0.00592496	1.0732	0.283183	0.00091961
B. ceiba	ZA	PER	SUA	0.00419758	1.08171	0.279382	0.000669364
B. ceiba	**SUA**	**ZA**	**RUB**	**0.0760235**	**9.52797**	**2.30E-16**	**0.0233976**
**GRE**	**MAD**	**PER**	**GRA**	**0.0233809**	**3.22584**	**0.00125602**	**0.00473346**
**GRE**	**GRA**	**MAD**	**RUB**	**0.0597602**	**6.8454**	**7.63E-12**	**0.0213955**
GRE	GRA	SUA	MAD	0.0123308	2.39631	0.016561	0.00285068
**GRE**	**MAD**	**ZA**	**GRA**	**0.0201396**	**5.38898**	**7.09E-08**	**0.003378**
**GRE**	**GRA**	**PER**	**RUB**	**0.0716879**	**8.42374**	**2.30E-16**	**0.0259344**
**GRE**	**GRA**	**SUA**	**PER**	**0.0137303**	**3.73561**	**0.000187262**	**0.00302015**
GRE	ZA	PER	GRA	0.00488314	0.765143	0.444187	0.00099577
GRE	SUA	GRA	RUB	0.00168712	0.3338	0.738531	0.000302851

**GRE**	**GRA**	**ZA**	**RUB**	**0.078029**	**8.03906**	**9.05E-16**	**0.0282594**
GRE	GRA	SUA	ZA	0.0140155	2.65328	0.00797146	0.00330536
GRE	MAD	PER	RUB	0.0154646	2.58979	0.00960354	0.00305583
**GRE**	**MAD**	**PER**	**SUA**	**0.0250795**	**4.49699**	**6.89E-06**	**0.00500434**
**GRE**	**MAD**	**ZA**	**PER**	**0.0390998**	**7.16563**	**7.74E-13**	**0.0212366**
**GRE**	**SUA**	**MAD**	**RUB**	**0.0558738**	**6.05486**	**1.41E-09**	**0.019919**
**GRE**	**MAD**	**ZA**	**RUB**	**0.030573**	**6.24524**	**4.23E-10**	**0.0050049**
**GRE**	**MAD**	**ZA**	**SUA**	**0.0248161**	**6.50333**	**7.86E-11**	**0.00409627**
**GRE**	**SUA**	**PER**	**RUB**	**0.0696042**	**7.84582**	**4.30E-15**	**0.0250871**
GRE	PER	ZA	RUB	0.0113346	1.50603	0.13206	0.00226058
GRE	ZA	PER	SUA	0.00199378	0.449365	0.653169	0.000400393
**GRE**	**SUA**	**ZA**	**RUB**	**0.0748915**	**7.37232**	**1.68E-13**	**0.0270187**

Ref. *A. madagascariensis*

outgroup	P1	P2	P3	*D*statistic	*Z*-score	*p*-value	*f*4-ratio
B. ceiba	**DIG**	**GRE**	**GRA**	**0.0460475**	**13.325**	**2.30E-16**	**0.0144863**
B. ceiba	**MAD**	**GRA**	**DIG**	**0.393849**	**58.6531**	**2.30E-16**	**0.072795**
B. ceiba	**PER**	**GRA**	**DIG**	**0.229013**	**67.902**	**2.30E-16**	**0.040005**
B. ceiba	**GRA**	**RUB**	**DIG**	**0.0131459**	**3.37299**	**0.000743563**	**0.00247492**
B. ceiba	GRA	SUA	DIG	0.0100238	2.9861	0.00282562	0.000856255
B. ceiba	**ZA**	**GRA**	**DIG**	**0.272471**	**72.399**	**2.30E-16**	**0.0483226**
B. ceiba	**DIG**	**GRE**	**MAD**	**0.0530659**	**15.0175**	**2.30E-16**	**0.025768**
B. ceiba	**DIG**	**GRE**	**PER**	**0.0465391**	**12.5556**	**2.30E-16**	**0.0178045**
B. ceiba	**DIG**	**GRE**	**RUB**	**0.0478306**	**12.3153**	**2.30E-16**	**0.014642**
B. ceiba	**DIG**	**GRE**	**SUA**	**0.0475888**	**14.111**	**2.30E-16**	**0.0147991**
B. ceiba	**DIG**	**GRE**	**ZA**	**0.0457053**	**12.1902**	**2.30E-16**	**0.0198701**
B. ceiba	**MAD**	**PER**	**DIG**	**0.354701**	**56.7398**	**2.30E-16**	**0.0348636**
B. ceiba	**MAD**	**RUB**	**DIG**	**0.385958**	**59.5921**	**2.30E-16**	**0.0749441**
B. ceiba	**MAD**	**SUA**	**DIG**	**0.402104**	**62.0854**	**2.30E-16**	**0.0740111**
B. ceiba	**MAD**	**ZA**	**DIG**	**0.317653**	**51.9181**	**2.30E-16**	**0.0262301**
B. ceiba	**PER**	**RUB**	**DIG**	**0.228025**	**56.633**	**2.30E-16**	**0.0422241**
B. ceiba	**PER**	**SUA**	**DIG**	**0.237106**	**77.3699**	**2.30E-16**	**0.0411537**
B. ceiba	**ZA**	**PER**	**DIG**	**0.0977999**	**27.3018**	**2.30E-16**	**0.00889396**
B. ceiba	SUA	RUB	DIG	0.00750253	2.03354	0.0419978	0.00141014
B. ceiba	**ZA**	**RUB**	**DIG**	**0.269917**	**70.6723**	**2.30E-16**	**0.050512**
B. ceiba	**ZA**	**SUA**	**DIG**	**0.280916**	**76.8302**	**2.30E-16**	**0.0495026**
B. ceiba	**MAD**	**GRA**	**GRE**	**0.375676**	**50.0838**	**2.30E-16**	**0.0569735**
B. ceiba	**PER**	**GRA**	**GRE**	**0.223136**	**41.7132**	**2.30E-16**	**0.0318881**
B. ceiba	**GRA**	**RUB**	**GRE**	**0.0171052**	**4.39665**	**1.10E-05**	**0.00263789**
B. ceiba	**GRA**	**SUA**	**GRE**	**0.0147101**	**5.93385**	**2.96E-09**	**0.00100868**
B. ceiba	**ZA**	**GRA**	**GRE**	**0.267082**	**47.8725**	**2.30E-16**	**0.0388016**
B. ceiba	**MAD**	**PER**	**GRA**	**0.362693**	**61.4225**	**2.30E-16**	**0.0587607**
B. ceiba	**RUB**	**GRA**	**MAD**	**0.0390627**	**5.26846**	**1.38E-07**	**0.0213253**
B. ceiba	GRA	SUA	MAD	0.0118381	2.49801	0.0124892	0.00278085
B. ceiba	**MAD**	**ZA**	**GRA**	**0.319178**	**52.8192**	**2.30E-16**	**0.0431188**

B. ceiba	**RUB**	**GRA**	**PER**	**0.0388828**	**4.63895**	**3.50E-06**	**0.0159796**
B. ceiba	**GRA**	**SUA**	**PER**	**0.0133389**	**3.26158**	**0.00110794**	**0.00238722**
B. ceiba	**ZA**	**PER**	**GRA**	**0.107816**	**23.5491**	**2.30E-16**	**0.0164193**
B. ceiba	**SUA**	**GRA**	**RUB**	**0.0164677**	**3.00107**	**0.00269031**	**0.00215033**
B. ceiba	**RUB**	**GRA**	**ZA**	**0.0367723**	**5.03093**	**4.88E-07**	**0.0175876**
B. ceiba	GRA	SUA	ZA	0.0131197	2.82343	0.00475135	0.00273167
B. ceiba	**MAD**	**PER**	**GRE**	**0.33489**	**56.7852**	**2.30E-16**	**0.0264543**
B. ceiba	**MAD**	**RUB**	**GRE**	**0.371513**	**49.9785**	**2.30E-16**	**0.0592631**
B. ceiba	**MAD**	**SUA**	**GRE**	**0.385346**	**52.6463**	**2.30E-16**	**0.0581696**
B. ceiba	**MAD**	**ZA**	**GRE**	**0.292099**	**53.0414**	**2.30E-16**	**0.0193171**
B. ceiba	**PER**	**RUB**	**GRE**	**0.226086**	**38.5293**	**2.30E-16**	**0.034298**
B. ceiba	**PER**	**SUA**	**GRE**	**0.232584**	**44.836**	**2.30E-16**	**0.0330081**
B. ceiba	**ZA**	**PER**	**GRE**	**0.100431**	**22.7711**	**2.30E-16**	**0.0073376**
B. ceiba	SUA	RUB	GRE	0.00991949	2.18579	0.0288311	0.00152725
B. ceiba	**ZA**	**RUB**	**GRE**	**0.267847**	**45.6318**	**2.30E-16**	**0.0411295**
B. ceiba	**ZA**	**SUA**	**GRE**	**0.277553**	**50.8431**	**2.30E-16**	**0.0400202**
B. ceiba	**MAD**	**PER**	**RUB**	**0.352594**	**44.5436**	**2.30E-16**	**0.054273**
B. ceiba	**MAD**	**PER**	**SUA**	**0.367546**	**70.1468**	**2.30E-16**	**0.0588432**
B. ceiba	**PER**	**ZA**	**MAD**	**0.248878**	**30.8061**	**2.30E-16**	**0.243544**
B. ceiba	**RUB**	**SUA**	**MAD**	**0.0436781**	**5.6389**	**1.71E-08**	**0.0238081**
B. ceiba	**MAD**	**ZA**	**RUB**	**0.314471**	**42.7417**	**2.30E-16**	**0.0405708**
B. ceiba	**MAD**	**ZA**	**SUA**	**0.323586**	**55.5287**	**2.30E-16**	**0.0431986**
B. ceiba	**RUB**	**SUA**	**PER**	**0.0442339**	**5.21504**	**1.84E-07**	**0.0181742**
B. ceiba	**ZA**	**PER**	**RUB**	**0.0990081**	**25.4615**	**2.30E-16**	**0.0143328**
B. ceiba	**ZA**	**PER**	**SUA**	**0.10911**	**26.3778**	**2.30E-16**	**0.0164269**
B. ceiba	**RUB**	**SUA**	**ZA**	**0.042103**	**5.5099**	**3.59E-08**	**0.0201206**
**GRE**	**MAD**	**PER**	**GRA**	**0.0521805**	**7.58653**	**3.29E-14**	**0.0101892**
GRE	GRA	MAD	RUB	0.00620279	0.696336	0.486219	0.00218977
GRE	SUA	GRA	MAD	0.00551696	0.816821	0.414031	0.00145384
**GRE**	**MAD**	**ZA**	**GRA**	**0.0448926**	**8.30675**	**2.30E-16**	**0.00707286**
GRE	GRA	PER	RUB	0.0257141	2.9605	0.0030714	0.00915628
GRE	SUA	GRA	PER	0.00345448	0.628657	0.529573	0.000777126
GRE	ZA	PER	GRA	0.0146329	1.90174	0.0572054	0.00285491
GRE	SUA	GRA	RUB	0.0142913	2.7762	0.00549991	0.00251204
GRE	GRA	ZA	RUB	0.029178	2.96065	0.00306995	0.0104151
GRE	SUA	GRA	ZA	0.004043	0.572554	0.566947	0.00101494
**GRE**	**MAD**	**PER**	**RUB**	**0.0421068**	**6.54463**	**5.96E-11**	**0.00794973**
**GRE**	**MAD**	**PER**	**SUA**	**0.0548262**	**9.52128**	**2.30E-16**	**0.0104621**
**GRE**	**MAD**	**ZA**	**PER**	**0.0952527**	**12.7234**	**2.30E-16**	**0.0461263**
GRE	SUA	MAD	RUB	0.0109575	1.14236	0.253305	0.00385518
**GRE**	**MAD**	**ZA**	**RUB**	**0.0574174**	**12.3438**	**2.30E-16**	**0.00876448**
**GRE**	**MAD**	**ZA**	**SUA**	**0.0506954**	**11.0029**	**2.30E-16**	**0.00778116**
**GRE**	**SUA**	**PER**	**RUB**	**0.031597**	**3.34672**	**0.000817752**	**0.0112096**
GRE	PER	ZA	RUB	0.00412617	0.592973	0.553199	0.000781785
GRE	ZA	PER	SUA	0.012476	2.3767	0.0174684	0.00237345
**GRE**	**SUA**	**ZA**	**RUB**	**0.0337282**	**3.32815**	**0.000874241**	**0.0119789**

Ref. *A. perrieri*

**outgroup**	**P1**	**P2**	**P3**	***D*** **statistic**	***Z*-score**	***p*-value**	***f4*-ratio**

B. ceiba	**DIG**	**GRE**	**GRA**	**0.0434313**	**8.92317**	**2.30E-16**	**0.0138083**
B. ceiba	**MAD**	**GRA**	**DIG**	**0.232105**	**50.1793**	**2.30E-16**	**0.04118**
B. ceiba	**PER**	**GRA**	**DIG**	**0.412756**	**53.1391**	**2.30E-16**	**0.0786355**
B. ceiba	**GRA**	**RUB**	**DIG**	**0.0129298**	**3.5366**	**0.000405315**	**0.00246978**
B. ceiba	GRA	SUA	DIG	0.00708608	1.66366	0.0961814	0.000622081
B. ceiba	**ZA**	**GRA**	**DIG**	**0.226281**	**47.1587**	**2.30E-16**	**0.0402643**
B. ceiba	**DIG**	**GRE**	**MAD**	**0.0457231**	**10.134**	**2.30E-16**	**0.0191146**
B. ceiba	**DIG**	**GRE**	**PER**	**0.0495451**	**10.0459**	**2.30E-16**	**0.023227**
B. ceiba	**DIG**	**GRE**	**RUB**	**0.0440012**	**10.2426**	**2.30E-16**	**0.0136168**
B. ceiba	**DIG**	**GRE**	**SUA**	**0.0450862**	**9.31275**	**2.30E-16**	**0.0141645**
B. ceiba	**DIG**	**GRE**	**ZA**	**0.0420666**	**9.2068**	**2.30E-16**	**0.0177717**
B. ceiba	**PER**	**MAD**	**DIG**	**0.388466**	**48.3404**	**2.30E-16**	**0.0396058**
B. ceiba	**MAD**	**RUB**	**DIG**	**0.232932**	**48.65**	**2.30E-16**	**0.0434666**
B. ceiba	**MAD**	**SUA**	**DIG**	**0.238838**	**57.1726**	**2.30E-16**	**0.0420017**
B. ceiba	**MAD**	**ZA**	**DIG**	**0.0121462**	**3.87386**	**0.000107127**	**0.00093404**
B. ceiba	**PER**	**RUB**	**DIG**	**0.404814**	**58.0656**	**2.30E-16**	**0.0807979**
B. ceiba	**PER**	**SUA**	**DIG**	**0.420948**	**57.2095**	**2.30E-16**	**0.0795236**
B. ceiba	**PER**	**ZA**	**DIG**	**0.395389**	**51.3464**	**2.30E-16**	**0.0407274**
B. ceiba	SUA	RUB	DIG	0.00893791	2.06394	0.0390231	0.00170486
B. ceiba	**ZA**	**RUB**	**DIG**	**0.226834**	**53.9965**	**2.30E-16**	**0.0425081**
B. ceiba	**ZA**	**SUA**	**DIG**	**0.233319**	**50.7877**	**2.30E-16**	**0.0411049**
B. ceiba	**MAD**	**GRA**	**GRE**	**0.224095**	**54.1489**	**2.30E-16**	**0.0325086**
B. ceiba	**PER**	**GRA**	**GRE**	**0.393733**	**55.3411**	**2.30E-16**	**0.0614191**
B. ceiba	**GRA**	**RUB**	**GRE**	**0.016428**	**4.04705**	**5.19E-05**	**0.00255922**
B. ceiba	GRA	SUA	GRE	0.0112946	2.22409	0.0261421	0.000795585
B. ceiba	**ZA**	**GRA**	**GRE**	**0.221753**	**50.8969**	**2.30E-16**	**0.0322365**
B. ceiba	**PER**	**MAD**	**GRA**	**0.393052**	**48.3339**	**2.30E-16**	**0.0657581**
B. ceiba	**RUB**	**GRA**	**MAD**	**0.0275956**	**3.90662**	**9.36E-05**	**0.0126961**
B. ceiba	GRA	SUA	MAD	0.0132414	2.67107	0.00756104	0.00267502
B. ceiba	MAD	ZA	GRA	0.00244184	0.589449	0.55556	0.000313515
B. ceiba	**RUB**	**GRA**	**PER**	**0.0399141**	**5.28584**	**1.25E-07**	**0.0206758**
B. ceiba	**GRA**	**SUA**	**PER**	**0.01823**	**3.44027**	**0.000581139**	**0.00411355**
B. ceiba	**PER**	**ZA**	**GRA**	**0.395025**	**52.4802**	**2.30E-16**	**0.0665663**
B. ceiba	SUA	GRA	RUB	0.0171493	2.67102	0.00756212	0.00230672
B. ceiba	**RUB**	**GRA**	**ZA**	**0.0278722**	**3.6139**	**0.00030162**	**0.0129751**
B. ceiba	GRA	SUA	ZA	0.0164675	2.88405	0.00392593	0.00336017

B. ceiba	**PER**	**MAD**	**GRE**	**0.369267**	**42.4533**	**2.30E-16**	**0.0303018**
B. ceiba	**MAD**	**RUB**	**GRE**	**0.228043**	**51.6727**	**2.30E-16**	**0.0348515**
B. ceiba	**MAD**	**SUA**	**GRE**	**0.232605**	**55.2755**	**2.30E-16**	**0.0334612**
B. ceiba	MAD	ZA	GRE	0.00385817	1.26577	0.205594	0.000237477
B. ceiba	**PER**	**RUB**	**GRE**	**0.389082**	**60.1259**	**2.30E-16**	**0.0636467**
B. ceiba	**PER**	**SUA**	**GRE**	**0.40345**	**56.0388**	**2.30E-16**	**0.062429**
B. ceiba	**PER**	**ZA**	**GRE**	**0.371642**	**42.0381**	**2.30E-16**	**0.0307505**
B. ceiba	SUA	RUB	GRE	0.0102595	1.98147	0.0475387	0.00159658
B. ceiba	**ZA**	**RUB**	**GRE**	**0.225504**	**50.2133**	**2.30E-16**	**0.0345549**
B. ceiba	**ZA**	**SUA**	**GRE**	**0.230737**	**52.5084**	**2.30E-16**	**0.0332005**
B. ceiba	**PER**	**MAD**	**RUB**	**0.397145**	**43.1559**	**2.30E-16**	**0.0636156**
B. ceiba	**PER**	**MAD**	**SUA**	**0.394344**	**46.3387**	**2.30E-16**	**0.0652279**
B. ceiba	MAD	ZA	PER	0.000878913	0.226057	0.821157	0.000553232
B. ceiba	**RUB**	**SUA**	**MAD**	**0.033676**	**4.9493**	**7.45E-07**	**0.0154752**
B. ceiba	MAD	ZA	RUB	0.00105418	0.338103	0.735286	0.000129822
B. ceiba	MAD	ZA	SUA	0.00586507	1.28331	0.199383	0.000743452
B. ceiba	**RUB**	**SUA**	**PER**	**0.047804**	**6.81887**	**9.18E-12**	**0.0247334**
B. ceiba	**PER**	**ZA**	**RUB**	**0.397018**	**45.9315**	**2.30E-16**	**0.0642138**
B. ceiba	**PER**	**ZA**	**SUA**	**0.398796**	**49.6942**	**2.30E-16**	**0.0665764**
B. ceiba	**RUB**	**SUA**	**ZA**	**0.035702**	**4.88735**	**1.02E-06**	**0.016598**
**GRE**	**PER**	**MAD**	**GRA**	**0.0632955**	**7.43652**	**1.03E-13**	**0.0122456**
**GRE**	**GRA**	**MAD**	**RUB**	**0.0345204**	**3.35844**	**0.000783849**	**0.0123464**
GRE	GRA	SUA	MAD	0.000363594	0.0557618	0.955532	8.93E-05
GRE	ZA	MAD	GRA	0.0063932	0.910811	0.362395	0.00102748
GRE	PER	GRA	RUB	0.00581179	0.646222	0.518135	0.00207094
GRE	SUA	GRA	PER	0.00535118	0.893368	0.37166	0.00132692
**GRE**	**PER**	**ZA**	**GRA**	**0.0587746**	**8.50356**	**2.30E-16**	**0.0115616**
GRE	SUA	GRA	RUB	0.0126791	1.93776	0.0526523	0.00225446
**GRE**	**GRA**	**ZA**	**RUB**	**0.0372314**	**3.41807**	**0.000630669**	**0.0134289**
GRE	GRA	SUA	ZA	0.000602229	0.0931379	0.925794	0.000149261
**GRE**	**PER**	**MAD**	**RUB**	**0.0762557**	**10.3096**	**2.30E-16**	**0.0143574**
**GRE**	**PER**	**MAD**	**SUA**	**0.0730898**	**11.0107**	**2.30E-16**	**0.0137906**

GRE	ZA	MAD	PER	0.000326829	0.0435511	0.965262	0.000192575
**GRE**	**SUA**	**MAD**	**RUB**	**0.0387904**	**3.44749**	**0.000565811**	**0.0137959**
GRE	MAD	ZA	RUB	0.00728022	1.63173	0.102736	0.00114651
GRE	ZA	MAD	SUA	0.00440613	0.989342	0.322496	0.000691655
GRE	PER	SUA	RUB	0.000526332	0.0546681	0.956403	0.000187527
**GRE**	**PER**	**ZA**	**RUB**	**0.081463**	**8.77392**	**2.30E-16**	**0.0156597**
**GRE**	**PER**	**ZA**	**SUA**	**0.0707799**	**12.0739**	**2.30E-16**	**0.0135578**
**GRE**	**SUA**	**ZA**	**RUB**	**0.0421123**	**3.79504**	**0.000147618**	**0.0150977**

Ref. *A. rubrostipa*

outgroup	P1	P2	P3	*D* statistic	*Z*-score	*p*-value	*f*4-ratio
B. ceiba	**DIG**	**GRE**	**GRA**	**0.0444819**	**11.1859**	**2.30E-16**	**0.0139528**
B. ceiba	**MAD**	**GRA**	**DIG**	**0.0177707**	**4.88018**	**1.06E-06**	**0.00298773**
B. ceiba	**PER**	**GRA**	**DIG**	**0.0119581**	**3.70988**	**0.00020736**	**0.00202507**
B. ceiba	**DIG**	**GRA**	**RUB**	**0.404442**	**107.54**	**2.30E-16**	**0.180373**
B. ceiba	**GRA**	**SUA**	**DIG**	**0.0151613**	**7.17008**	**7.50E-13**	**0.00130022**
B. ceiba	ZA	GRA	DIG	0.0116961	2.93136	0.00337478	0.0019776
B. ceiba	**DIG**	**GRE**	**MAD**	**0.0442574**	**15.2502**	**2.30E-16**	**0.0167636**
B. ceiba	**DIG**	**GRE**	**PER**	**0.0422959**	**15.0224**	**2.30E-16**	**0.0150248**
B. ceiba	**DIG**	**GRE**	**RUB**	**0.0548103**	**19.2599**	**2.30E-16**	**0.0223093**
B. ceiba	**DIG**	**GRE**	**SUA**	**0.0459254**	**13.1416**	**2.30E-16**	**0.0142665**
B. ceiba	**DIG**	**GRE**	**ZA**	**0.0417231**	**13.5667**	**2.30E-16**	**0.0159865**
B. ceiba	MAD	PER	DIG	0.0100514	2.68585	0.00723458	0.000863892
B. ceiba	**RUB**	**MAD**	**DIG**	**0.441655**	**81.2644**	**2.30E-16**	**0.0880742**
B. ceiba	**MAD**	**SUA**	**DIG**	**0.0265176**	**7.63642**	**2.23E-14**	**0.00441814**
B. ceiba	**MAD**	**ZA**	**DIG**	**0.0130779**	**4.15359**	**3.27E-05**	**0.000952384**
B. ceiba	**DIG**	**PER**	**RUB**	**0.436211**	**96.8192**	**2.30E-16**	**0.196778**
B. ceiba	**PER**	**SUA**	**DIG**	**0.0204909**	**6.88133**	**5.93E-12**	**0.00343636**
B. ceiba	PER	ZA	DIG	0.000552641	0.116389	0.907344	4.76E-05
B. ceiba	**DIG**	**SUA**	**RUB**	**0.405056**	**101.671**	**2.30E-16**	**0.178611**
B. ceiba	**DIG**	**ZA**	**RUB**	**0.438772**	**84.3911**	**2.30E-16**	**0.197665**
B. ceiba	**ZA**	**SUA**	**DIG**	**0.0202793**	**5.25392**	**1.49E-07**	**0.00339516**
B. ceiba	**MAD**	**GRA**	**GRE**	**0.0167563**	**4.97076**	**6.67E-07**	**0.00230628**
B. ceiba	**PER**	**GRA**	**GRE**	**0.0138739**	**3.67451**	**0.000238304**	**0.00191912**
B. ceiba	**GRE**	**GRA**	**RUB**	**0.363553**	**114.36**	**2.30E-16**	**0.161624**
B. ceiba	**GRA**	**SUA**	**GRE**	**0.0207645**	**6.17984**	**6.42E-10**	**0.00143414**

B. ceiba	**ZA**	**GRA**	**GRE**	**0.0146674**	**3.87834**	**0.000105172**	**0.00202733**
B. ceiba	MAD	PER	GRA	0.01025	2.56673	0.0102664	0.00155419
B. ceiba	**GRA**	**MAD**	**RUB**	**0.0671329**	**10.9763**	**2.30E-16**	**0.0252938**
B. ceiba	**GRA**	**SUA**	**MAD**	**0.0169084**	**3.63556**	**0.000277376**	**0.00309689**
B. ceiba	MAD	ZA	GRA	0.00847538	2.09713	0.0359822	0.00108017
B. ceiba	**GRA**	**PER**	**RUB**	**0.0545034**	**9.95628**	**2.30E-16**	**0.020526**
B. ceiba	**GRA**	**SUA**	**PER**	**0.0210994**	**4.45222**	**8.50E-06**	**0.00359331**
B. ceiba	ZA	PER	GRA	0.00271361	0.669639	0.503088	0.000411548
B. ceiba	SUA	GRA	RUB	0.0120211	2.42322	0.0153834	0.0021089
B. ceiba	**GRA**	**ZA**	**RUB**	**0.0568346**	**8.59024**	**2.30E-16**	**0.0214002**
B. ceiba	**GRA**	**SUA**	**ZA**	**0.018582**	**3.51302**	**0.000443041**	**0.00345168**
B. ceiba	MAD	PER	GRE	0.00615906	1.33002	0.183512	0.000425354
B. ceiba	**GRE**	**MAD**	**RUB**	**0.405097**	**93.3209**	**2.30E-16**	**0.18271**
B. ceiba	**MAD**	**SUA**	**GRE**	**0.0277731**	**9.07948**	**2.30E-16**	**0.00378439**
B. ceiba	MAD	ZA	GRE	0.00339318	0.811495	0.417082	0.000198148
B. ceiba	**GRE**	**PER**	**RUB**	**0.397296**	**90.9404**	**2.30E-16**	**0.178808**
B. ceiba	**PER**	**SUA**	**GRE**	**0.0244798**	**7.67116**	**1.70E-14**	**0.00334848**
B. ceiba	ZA	PER	GRE	0.00265663	0.492128	0.622629	0.000183906
B. ceiba	**GRE**	**SUA**	**RUB**	**0.364457**	**100.981**	**2.30E-16**	**0.160029**
B. ceiba	**GRE**	**ZA**	**RUB**	**0.398492**	**78.7361**	**2.30E-16**	**0.179521**
B. ceiba	**ZA**	**SUA**	**GRE**	**0.0257699**	**6.80008**	**1.05E-11**	**0.00352326**
B. ceiba	**PER**	**MAD**	**RUB**	**0.0230214**	**4.46677**	**7.94E-06**	**0.00434064**
B. ceiba	**MAD**	**PER**	**SUA**	**0.0150878**	**4.2656**	**1.99E-05**	**0.00225781**
B. ceiba	**MAD**	**ZA**	**PER**	**0.0224023**	**5.64121**	**1.69E-08**	**0.0114765**
B. ceiba	**SUA**	**MAD**	**RUB**	**0.0740692**	**11.9352**	**2.30E-16**	**0.0274828**
B. ceiba	**ZA**	**MAD**	**RUB**	**0.0217191**	**4.94516**	**7.61E-07**	**0.00346063**
B. ceiba	MAD	ZA	SUA	0.0118573	2.53224	0.0113337	0.00149291
B. ceiba	**SUA**	**PER**	**RUB**	**0.0612**	**10.8799**	**2.30E-16**	**0.0226649**
B. ceiba	PER	ZA	RUB	0.00449459	0.813605	0.415871	0.000844828
B. ceiba	ZA	PER	SUA	0.00501978	1.40347	0.160475	0.000753406
B. ceiba	**SUA**	**ZA**	**RUB**	**0.0635836**	**9.29016**	**2.30E-16**	**0.0235429**
GRE	PER	MAD	GRA	0.00263615	0.480545	0.63084	0.000517256

**GRE**	**GRA**	**MAD**	**RUB**	**0.0712252**	**7.62884**	**2.37E-14**	**0.0290317**
GRE	GRA	SUA	MAD	0.00293354	0.334146	0.73827	0.000674711
GRE	ZA	MAD	GRA	0.00691422	1.77473	0.0759427	0.00111852
**GRE**	**GRA**	**PER**	**RUB**	**0.0483003**	**5.91782**	**3.26E-09**	**0.0197239**
GRE	GRA	SUA	PER	0.00249592	0.359143	0.719488	0.000536078
GRE	ZA	PER	GRA	0.00191162	0.320924	0.748268	0.000376099
**GRE**	**SUA**	**GRA**	**RUB**	**0.0204282**	**3.20755**	**0.00133873**	**0.00414423**
**GRE**	**GRA**	**ZA**	**RUB**	**0.0579461**	**5.63927**	**1.71E-08**	**0.0237417**
GRE	GRA	SUA	ZA	0.00114003	0.134686	0.89286	0.00026533
**GRE**	**PER**	**MAD**	**RUB**	**0.0338985**	**4.15231**	**3.29E-05**	**0.00744837**
GRE	PER	MAD	SUA	0.00387193	0.687959	0.491479	0.000745551
GRE	MAD	ZA	PER	0.0238507	2.9945	0.00274891	0.0128047
**GRE**	**SUA**	**MAD**	**RUB**	**0.0804923**	**8.57642**	**2.30E-16**	**0.0324408**
**GRE**	**ZA**	**MAD**	**RUB**	**0.0180268**	**3.0824**	**0.00205342**	**0.00326772**
GRE	ZA	MAD	SUA	0.00586069	1.53106	0.125755	0.000928585
**GRE**	**SUA**	**PER**	**RUB**	**0.0589665**	**7.10107**	**1.24E-12**	**0.0238329**
GRE	PER	ZA	RUB	0.0165999	1.72081	0.0852847	0.00363401
GRE	PER	ZA	SUA	0.000416171	0.0854159	0.931931	8.02E-05
**GRE**	**SUA**	**ZA**	**RUB**	**0.0675424**	**6.42103**	**1.35E-10**	**0.0273692**

Ref. *A. suarezensis*

**outgroup**	**P1**	**P2**	**P3**	***D* statistic**	***Z*-score**	***p*-value**	***f*4-ratio**
B. ceiba	**DIG**	**GRE**	**GRA**	**0.0464534**	**12.7157**	**2.30E-16**	**0.0155769**
B. ceiba	**GRA**	**MAD**	**DIG**	**0.240159**	**56.8957**	**2.30E-16**	**0.0418885**
B. ceiba	**GRA**	**PER**	**DIG**	**0.242994**	**57.1678**	**2.30E-16**	**0.0428729**
B. ceiba	**GRA**	**RUB**	**DIG**	**0.248281**	**59.3444**	**2.30E-16**	**0.0481675**
B. ceiba	**SUA**	**GRA**	**DIG**	**0.461545**	**67.7877**	**2.30E-16**	**0.0481389**
B. ceiba	**GRA**	**ZA**	**DIG**	**0.245758**	**57.6977**	**2.30E-16**	**0.0433084**
B. ceiba	**DIG**	**GRE**	**MAD**	**0.0429685**	**11.5126**	**2.30E-16**	**0.0163198**
B. ceiba	**DIG**	**GRE**	**PER**	**0.0406249**	**9.60874**	**2.30E-16**	**0.0144628**
B. ceiba	**DIG**	**GRE**	**RUB**	**0.043028**	**10.0732**	**2.30E-16**	**0.0132165**
B. ceiba	**DIG**	**GRE**	**SUA**	**0.0555765**	**13.9928**	**2.30E-16**	**0.0232303**
B. ceiba	**DIG**	**GRE**	**ZA**	**0.0395481**	**9.14217**	**2.30E-16**	**0.0151895**
B. ceiba	MAD	PER	DIG	0.00494288	1.36271	0.172974	0.000426846
B. ceiba	**MAD**	**RUB**	**DIG**	**0.0366549**	**10.5073**	**2.30E-16**	**0.00654966**
B. ceiba	**SUA**	**MAD**	**DIG**	**0.460877**	**66.0675**	**2.30E-16**	**0.0870546**

B. ceiba	**MAD**	**ZA**	**DIG**	**0.012476**	**3.0791**	**0.00207628**	**0.000912291**
B. ceiba	**PER**	**RUB**	**DIG**	**0.0333192**	**11.2906**	**2.30E-16**	**0.00600703**
B. ceiba	**SUA**	**PER**	**DIG**	**0.462458**	**67.1083**	**2.30E-16**	**0.0885054**
B. ceiba	PER	ZA	DIG	0.00576066	1.50271	0.132913	0.000496584
B. ceiba	**DIG**	**RUB**	**SUA**	**0.401229**	**113.633**	**2.30E-16**	**0.184309**
B. ceiba	**ZA**	**RUB**	**DIG**	**0.0302368**	**11.3397**	**2.30E-16**	**0.00543093**
B. ceiba	**SUA**	**ZA**	**DIG**	**0.464294**	**65.5551**	**2.30E-16**	**0.0887469**
B. ceiba	**GRA**	**MAD**	**GRE**	**0.229063**	**61.9635**	**2.30E-16**	**0.0327097**
B. ceiba	**GRA**	**PER**	**GRE**	**0.230338**	**62.9997**	**2.30E-16**	**0.0331913**
B. ceiba	**GRA**	**RUB**	**GRE**	**0.24041**	**71.6878**	**2.30E-16**	**0.0381894**
B. ceiba	**SUA**	**GRA**	**GRE**	**0.435133**	**69.2797**	**2.30E-16**	**0.0363753**
B. ceiba	**GRA**	**ZA**	**GRE**	**0.230292**	**56.7251**	**2.30E-16**	**0.0331503**
B. ceiba	PER	MAD	GRA	0.00516837	1.21222	0.225427	0.000800935
B. ceiba	**RUB**	**MAD**	**GRA**	**0.0818008**	**21.1554**	**2.30E-16**	**0.0260801**
B. ceiba	**SUA**	**GRA**	**MAD**	**0.466435**	**63.6367**	**2.30E-16**	**0.0940037**
B. ceiba	ZA	MAD	GRA	0.00485178	1.35419	0.175675	0.000636167
B. ceiba	**RUB**	**PER**	**GRA**	**0.0785685**	**17.9128**	**2.30E-16**	**0.0251836**
B. ceiba	**SUA**	**GRA**	**PER**	**0.474841**	**63.1017**	**2.30E-16**	**0.0896306**
B. ceiba	PER	ZA	GRA	0.000324505	0.081418	0.93511	5.02E-05
B. ceiba	**SUA**	**GRA**	**RUB**	**0.485075**	**73.2174**	**2.30E-16**	**0.074188**
B. ceiba	**RUB**	**ZA**	**GRA**	**0.0789373**	**20.6322**	**2.30E-16**	**0.0252417**
B. ceiba	**SUA**	**GRA**	**ZA**	**0.475429**	**64.6316**	**2.30E-16**	**0.0962825**
B. ceiba	MAD	PER	GRE	0.000482492	0.125849	0.899852	3.34E-05
B. ceiba	**MAD**	**RUB**	**GRE**	**0.0386068**	**9.59998**	**2.30E-16**	**0.00564864**
B. ceiba	**SUA**	**MAD**	**GRE**	**0.434495**	**71.778**	**2.30E-16**	**0.0671439**
B. ceiba	MAD	ZA	GRE	0.0017719	0.43082	0.666599	0.000103488
B. ceiba	**PER**	**RUB**	**GRE**	**0.0374802**	**10.2618**	**2.30E-16**	**0.00551549**
B. ceiba	**SUA**	**PER**	**GRE**	**0.435174**	**75.3783**	**2.30E-16**	**0.0679253**
B. ceiba	PER	ZA	GRE	0.00111735	0.252122	0.800947	7.71E-05
B. ceiba	**GRE**	**RUB**	**SUA**	**0.361359**	**89.1108**	**2.30E-16**	**0.16519**
B. ceiba	**ZA**	**RUB**	**GRE**	**0.0366359**	**8.46426**	**2.30E-16**	**0.00537916**
B. ceiba	**SUA**	**ZA**	**GRE**	**0.435371**	**68.4488**	**2.30E-16**	**0.0679339**
B. ceiba	PER	MAD	RUB	0.00493671	0.879463	0.37915	0.000720445
B. ceiba	PER	MAD	SUA	0.0125583	2.88492	0.00391516	0.00241346
B. ceiba	**MAD**	**ZA**	**PER**	**0.0211708**	**4.54028**	**5.62E-06**	**0.0109195**
B. ceiba	**RUB**	**MAD**	**SUA**	**0.118254**	**32.4515**	**2.30E-16**	**0.0473151**
B. ceiba	MAD	ZA	RUB	0.00496906	1.28837	0.197618	0.000611512
B. ceiba	ZA	MAD	SUA	0.0154229	2.97056	0.00297257	0.00250344
B. ceiba	**RUB**	**PER**	**SUA**	**0.110952**	**26.6874**	**2.30E-16**	**0.0445402**
B. ceiba	PER	ZA	RUB	0.0079668	1.41918	0.155846	0.00116098

B. ceiba	ZA	PER	SUA	0.000833581	0.171105	0.864141	0.000159493
B. ceiba	**RUB**	**ZA**	**SUA**	**0.110692**	**25.4464**	**2.30E-16**	**0.0442832**
GRE	PER	MAD	GRA	0.0154111	2.69656	0.00700605	0.00318017
**GRE**	**RUB**	**MAD**	**GRA**	**0.0893267**	**10.4451**	**2.30E-16**	**0.0352385**
**GRE**	**SUA**	**GRA**	**MAD**	**0.0672206**	**8.34678**	**2.30E-16**	**0.0153112**
**GRE**	**ZA**	**MAD**	**GRA**	**0.0183867**	**3.14962**	**0.00163485**	**0.00312747**
**GRE**	**RUB**	**PER**	**GRA**	**0.0768451**	**8.18405**	**2.74E-16**	**0.0306048**
**GRE**	**SUA**	**GRA**	**PER**	**0.0851198**	**12.0227**	**2.30E-16**	**0.0178849**
GRE	PER	ZA	GRA	0.000372055	0.0538919	0.957021	7.68E-05
**GRE**	**SUA**	**GRA**	**RUB**	**0.0857781**	**18.6426**	**2.30E-16**	**0.0149795**
**GRE**	**RUB**	**ZA**	**GRA**	**0.0778612**	**10.0382**	**2.30E-16**	**0.0308444**
**GRE**	**SUA**	**GRA**	**ZA**	**0.0830617**	**10.1608**	**2.30E-16**	**0.0188405**
GRE	PER	MAD	RUB	0.00978674	1.22874	0.21917	0.00189643
**GRE**	**PER**	**MAD**	**SUA**	**0.0279024**	**4.207**	**2.59E-05**	**0.00627455**
**GRE**	**MAD**	**ZA**	**PER**	**0.0207681**	**3.1**	**0.00193523**	**0.0112915**
**GRE**	**RUB**	**MAD**	**SUA**	**0.101087**	**11.7854**	**2.30E-16**	**0.043408**
GRE	MAD	ZA	RUB	0.00675922	1.8894	0.0588387	0.00108143
**GRE**	**ZA**	**MAD**	**SUA**	**0.0296846**	**6.04107**	**1.53E-09**	**0.00548415**
**GRE**	**RUB**	**PER**	**SUA**	**0.0811773**	**8.67219**	**2.30E-16**	**0.0350457**
GRE	PER	ZA	RUB	0.0140246	1.76526	0.0775207	0.00273724
GRE	PER	ZA	SUA	0.00342043	0.505967	0.61288	0.000761979
**GRE**	**RUB**	**ZA**	**SUA**	**0.0824568**	**9.48292**	**2.30E-16**	**0.0353744**

Ref. *A. za*

**outgroup**	**P1**	**P2**	**P3**	***D* statistic**	***Z*-score**	***p*-value**	***f*4-ratio**
B. ceiba	**DIG**	**GRE**	**GRA**	**0.0437011**	**10.259**	**2.30E-16**	**0.01378**
B. ceiba	**MAD**	**GRA**	**DIG**	**0.277443**	**68.5424**	**2.30E-16**	**0.0491022**
B. ceiba	**PER**	**GRA**	**DIG**	**0.230827**	**66.0276**	**2.30E-16**	**0.0403988**
B. ceiba	**GRA**	**RUB**	**DIG**	**0.0103096**	**3.53933**	**0.000401143**	**0.00194073**
B. ceiba	GRA	SUA	DIG	0.00675129	2.59776	0.00938332	0.000579178
B. ceiba	**ZA**	**GRA**	**DIG**	**0.383458**	**68.9539**	**2.30E-16**	**0.0706521**
B. ceiba	**DIG**	**GRE**	**MAD**	**0.0474852**	**14.5322**	**2.30E-16**	**0.0205409**
B. ceiba	**DIG**	**GRE**	**PER**	**0.0456531**	**12.186**	**2.30E-16**	**0.01754**
B. ceiba	**DIG**	**GRE**	**RUB**	**0.0463087**	**13.5189**	**2.30E-16**	**0.0142031**
B. ceiba	**DIG**	**GRE**	**SUA**	**0.0456032**	**10.4797**	**2.30E-16**	**0.0142074**
B. ceiba	**DIG**	**GRE**	**ZA**	**0.0485676**	**13.1806**	**2.30E-16**	**0.0240235**

B. ceiba	**MAD**	**PER**	**DIG**	**0.102625**	**22.2075**	**2.30E-16**	**0.00927041**
B. ceiba	**MAD**	**RUB**	**DIG**	**0.273107**	**82.0347**	**2.30E-16**	**0.0508621**
B. ceiba	**MAD**	**SUA**	**DIG**	**0.285246**	**71.4923**	**2.30E-16**	**0.0500098**
B. ceiba	**ZA**	**MAD**	**DIG**	**0.288024**	**60.9055**	**2.30E-16**	**0.0230611**
B. ceiba	**PER**	**RUB**	**DIG**	**0.227966**	**85.4568**	**2.30E-16**	**0.0421414**
B. ceiba	**PER**	**SUA**	**DIG**	**0.238378**	**68.6558**	**2.30E-16**	**0.0412997**
B. ceiba	**ZA**	**PER**	**DIG**	**0.334108**	**54.7106**	**2.30E-16**	**0.0322734**
B. ceiba	SUA	RUB	DIG	0.00640812	2.20242	0.0276359	0.00120147
B. ceiba	**ZA**	**RUB**	**DIG**	**0.374987**	**76.8855**	**2.30E-16**	**0.0724155**
B. ceiba	**ZA**	**SUA**	**DIG**	**0.391908**	**76.1959**	**2.30E-16**	**0.0715958**
B. ceiba	**MAD**	**GRA**	**GRE**	**0.265619**	**47.4359**	**2.30E-16**	**0.0385637**
B. ceiba	**PER**	**GRA**	**GRE**	**0.222054**	**42.3006**	**2.30E-16**	**0.0318357**
B. ceiba	**GRA**	**RUB**	**GRE**	**0.0159896**	**3.75664**	**0.000172211**	**0.00246265**
B. ceiba	GRA	SUA	GRE	0.00989074	2.08834	0.0367669	0.000680567
B. ceiba	**ZA**	**GRA**	**GRE**	**0.367027**	**56.4119**	**2.30E-16**	**0.0553717**
B. ceiba	**MAD**	**PER**	**GRA**	**0.106561**	**23.7232**	**2.30E-16**	**0.0161813**
B. ceiba	**RUB**	**GRA**	**MAD**	**0.0344687**	**4.52948**	**5.91E-06**	**0.0163409**
B. ceiba	**GRA**	**SUA**	**MAD**	**0.0150637**	**4.85923**	**1.18E-06**	**0.00311611**
B. ceiba	**ZA**	**MAD**	**GRA**	**0.296411**	**69.779**	**2.30E-16**	**0.0390121**
B. ceiba	**RUB**	**GRA**	**PER**	**0.0371219**	**4.60174**	**4.19E-06**	**0.01528**
B. ceiba	**GRA**	**SUA**	**PER**	**0.016433**	**6.64241**	**3.09E-11**	**0.00294723**
B. ceiba	**ZA**	**PER**	**GRA**	**0.343713**	**70.8934**	**2.30E-16**	**0.0549668**
B. ceiba	**SUA**	**GRA**	**RUB**	**0.0163236**	**3.65402**	**0.000258168**	**0.00214324**
B. ceiba	**RUB**	**GRA**	**ZA**	**0.040522**	**5.52329**	**3.33E-08**	**0.0225259**
B. ceiba	**GRA**	**SUA**	**ZA**	**0.0175958**	**4.58531**	**4.53E-06**	**0.00422829**
B. ceiba	**MAD**	**PER**	**GRE**	**0.0968226**	**19.2406**	**2.30E-16**	**0.00706531**
B. ceiba	**MAD**	**RUB**	**GRE**	**0.265606**	**55.1546**	**2.30E-16**	**0.040685**
B. ceiba	**MAD**	**SUA**	**GRE**	**0.274273**	**47.7189**	**2.30E-16**	**0.0394629**
B. ceiba	**ZA**	**MAD**	**GRE**	**0.276796**	**54.9121**	**2.30E-16**	**0.0178401**
B. ceiba	**PER**	**RUB**	**GRE**	**0.224304**	**46.1563**	**2.30E-16**	**0.0340179**
B. ceiba	**PER**	**SUA**	**GRE**	**0.230841**	**43.5732**	**2.30E-16**	**0.0327567**
B. ceiba	**ZA**	**PER**	**GRE**	**0.319968**	**50.5659**	**2.30E-16**	**0.0248612**
B. ceiba	SUA	RUB	GRE	0.0103389	2.21045	0.0270739	0.00158729
B. ceiba	**ZA**	**RUB**	**GRE**	**0.362458**	**66.3679**	**2.30E-16**	**0.0574889**
B. ceiba	**ZA**	**SUA**	**GRE**	**0.376777**	**58.2**	**2.30E-16**	**0.0563395**

B. ceiba	**MAD**	**PER**	**RUB**	**0.0975494**	**18.5325**	**2.30E-16**	**0.0140833**
B. ceiba	**MAD**	**PER**	**SUA**	**0.109939**	**23.72**	**2.30E-16**	**0.0164788**
B. ceiba	**PER**	**MAD**	**ZA**	**0.245369**	**45.6553**	**2.30E-16**	**0.252363**
B. ceiba	**RUB**	**SUA**	**MAD**	**0.0405218**	**5.44711**	**5.12E-08**	**0.0191709**
B. ceiba	**ZA**	**MAD**	**RUB**	**0.29798**	**64.503**	**2.30E-16**	**0.0375119**
B. ceiba	**ZA**	**MAD**	**SUA**	**0.297034**	**63.1407**	**2.30E-16**	**0.0386641**
B. ceiba	**RUB**	**SUA**	**PER**	**0.0442027**	**5.58302**	**2.36E-08**	**0.0181586**
B. ceiba	**ZA**	**PER**	**RUB**	**0.337946**	**58.5834**	**2.30E-16**	**0.0514057**
B. ceiba	**ZA**	**PER**	**SUA**	**0.347178**	**77.7762**	**2.30E-16**	**0.0548925**
B. ceiba	**RUB**	**SUA**	**ZA**	**0.0473838**	**6.3471**	**2.19E-10**	**0.0262803**
GRE	MAD	PER	GRA	0.0116976	1.71564	0.0862272	0.00227503
GRE	GRA	MAD	RUB	0.02566	2.35688	0.0184293	0.00909727
GRE	SUA	GRA	MAD	0.000612541	0.0843416	0.932785	0.00015312
**GRE**	**ZA**	**MAD**	**GRA**	**0.0511826**	**7.75149**	**9.08E-15**	**0.0078671**
**GRE**	**GRA**	**PER**	**RUB**	**0.0268077**	**3.00032**	**0.002697**	**0.00956153**
GRE	SUA	GRA	PER	0.00153807	0.246241	0.805495	0.000347713
**GRE**	**ZA**	**PER**	**GRA**	**0.0543293**	**8.70147**	**2.30E-16**	**0.0105159**
GRE	SUA	GRA	RUB	0.0114425	1.92604	0.0540996	0.0020203
GRE	GRA	ZA	RUB	0.00894013	0.82889	0.407166	0.00317114
GRE	SUA	GRA	ZA	0.00461679	0.627022	0.530645	0.00123894
GRE	MAD	PER	RUB	0.00242625	0.28802	0.773331	0.000458333
GRE	MAD	PER	SUA	0.010615	1.65264	0.0984049	0.00201623
**GRE**	**ZA**	**MAD**	**PER**	**0.0539225**	**6.8429**	**7.76E-12**	**0.0258363**
GRE	SUA	MAD	RUB	0.028094	2.46848	0.0135689	0.00992758
**GRE**	**ZA**	**MAD**	**RUB**	**0.0428447**	**11.527**	**2.30E-16**	**0.00640572**
**GRE**	**ZA**	**MAD**	**SUA**	**0.0537895**	**8.87283**	**2.30E-16**	**0.00804903**
**GRE**	**SUA**	**PER**	**RUB**	**0.0310392**	**3.08285**	**0.00205028**	**0.0110383**
**GRE**	**ZA**	**PER**	**RUB**	**0.0383003**	**4.79585**	**1.62E-06**	**0.00722169**
**GRE**	**ZA**	**PER**	**SUA**	**0.0553772**	**10.0232**	**2.30E-16**	**0.0104465**
GRE	SUA	ZA	RUB	0.0116931	1.07166	0.283872	0.0041384

Note: Data with *Z*-scores exceeding 3 are shown in bold and indicate the *D*-statistics was significantly larger than zero. DIG = *A. digitata*; GRA = *A. grandidieri*; GRE = *A. gregorii*; MAD = *A. madagascariensis*; PER = *A. perrieri*; RUB = *A. rubrostipa*; SUA = *A. suarezensis*; ZA = *A. za*.

Supplementary Table 5 | The results of QuIBL analysis for different triplet tree topologies.

Topology	*mixprop 1*	*mixprop 2*	*Lambda -2Dist*	*lambda1 -Dist*	*BIC2Dist*	*BIC1Dist*	*count*
((GRE, RUB), DIG)	0.994	0.006	0.009	0.013	-2381.502	-2211.954	329
((DIG, RUB), GRE)	0.997	0.003	0.011	0.013	-2434.979	-2294.437	347
((DIG, GRE), RUB)	0.984	0.016	0.008	0.011	-2276.419	-2197.836	312
((GRE, GRA), DIG)	0.980	0.020	0.006	0.008	-2725.144	-2622.721	345
((DIG, GRA), GRE)	0.997	0.003	0.009	0.012	-2448.406	-2314.376	336
((DIG, GRE), GRA)	0.967	0.033	0.007	0.010	-2276.786	-2214.820	306
((GRE, MAD), DIG)	0.981	0.019	0.007	0.009	-2604.920	-2540.540	344
((DIG, MAD), GRE)	0.997	0.003	0.009	0.013	-2342.051	-2162.224	324
((DIG, GRE), MAD)	0.985	0.015	0.007	0.010	-2410.789	-2306.055	319
((GRE, PER), DIG)	0.885	0.115	0.005	0.007	-2583.991	-2620.611	329
((DIG, PER), GRE)	0.974	0.026	0.006	0.008	-2445.407	-2326.884	307
((DIG, GRE), PER)	0.947	0.053	0.007	0.011	-2537.062	-2460.125	350
((GRE, SUA), DIG)	0.954	0.046	0.006	0.008	-2590.917	-2575.623	337
((DIG, SUA), GRE)	0.992	0.008	0.007	0.008	-2749.255	-2685.736	353
((DIG, GRE), SUA)	0.960	0.040	0.008	0.012	-2137.528	-2061.581	299
((GRE, ZA), DIG)	0.975	0.025	0.007	0.009	-2522.248	-2449.518	328
((DIG, ZA), GRE)	0.997	0.003	0.012	0.016	-2470.557	-2289.393	365
((DIG, GRE), ZA)	0.977	0.023	0.008	0.010	-2214.445	-2136.412	299
((GRA, PER), RUB)	0.997	0.003	0.009	0.014	-2463.919	-2227.566	341
((GRA, RUB), PER)	0.997	0.003	0.009	0.013	-2255.311	-2050.829	307
((RUB, PER), GRA)	0.997	0.003	0.008	0.021	-2520.746	-1920.019	337
((GRA, MAD), RUB)	0.997	0.003	0.011	0.015	-2282.437	-2122.878	330
((GRA, RUB), MAD)	0.991	0.009	0.009	0.011	-2215.500	-2099.727	303
((RUB, MAD), GRA)	0.997	0.003	0.009	0.015	-2618.381	-2271.699	355
((GRA, ZA), RUB)	0.997	0.003	0.015	0.023	-2220.179	-1941.107	351
((GRA, RUB), ZA)	0.958	0.042	0.006	0.008	-2378.377	-2314.839	303
((RUB, ZA), GRA)	0.997	0.003	0.010	0.016	-2374.829	-2099.487	337
((SUA, MAD), RUB)	0.991	0.009	0.012	0.016	-2193.132	-2079.216	330
((SUA, RUB), MAD)	0.997	0.003	0.012	0.019	-2080.096	-1845.573	311
((RUB, MAD), SUA)	0.997	0.003	0.008	0.018	-2630.926	-2098.631	351
((SUA, PER), RUB)	0.997	0.003	0.009	0.012	-2511.380	-2340.608	344
((SUA, RUB), PER)	0.984	0.016	0.010	0.013	-2218.884	-2093.271	317
((RUB, PER), SUA)	0.916	0.084	0.005	0.008	-2520.673	-2534.081	327
((SUA, ZA), RUB)	0.997	0.003	0.010	0.013	-2373.849	-2228.495	336
((SUA, RUB), ZA)	0.993	0.007	0.008	0.011	-2233.188	-2078.490	300

((RUB, ZA), SUA)	0.997	0.003	0.011	0.027	-2598.483	-1856.473	358

Note: Two alternative topologies of the triplet species tree are indicated: *mixprop1* means the inferred mixing proportions for each distribution while *mixprop2* corresponds to the non-ILS component. *lambda1Dist*, and *lambda2Dist* indicate the scaling factor required to go from substitutions per site to coalescent units for a 2 and 1 distribution model. *BIC2Dist* and *BIC1Dist* mean BIC scores for the 'introgression + ILS' model and 'ILS only' model respectively for model selection. *count* means the total number of trees in that triplet topology. DIG = *A. digitata*; GRA = *A. grandidieri*, GRE = *A. gregorii*, MAD = *A. madagascariensis*, PER = *A. perrieri*, RUB = *A. rubrostipa*, SUA = *A. suarezensis*, ZA = *A. za*.

Supplementary Table 6 | Heterozygosity of each chromosome in diploid baobabs.

Chromosome	GRA	GRE	MAD	PER	RUB	SUA	ZA
Chr01	0.0156	0.0136	0.0143	0.0115	0.0163	0.0053	0.0148
Chr02	0.0101	0.0067	0.0138	0.0111	0.0117	0.0074	0.0127
Chr03	0.0225	0.0161	0.0171	0.0116	0.0120	0.0132	0.0127
Chr04	0.0095	0.0114	0.0173	0.0159	0.0144	0.0092	0.0179
Chr05	0.0163	0.0183	0.0172	0.0173	0.0205	0.0077	0.0170
Chr06	0.0148	0.0122	0.0151	0.0142	0.0165	0.0107	0.0153
Chr07	0.0149	0.0098	0.0183	0.0156	0.0136	0.0098	0.0169
Chr08	0.0106	0.0103	0.0163	0.0054	0.0139	0.0050	0.0152
Chr09	0.0096	0.0089	0.0153	0.0148	0.0152	0.0049	0.0173
Chr10	0.0115	0.0113	0.0149	0.0068	0.0104	0.0081	0.0156
Chr11	0.0141	0.0074	0.0155	0.0121	0.0110	0.0085	0.0164
Chr12	0.0091	0.0094	0.0155	0.0161	0.0116	0.0066	0.0161
Chr13	0.0094	0.0075	0.0183	0.0121	0.0139	0.0093	0.0147
Chr14	0.0114	0.0109	0.0177	0.0132	0.0120	0.0087	0.0150
Chr15	0.0116	0.0094	0.0168	0.0072	0.0166	0.0076	0.0150
Chr16	0.0089	0.0109	0.0197	0.0152	0.0146	0.0100	0.0200
Chr17	0.0089	0.0093	0.0178	0.0146	0.0150	0.0050	0.0165
Chr18	0.0111	0.0086	0.0133	0.0155	0.0125	0.0041	0.0157
Chr19	0.0094	0.0130	0.0186	0.0136	0.0112	0.0060	0.0174
Chr20	0.0119	0.0078	0.0157	0.0165	0.0128	0.0070	0.0152
Chr21	0.0124	0.0097	0.0170	0.0138	0.0122	0.0089	0.0159
Chr22	0.0121	0.0095	0.0172	0.0167	0.0135	0.0052	0.0127
Chr23	0.0103	0.0125	0.0159	0.0169	0.0145	0.0063	0.0173
Chr24	0.0110	0.0078	0.0165	0.0122	0.0124	0.0080	0.0171
Chr25	0.0124	0.0093	0.0175	0.0036	0.0131	0.0090	0.0152
Chr26	0.0121	0.0081	0.0154	0.0131	0.0135	0.0098	0.0160
Chr27	0.0160	0.0109	0.0184	0.0092	0.0164	0.0100	0.0164
Chr28	0.0153	0.0150	0.0166	0.0118	0.0150	0.0084	0.0160
Chr29	0.0120	0.0110	0.0127	0.0162	0.0155	0.0073	0.0158
Chr30	0.0143	0.0116	0.0169	0.0153	0.0166	0.0076	0.0179
Chr31	0.0181	0.0089	0.0184	0.0151	0.0128	0.0063	0.0173
Chr32	0.0089	0.0105	0.0188	0.0119	0.0157	0.0096	0.0156
Chr33	0.0123	0.0152	0.0128	0.0108	0.0134	0.0055	0.0178
Chr34	0.0126	0.0107	0.0193	0.0119	0.0158	0.0096	0.0169
Chr35	0.0125	0.0089	0.0181	0.0123	0.0143	0.0054	0.0146
Chr36	0.0165	0.0098	0.0176	0.0187	0.0156	0.0078	0.0178
Chr37	0.0059	0.0125	0.0163	0.0123	0.0135	0.0088	0.0157
Chr38	0.0162	0.0115	0.0151	0.0190	0.0167	0.0095	0.0178
Chr39	0.0122	0.0104	0.0176	0.0143	0.0118	0.0099	0.0152
Chr40	0.0096	0.0095	0.0191	0.0204	0.0156	0.0139	0.0183
Chr41	0.0122	0.0103	0.0213	0.0158	0.0122	0.0099	0.0160
Chr42	0.0096	0.0112	0.0182	0.0133	0.0176	0.0096	0.0191
Chr43	0.0151	0.0123	0.0162	-	0.0119	0.0072	0.0163
Chr44	0.0058	0.0108	0.0174	-	0.0140	0.0098	0.0183
Mean	0.0122	0.0107	0.0168	0.0134	0.0141	0.0081	0.0162
Global	0.0117	0.0104	0.0169	0.0133	0.0138	0.0082	0.0163

Note: DIG = *A. digitata*; GRA = *A. grandidieri*, GRE = *A. gregorii*, MAD = *A. madagascariensis*, PER = *A. perrieri*, RUB = *A. rubrostipa*, SUA = *A. suarezensis*, ZA = *A. za*.

Supplementary Table 7 | The statistics of runs of homozygosity (ROH) of different length.

Species	Length of ROH < 0.5 Mb	Length of ROH 0.5 - 1 Mb	Length of ROH 1 - 2 Mb	Length of ROH > 2 Mb
A. grandidieri	39.1403	18.8269	16.3953	26.8331
A. gregorii	24.9345	2.0194	1.0458	2.1707
A. madagascariensis	71.5018	25.1311	6.9212	4.4468
A. perrieri	41.638	14.844	21.3405	80.2853
A. rubrostipa	72.1933	9.9985	1.0837	0
A. suarezensis	37.2593	16.5174	21.1491	25.8895
A. za	56.7464	13.2077	2.9114	4.4017

Supplementary Table 8 | Resulting AUC values for the four MaxEnt models. Values shown for current climatic conditions, and as predicted, for the last glacial maximum (LGM, ~ 22,000 years BP) and last interglacial (LIG, ~ 130,000 years BP).

**Species**	**Current - AUC**	**LGM - AUC**	**LIG - AUC**
Adansonia grandidieri	0.989	0.984	0.988
Adansonia madagascariensis	0.951	0.945	0.933
Adansonia perrieri	0.997	0.993	0.996
Adansonia rubrostipa	0.958	0.967	0.967
Adansonia suarezensis	0.997	0.975	0.995
Adansonia za	0.968	0.883	0.893

Supplementary Table 9 | Pearson correlation analysis of environmental factors selected in this study.

	Bio 1	Bio 2	Bio 3	Bio 4	Bio 5	Bio 6	Bio 7	Bio 8	Bio 9	Bio 10	Bio 11	Bio 12	Bio 13	Bio 14	Bio 15	Bio 16	Bio 17	Bio 18	Bio 19
**Bio 1**	1	0.17763	0.2792	-0.41817	0.92399	0.83782	0.09761	0.9656	0.96813	0.97647	0.97993	-0.09085	0.17075	-0.18551	0.32412	0.05542	-0.17178	-0.39853	-0.12627
**Bio 2**	0.17763	1	0.3047	0.32879	0.50936	-0.35538	0.95225	0.22293	-0.01392	0.22422	0.0603	-0.79012	-0.47217	-0.78698	0.63768	-0.58237	-0.78359	-0.70063	-0.76037
**Bio 3**	0.2792	0.3047	1	-0.60838	0.24567	0.24372	0.00291	0.11723	0.29126	0.14157	0.36947	-0.16696	0.26929	-0.52331	0.51856	0.16271	-0.51506	-0.20072	-0.52173
**Bio 4**	-0.41817	0.32879	-0.60838	1	-0.16174	-0.65429	0.54098	-0.1804	-0.53425	-0.22131	-0.58725	-0.44538	-0.79901	0.04959	-0.24585	-0.72395	0.04494	-0.19125	0.03938
**Bio 5**	0.92399	0.50936	0.24567	-0.16174	1	0.58747	0.45663	0.93309	0.82233	0.94117	0.84865	-0.35819	-0.06347	-0.39353	0.4597	-0.19376	-0.38013	-0.59761	-0.32882
**Bio 6**	0.83782	-0.35538	0.24372	-0.65429	0.58747	1	-0.45169	0.7567	0.92549	0.77101	0.90119	0.36075	0.47321	0.21668	-0.00714	0.41856	0.22926	0.01986	0.25865
**Bio 7**	0.09761	0.95225	0.00291	0.54098	0.45663	-0.45169	1	0.19682	-0.11085	0.19	-0.05512	-0.79149	-0.5902	-0.67206	0.51466	-0.67377	-0.67112	-0.68069	-0.64687
**Bio 8**	0.9656	0.22293	0.11723	-0.1804	0.93309	0.7567	0.19682	1	0.91224	0.99761	0.90103	-0.17824	-0.0306	-0.1231	0.22255	-0.12478	-0.10917	-0.44232	-0.06339
**Bio 9**	0.96813	-0.01392	0.29126	-0.53425	0.82233	0.92549	-0.11085	0.91224	1	0.926	0.98298	0.11209	0.31254	-0.0137	0.1777	0.21972	0.00161	-0.2379	0.04547
**Bio 10**	0.97647	0.22422	0.14157	-0.22131	0.94117	0.77101	0.19	0.99761	0.926	1	0.91884	-0.16451	0.00719	-0.13881	0.24452	-0.0921	-0.12499	-0.45016	-0.07773
**Bio 11**	0.97993	0.0603	0.36947	-0.58725	0.84865	0.90119	-0.05512	0.90103	0.98298	0.91884	1	0.03686	0.32799	-0.14482	0.31227	0.21395	-0.13131	-0.29531	-0.08977
**Bio 12**	-0.09085	-0.79012	-0.16696	-0.44538	-0.35819	0.36075	-0.79149	-0.17824	0.11209	-0.16451	0.03686	1	0.74661	0.78395	-0.59629	0.8568	0.78864	0.8057	0.79173
**Bio 13**	0.17075	-0.47217	0.26929	-0.79901	-0.06347	0.47321	-0.5902	-0.0306	0.31254	0.00719	0.32799	0.74661	1	0.21402	0.05333	0.97423	0.22097	0.56417	0.23005
**Bio 14**	-0.18551	-0.78698	-0.52331	0.04959	-0.39353	0.21668	-0.67206	-0.1231	-0.0137	-0.13881	-0.14482	0.78395	0.21402	1	-0.90996	0.37929	0.99428	0.65615	0.97676
**Bio 15**	0.32412	0.63768	0.51856	-0.24585	0.4597	-0.00714	0.51466	0.22255	0.1777	0.24452	0.31227	-0.59629	0.05333	-0.90996	1	-0.12655	-0.91121	-0.49282	-0.88734
**Bio 16**	0.05542	-0.58237	0.16271	-0.72395	-0.19376	0.41856	-0.67377	-0.12478	0.21972	-0.0921	0.21395	0.8568	0.97423	0.37929	-0.12655	1	0.38369	0.6864	0.3847
**Bio 17**	-0.17178	-0.78359	-0.51506	0.04494	-0.38013	0.22926	-0.67112	-0.10917	0.00161	-0.12499	-0.13131	0.78864	0.22097	0.99428	-0.91121	0.38369	1	0.65575	0.98456
**Bio 18**	-0.39853	-0.70063	-0.20072	-0.19125	-0.59761	0.01986	-0.68069	-0.44232	-0.2379	-0.45016	-0.29531	0.8057	0.56417	0.65615	-0.49282	0.6864	0.65575	1	0.63796
**Bio 19**	-0.12627	-0.76037	-0.52173	0.03938	-0.32882	0.25865	-0.64687	-0.06339	0.04547	-0.07773	-0.08977	0.79173	0.23005	0.97676	-0.88734	0.3847	0.98456	0.63796	1

Note: Color labeled rectangles indicates Pearson correlations (absolute values) that are greater than 0.8. Bio 1 = Annual Mean Temperature (℃); Bio 2 = Mean Diurnal Range (Mean of monthly (max temp - min temp) (℃); Bio 3 = Isothermality (Bio2 / Bio 7) (×100); Bio 4 = Temperature Seasonality (standard deviation ×100) ; Bio 5 = Max Temperature of Warmest Month (℃); Bio 6 = Min Temperature of Coldest Month (℃); Bio 7 = Temperature Annual Range (Bio 5 – Bio 6) (℃); Bio 8 = Mean Temperature of Wettest Quarter (℃); Bio 9 = Mean Temperature of Driest Quarter (℃); Bio 10 = Mean Temperature of Warmest Quarter (℃); Bio 11 = Mean Temperature of Coldest Quarter (℃); Bio 12 = Annual Precipitation (mm); Bio 13 = Precipitation of Wettest Month (mm); Bio 14 = Precipitation of Driest Month (mm); Bio 15 = Precipitation Seasonality (Coefficient of Variation) (mm); Bio 16 = Precipitation of Wettest Quarter (mm); Bio 17 = Precipitation of Driest Quarter (mm); Bio 18 = Precipitation of Warmest Quarter (mm); Bio 19 = Precipitation of Coldest Quarter (mm).

Supplementary Table 10 | Statistics of paired *t*-tests for scaled ecological valence of six Malagasy baobabs.

	A. za	***A. suarezensis***	***A. rubrostipa***	***A. perrieri***	***A. madagascariensis***
A. grandidieri	**0.0002381	*0.01534	*0.1183	0.1276	**0.00599
A. madagascariensis	0.3777	**0.0001396	*0.03732	*0.01238	
A. perrieri	*0.0161	**0.00801	0.8534		
A. rubrostipa	**0.001107	**0.00007605			
A. suarezensis	**0.000009329				

Note: *$p < 0.05$, **$p < 0.01$

Supplementary Table 11 | The global sea level and land area of Madagascar in history.

Global mean sea level (m)	Land area (km)	Changes (%)
-123.63	701498.0246	18.75
-115.63	700254.4081	18.54
-110.03	699211.4864	18.36
-105.23	698240.5376	18.20
-100.43	697198.333	18.02
-95.63	696151.7306	17.84
-90.83	695004.976	17.65
-85.23	693533.6541	17.40
-80.02	691753.5792	17.10
-75.54	689810.7161	16.77
-70.03	686918.3183	16.28
-65.19	684015.8068	15.79
-60.09	680191.0995	15.14
-55.06	675791.7267	14.40
-50.03	671435.0457	13.66
-45.01	666902.9961	12.89
-40.01	662308.1932	12.11
-35.04	656669.7366	11.16
-30.03	648794.8965	9.83
-25.02	639458.6763	8.25
-20.01	626683.4075	6.08
-15.02	613848.501	3.91
-10	603999.0558	2.24
-5	596288.4017	0.94
0	590750	0.00
5	584156.903	-1.12
10.01	571390.5317	-3.28
15.1	561431.118	-4.96
20	553177.5656	-6.36
25.01	545429.0733	-7.67
30.35	537505.0356	-9.01
36.58	530477.612	-10.54

Note: The land area was calculated with reference to the current simulation of global elevation data downloaded from Global Multi-Resolution Topography Data Synthesis (https://www.gmrt.org/).
