# Supplementary material for: The rise of baobab trees in Madagascar
Source: Nature. 2024 May 15;629(8014):1091–9. doi: 10.1038/s41586-024-07447-4 (PMC11136661; doi:10.1038/s41586-024-07447-4)
Supplement: Supplementary file 2 — Reporting Summary [file 41586_2024_7447_MOESM2_ESM.pdf]

nature portfolio

Corresponding author(s): Qing-Feng Wang

Last updated by author(s): Mar 5, 2024

Reporting Summary

Nature Portfolio wishes to improve the reproducibility of the work that we publish. This form provides structure for consistency and transparency in reporting. For further information on Nature Portfolio policies, see our Editorial Policies and the Editorial Policy Checklist.

Statistics

For all statistical analyses, confirm that the following items are present in the figure legend, table legend, main text, or Methods section.

n/a	Confirmed	
☒	☐	The exact sample size (*n*) for each experimental group/condition, given as a discrete number and unit of measurement
☒	☐	A statement on whether measurements were taken from distinct samples or whether the same sample was measured repeatedly
☐	☒	The statistical test(s) used AND whether they are one- or two-sided<br>*Only common tests should be described solely by name; describe more complex techniques in the Methods section.*
☒	☐	A description of all covariates tested
☒	☐	A description of any assumptions or corrections, such as tests of normality and adjustment for multiple comparisons
☐	☒	A full description of the statistical parameters including central tendency (e.g. means) or other basic estimates (e.g. regression coefficient) AND variation (e.g. standard deviation) or associated estimates of uncertainty (e.g. confidence intervals)
☐	☒	For null hypothesis testing, the test statistic (e.g. *F*, *t*, *r*) with confidence intervals, effect sizes, degrees of freedom and *P* value noted<br>*Give P values as exact values whenever suitable.*
☒	☐	For Bayesian analysis, information on the choice of priors and Markov chain Monte Carlo settings
☒	☐	For hierarchical and complex designs, identification of the appropriate level for tests and full reporting of outcomes
☒	☐	Estimates of effect sizes (e.g. Cohen's *d*, Pearson's *r*), indicating how they were calculated

Our web collection on statistics for biologists contains articles on many of the points above.

Software and code

Policy information about availability of computer code

Data collection	No software used for data collection.
Data analysis	K-mer analysis on genome size estimation: SOAPnuke (v1.5.6); Jellyfish (v2.3.0); Genomescope1.0; Genomescope 2.0; Smudgeplot (v0.2.5).<br>Genome assembly and contigs anchoring: Falcon (v0.5); Pilon (v1.23); Hifisam (v0.15.1); bwa (v0.7.12); Juicer pipeline (v1.5); 3d-dna (version 180922); JuiceBox (v1.9.0); RepBase (v21.12).<br>Genome annotation and assessment: RepBase (v21.12); RepeatModeler (v2.0); LTR_Finder (v1.07); REPEATMASKER (v3.3.0); Tandem Repeats Finder (v4.09); hista2 (v2.1.0); stringtie (v2.1.6); GeMoMa (v1.6.4); AUGUSTUS (v3.2.3); SNAP (version 2006-07-28); Maker-P (v2.31.11).<br>Historical activity of LTR retrotransposons: LTR_Finder (v1.0.7); Genome Tools (v1.5.8); LTR_retriever (v1.9); TEsorter (v1.3.0).<br>Syntenic analysis and whole genome duplication detection: JCVI (v1.1.11); MCScanX-170908; PAML (v4.5).<br>Orthologous gene set construction: BLASTP (2.9.0+); OrthoMCL (version 1.4).<br>Phylogenetic inference and molecular dating: MUSCLE (v3.8.31); MrBayes (v3.1.2); PAML (v4.5); RAxML-8.2.11; ASTRAL-5.6.1; LASTZ 1.04; MULTIZ (v11.2); CONSEL (v1.20); NOVOPlasty (v4.3.1).<br>Variant calling and filtering: bwa (v0.7.17); samtools (v1.9); Picard (v2.21.6); GATK (v4.1.2.0).<br>Identification of copy number variations: CNVpytor (v1.2.1); R package “ape” (version 5.6-2).<br>Gene flow: Dsuite (version 0.5 r47); genomics_general-master2 (v0.4); QuIBL (https://github.com/miriammiyagi/QuIBL); PhyloNet (v3.8.2).<br>Genome wide heterozygosity estimation: MlRho (v2.9).<br>Demographic inference and ROH estimation: PSMC (version 0.6.5-r67).<br>Coupled logistic modeling: R package “deSolve” (version 1.35).<br>Species distribution data: Global Biodiversity Information Facility (https://www.gbif.org/; November 28, 2022).<br>Environmental variables: WorldClim database version 2.0 (www.worldclim.org); ArcGIS 10.2.<br>Modeling procedure: MaxEnt (v3.4.1).

April 2023

Paleo-distribution prediction by ecological niche consistency: ArcGIS 10.2.
The analysis of niche overlap: R package "ecospat" (version 3.4).
The geological history of Madagascar: Global Elevation Layer (https://www.gmrt.org/); ArcGIS 10.2.

For manuscripts utilizing custom algorithms or software that are central to the research but not yet described in published literature, software must be made available to editors and reviewers. We strongly encourage code deposition in a community repository (e.g. GitHub). See the Nature Portfolio guidelines for submitting code & software for further information.

Data

Policy information about availability of data

All manuscripts must include a data availability statement. This statement should provide the following information, where applicable:

- Accession codes, unique identifiers, or web links for publicly available datasets
- A description of any restrictions on data availability
- For clinical datasets or third party data, please ensure that the statement adheres to our policy

The raw and whole genome sequence data reported in this paper have been deposited in the Genome Sequence Archive123 in National Genomics Data Center124, China National Center for Bioinformation / Beijing Institute of Genomics, Chinese Academy of Sciences (GSA: CRA015211) that are publicly accessible at https://ngdc.cncb.ac.cn/gsa. The distribution data of baobabs used in our analyses are freely available in GBIF (www.gbif.org) and the doi representing the download is: https://doi.org/10.15468/dl.ajuz23.
The data sources of outgroup species: Arabidopsis thaliana (https://phytozome-next.jgi.doe.gov/info/Athaliana_TAIR10); Bombax ceiba (https://gigadb.org/dataset/100445); Carica papaya (https://phytozome-next.jgi.doe.gov/info/Cpapaya_ASGPBv0_4); Durio zibethinus (https://www.ncbi.nlm.nih.gov/assembly/GCF_002303985.1); Gossypium arboreum (https://www.cottongen.org/species/Gossypium_arboreum/A2_WHU); Gossypium raimondii (https://www.cottongen.org/species/Gossypium_raimondii/NSF-D5); Theobroma cacao (https://cocoa-genome-hub.southgreen.fr/download); Populus trichocarpa (https://phytozome-next.jgi.doe.gov/info/Ptrichocarpa_v3_1).

Research involving human participants, their data, or biological material

Policy information about studies with human participants or human data. See also policy information about sex, gender (identity/presentation), and sexual orientation and race, ethnicity and racism.

Reporting on sex and gender	N/A
Reporting on race, ethnicity, or other socially relevant groupings	N/A
Population characteristics	N/A
Recruitment	N/A
Ethics oversight	N/A

Note that full information on the approval of the study protocol must also be provided in the manuscript.

Field-specific reporting

Please select the one below that is the best fit for your research. If you are not sure, read the appropriate sections before making your selection.

☒ Life sciences ☐ Behavioural & social sciences ☐ Ecological, evolutionary & environmental sciences

For a reference copy of the document with all sections, see nature.com/documents/nr-reporting-summary-flat.pdf

Life sciences study design

All studies must disclose on these points even when the disclosure is negative.

Sample size	No statistical method was used for determining sample size. One individual of each species was sequenced.
Data exclusions	The tetraploid species A. digitata was only excluded from the genome-wide estimation of theta and ROH, and the analysis of PSMC, due to the incommensurability of these estimations for species have a higher polyploid level.
Replication	No such experiments.
Randomization	The sampling of individual for sequencing was randomly conducted without any specific selection. No group allocation was needed.
Blinding	Blinding was not relevant to our study as comparison were performed by computer software not influenced by investigator.

Reporting for specific materials, systems and methods

We require information from authors about some types of materials, experimental systems and methods used in many studies. Here, indicate whether each material, system or method listed is relevant to your study. If you are not sure if a list item applies to your research, read the appropriate section before selecting a response.

Materials & experimental systems

n/a	Involved in the study	
☒	☐	Antibodies
☒	☐	Eukaryotic cell lines
☒	☐	Palaeontology and archaeology
☒	☐	Animals and other organisms
☒	☐	Clinical data
☒	☐	Dual use research of concern
☐	☒	Plants

Methods

n/a	Involved in the study	
☒	☐	ChIP-seq
☒	☐	Flow cytometry
☒	☐	MRI-based neuroimaging

Dual use research of concern

Policy information about dual use research of concern

Hazards

Could the accidental, deliberate or reckless misuse of agents or technologies generated in the work, or the application of information presented in the manuscript, pose a threat to:

No	Yes	
☒	☐	Public health
☒	☐	National security
☒	☐	Crops and/or livestock
☒	☐	Ecosystems
☒	☐	Any other significant area

Experiments of concern

Does the work involve any of these experiments of concern:

No	Yes	
☒	☐	Demonstrate how to render a vaccine ineffective
☒	☐	Confer resistance to therapeutically useful antibiotics or antiviral agents
☒	☐	Enhance the virulence of a pathogen or render a nonpathogen virulent
☒	☐	Increase transmissibility of a pathogen
☒	☐	Alter the host range of a pathogen
☒	☐	Enable evasion of diagnostic/detection modalities
☒	☐	Enable the weaponization of a biological agent or toxin
☒	☐	Any other potentially harmful combination of experiments and agents

Plants

Seed stocks	Young leaves of A. digitata and A. gregorii were collected from trees in wild populations. The ripe fruits of Malagasy baobabs were collected from natural populations and seeds were further germinated for fresh young leaves at a local nursery garden in Antananarivo, Madagascar. A. digitata Lon:-17.19111, Lat: 14.74497, Senegal; A. gregorii Lon: 132.26266, Lat: -14.45994, Australia; A. grandidieri Lon: 44.415654, Lat: -20.255134, Madagascar, 2021-11-05; A. madagascariensis Lon: 49.11928, Lat: -12.96816, Madagascar, 2021-11-05; A. perrieri Lon: 49.171495, Lat: -12.490713, Madagascar, 2021-11-05; A. rubrostipa Lon: 43.6290503, Lat: -23.1193637, Madagascar, 2021-11-16; A. suarezensis Lon: 49.16806, Lat: -12.26667, Madagascar, 2021-11-16; A. za Lon: 44.534665, Lat: -20.335426, 2021-11-05.
Novel plant genotypes	No novel plant genotype.
Authentication	No such experiments.
